# Synthesis of Shape-Memory Polyurethanes: Combined Experimental and Simulation Studies

**DOI:** 10.3390/ijms23137064

**Published:** 2022-06-25

**Authors:** Karolina Rolińska, Magdalena Mazurek-Budzyńska, Paweł G. Parzuchowski, Dominik Wołosz, Maria Balk, Krzysztof Gorący, Miroslawa El Fray, Piotr Polanowski, Andrzej Sikorski

**Affiliations:** 1 Faculty of Chemistry, Warsaw University of Technology, Noakowskiego 3, 00-664 Warsaw, Poland; magdalena.budzynska@pw.edu.pl (M.M.-B.); pawel.parzuchowski@pw.edu.pl (P.G.P.); dominik.wolosz.dokt@pw.edu.pl (D.W.); 2 Faculty of Chemistry, University of Warsaw, Pasteura 1, 02-093 Warsaw, Poland; sikorski@chem.uw.edu.pl; 3 Institute of Active Polymers, Helmholtz-Zentrum Hereon, Kantstraße 55, 14513 Teltow, Germany; maria.balk@hereon.de; 4 Department of Polymer and Biomaterials Science, Faculty of Chemical Technology and Engineering, West Pomeranian University of Technology, Piastów Avenue 42, 71-065 Szczecin, Poland; kgoracy@zut.edu.pl (K.G.); mirfray@zut.edu.pl (M.E.F.); 5 Faculty of Chemistry, Technical University of Lodz, Zeromskiego 116, 90-924 Lodz, Poland; piotr.polanowski@p.lodz.pl

**Keywords:** oligocarbonate diols, poly(carbonate-urethane-urea)s, shape-memory, dynamic liquid lattice, Monte Carlo

## Abstract

The presented research focuses on the synthesis and structure–properties relationship of poly(carbonate-urea-urethane) (PCUU) systems including investigations on shape-memory effect capability. Furthermore, we approached the topic from a broader perspective by conducting extensive analysis of the relationship between the synthesized compounds and the results of computer simulations by means of the Monte Carlo method. For the first time, by using a unique simulation tool, the dynamic lattice liquid model (DLL), all steps of multi-step synthesis of these materials were covered by the simulations. Furthermore, broad thermal, mechanical, and thermomechanical characterization of synthesized PCUUs was performed, as well as determining the shape-memory properties. PCUUs exhibited good mechanical properties with a tensile strength above 20 MPa, elongation at break around 800%, and an exhibited shape-memory effect with shape fixity and shape recovery ratios above 94% and 99%, respectively. The dynamic lattice liquid model was employed to show the products and their molar mass distribution, as well as monomer conversion or the dispersity index for individual reaction steps. The results obtained in the following manuscript allow the planning of syntheses for the PCUUs of various structures, including crosslinked and soluble systems, which can provide a broad variety of applications of these materials, as well as a better understanding of the composition–properties relationship.

## 1. Introduction

With a worldwide production of nearly 25 Mt in 2021, polyurethanes (PURs) are the sixth most widely used polymers in the world and the global market for these polymers is going to grow in the next five years by 6.8% [1]. The high demand and popularity of PURs originate from their excellent physical and mechanical properties such as toughness, durability, elasticity, and abrasive resistance. They are used for the production of elastic and rigid foams, seals, high-performance coatings, and adhesives [2,3,4]. Due to excellent biocompatibility and biostability, PURs have also found application as biomedical materials [5,6,7,8].

Conventional PURs are obtained from diisocyanates or polyisocyanates, polyols, and chain extenders. The desired properties of PURs are achievable by appropriate selection of the reacting components, which makes them highly versatile materials. Applications of polyester diols as soft segments of PURs enable preparation of polymers of relatively good physical properties. However, they are susceptible to hydrolytic degradation of the ester linkages [9]. On the other hand, PURs based on polyether diols exhibit better hydrolytic resistance and are therefore favored in the applications where hydrolytic stability is expected. Unfortunately, the poly(ether-urethane)s are susceptible to oxidation processes. Both of mentioned disadvantages can be overcome by replacing polyester or polyether segments by hydrolytically and oxidatively stable polycarbonate ones [10,11].

Synthesis of poly(carbonate-urethane)s (PCURs) requires appropriate soft segment precursors, oligocarbonate diols (OCDs). Aliphatic OCDs can be obtained by catalytic co-polymerization of oxiranes with carbon dioxide [12], ring-opening polymerization of six- and seven-membered cyclic carbonates [13], or in polycondensation processes using simple organic carbonates such as a linear dimethyl carbonate (DMC) or cyclic ethylene or propylene carbonates (EC or PC) and α,ω-diols or α,ω-diols mixtures. In the latter case, the use of DMC requires a two-step procedure leading to hydroxyl terminated oligomerols, while the use of EC or PC is a one-step reaction. Both methods allow OCDs of molar mass to be obtained in the range of 500 g × mol^−1^ to several thousand g × mol^−1^, depending on the type of α,ω-diol used [14,15]. The advantage of the OCD synthesis involving DMC is the lower reaction temperature, that is, the boiling point of DMC/methanol azeotrope, and the possibility of carrying out the reaction without the addition of organic solvents. Due to the direct or indirect use of carbon dioxide in their synthesis, OCDs are also considered to be environmentally friendly “green starting materials”. Furthermore, PCURs, owing to their superior mechanical and biological properties, as well as being environmentally friendly starting materials, are favored as biopolymers for long-lasting implantation such as in the spine, as meniscus implants or as artificial heart elements [16,17,18]. What is more, the above-mentioned features of PCURs have also attracted attention in the field of shape-memory polymers (SMPs). Recently, we proposed the method of preparation of SMPs that involved the utilization of OCDs containing long hydrocarbon chains between the carbonate linkages. Obtained poly(carbonate-urea-urethane) networks (PCUUs) exhibited a high-strain shape-memory effect with excellent shape recovery and temporary shape fixation ratios [19,20], as well as a superb reversible bidirectional shape-memory effect [21].

Segmented thermoplastic PURs have been investigated for many years [22,23,24,25,26]. In 1981, Castro et al. detected early phase separation in PURs using light transmission and viscosity measurements [27]. Yilgör et al. concluded that the improvement in microphase separation was related to the presence of urea hard segments, particularly when using symmetrical diisocyanates [28]. The research of Sun and Sung [29] on methylene diphenyl diisocyanate (MDI) and 1,4-butandiol reactions confirmed the equal reactivity of both isocyanate groups in MDI. Several research groups have also been working on the incorporation of computer simulations for a better understanding of PUR synthesis, structure, and properties. In 1990, Lee and Eichinger used computer simulations to model the structure and elasticity of PUR networks [30,31]. Monte Carlo and molecular dynamics simulations at the atomistic level were successfully implemented to study the structure and mechanics of heterogeneous block PURs [32]. Density functional theory (DFT) study of the phase-separation morphology of urea/urethane-based segmented thermoplastic copolymers were also performed [33]. The thermomechanical constitutive model of shape-memory PURs was proposed by using a non-linear constitutive equation. The parameters such as shape fixity and shape recovery ratios were determined in the proposed method [34]. Recently, a thermo-mechanical behavior of shape-memory PUR copolymers was reported in the literature. Park et al. simulated the SMP using a coarse-grained molecular dynamics simulation [35]. However, to our knowledge, no research concerning computer simulations of complex two-step OCD synthesis, followed by the synthesis of urethane prepolymers and curing process of PCUUs has been carried out before.

This confirms the legitimacy of pursuing studies based on experimental data supported by the theoretical approach. Computer simulations of such processes are usually studied by using the molecular dynamics [36], dissipative particle dynamics [37,38], and Monte Carlo (MC) methods [39,40,41,42,43,44]. One type of MC simulation is based on the dynamic lattice liquid (DLL) model, which assumes that a molecular transport is realized in a cooperative motion of the objects in the lattice that moves in the closed loops. In this algorithm, some additional assumptions are made, such as it being an athermal system (exclusion of volume effects only) and disregarding the fact that chain structure influences chain reactivity. On the other hand, proper dynamics of complex macromolecular systems have been provided by this model [45]. The other advantage of the application of the proposed method is the possibility of studies on polymer systems with high densities, which should resemble a real crowded environment [46]. The DLL model was already employed for studies on atom transfer radical polymerization, showing good agreement with the experimentally determined gel points [46], and for polymerization of various macromolecular systems such as star-branched polymers [47,48], polymer brushes [49], and opposing polymer brushes [50,51].

Based on these findings, we decided to perform computer simulations of the complex preparation process of PCUUs based on MC simulations, which includes two-step synthesis of OCDs, synthesis of urethane prepolymers, and the curing step. The DLL model is an efficient tool, which enables the simulations of dense systems that are suitable for investigation of multi-step synthesis of the polymers. In order to set the tractable model, we assumed that distances between reacting components and topologies, conformations, and the diffusion of the elements are not taken into consideration. In the discussed model there is also a possibility of the presumption of cross-linking reactions during the process [52,53]. Furthermore, detailed characterization of thermal, mechanical, and thermomechanical properties, followed by the shape-memory investigations of obtained PCUUs were performed.

## 2. Materials and Methods

### 2.1. Materials

The 1,10-decanediol (purity 98%) and isophorone diisocyanate (IPDI) (purity 98%) were purchased from Sigma-Aldrich (Poznan, Poland). Potassium carbonate (K_2_CO_3_) (purity ≥ 99%), 1,4-dioxane (purity 99%), dimethyl carbonate (purity 99%), and chloroform (purity ≥ 99%) were purchased from POCH (Gliwice, Poland). The materials were used without any further purification.

### 2.2. Syntheses

#### 2.2.1. Bis(methylcarbonate)decamethylene

##### Bis(methylcarbonate)decamethylene Based on 1,10-Decanediol with an 8 Molar Excess of DMC (E_BMC_8)

The alkylene bis(methylcarbonate)s (BMCs) were synthesized according to previously published procedures [20]. Briefly, in a 2000 cm^3^ reactor equipped with a magnetic stirrer, Vigreux column, distillation condenser, and thermometer, 250.0 g (1.43 mol) of 1,10-decanediol and 1000.0 g (11.1 mol) of DMC were placed in the presence of potassium carbonate (3 mol.% referred to 1,10-decanediol), used as a catalyst. The mixture was heated under reflux for 1.5 h at 80 °C. Thereafter, while using the distillation column, the azeotrope of DMC/methanol was removed. The reactions were carried out at the boiling point of the mixture (approximately 90 °C). The progress of the reaction was followed by measurement of refractive index of the distillate until no more methanol was detected. In addition, the region from 3.85 ppm to 3.55 ppm in the ^1^H NMR spectra was analyzed to observe the progress of conversion for E_BMC_10 with 20 min sampling intervals (Figure S1, Supplementary Materials). The excess of DMC and residuals of methanol were removed under reduced pressure. Yield was 99%.

E_BMC_8: ^1^H NMR (CDCl_3_, 400MHz): δ (ppm) = 4.11 (t, 4H, C(O)OCH_2_), 3.76 (s, 6H, CH_3_O), 1.63 (m, 4H, OCH_2_CH_2_CH_2_CH_2_CH_2_), 1.33 (m, 4H, OCH_2_CH_2_CH_2_CH_2_CH_2_), 1.26 (m, 8H, OCH_2_CH_2_CH_2_CH_2_CH_2_); Figure S1, Supplementary Materials.

##### BMC Based on 1,10-Decanediol with a 10 Molar Excess of DMC (E_BMC_10)

E_BMC_10 was obtained analogically to E_BMC_8, using 200.0 g (1.15 mol) of 1,10-decanediol and 1000.0 g (11.1 mol) of DMC. Yield 98%.

E_BMC_10: ^1^H NMR (CDCL_3_, 400MHz): δ (ppm) = 4.12 (t, 4H, C(O)OCH_2_), 3.77 (s, 6H, CH_3_O), 1.65 (m, 4H, OCH_2_CH_2_CH_2_CH_2_CH_2_), 1.34 (m, 4H, OCH_2_CH_2_CH_2_CH_2_CH_2_), 1.28 (m, 8H, OCH_2_CH_2_CH_2_CH_2_CH_2_); Figure S2, Supplementary Materials.

##### BMC Based on 1,10-Decanediol with an 18 Molar Excess of DMC (E_BMC_18)

E_BMC_18 was obtained analogically to E_BMC_8 and E_BMC_10, using 105.0 g (0.6 mol) of 1,10-decanediol and 1000.0 g (11.1 mol) of DMC. Yield 98%.

E_BMC_18: ^1^H NMR (CDCl_3_, 400MHz): δ (ppm) = 4.12 (t, 4H, C(O)OCH_2_), 3.77 (s, 6H, CH_3_O), 1.65 (m, 4H, OCH_2_CH_2_CH_2_CH_2_CH_2_), 1.34 (m, 4H, OCH_2_CH_2_CH_2_CH_2_CH_2_), 1.27 (m, 8H, OCH_2_CH_2_CH_2_CH_2_CH_2_); Figure S3, Supplementary Materials.

FTIR (ATR): 2920, 2850, 1750, 1440, 1280, 1260, 950, 930, 790, 710 cm^−1^; Figures S4–S6 in the Supplementary Materials.

#### 2.2.2. Oligo(decamethylene carbonate) Diols

##### Oligo(decamethylene carbonate) Diol with an Average Molar Mass of 3000 g × mol^−1^ (E_OCD_3000)

The 56.5 g of 1,10-decanediol (0.32 mol), 150.0 g of E_BMC_8 (0.52 mol) and K_2_CO_3_ (0.005 mol.%) were placed in a 500 cm^3^ three-necked flask equipped with a magnetic stirrer, thermometer, and distillation condenser. Then, approximately 60 cm^3^ of 1,4-dioxane was added. The temperature of the mixture was gradually increased from 100 °C to 160 °C. The progress of the reaction was monitored by measuring the refractive index of the distillate and carried out until no methanol was observed in the distillate (around 14 h). Afterwards, the reaction was continued under reduced pressure at 160 °C (for an additional 4 h), while the 1,10-decanediol and residual 1,4-dioxane were removed. The obtained product was dissolved in dichloromethane and washed six times with demineralized water to remove the catalyst. Afterwards, the solvent and residuals of water were removed under reduced pressure. The synthesized product was characterized by ^1^H NMR and FT-IR spectroscopy. Based on ^1^H NMR, the number average molar mass was calculated to be equal 3000 g × mol^−1^.

E_OCD_3000: ^1^H NMR: (CDCl_3_, 400MHz): δ (ppm)= 4.10 (t, 4H, C(O)OCH_2_), 3.62 (t, 4H, CH_2_OH), 1.65 (m, 4H, HOCH_2_CH_2_CH_2_CH_2_CH_2_), 1.55 (m, 4H, HOCH_2_CH_2_CH_2_CH_2_CH_2_), 1.53 (m, 4H, OCH_2_CH_2_CH_2_CH_2_CH_2_), 1.32 (m, 4H, OCH_2_CH_2_CH_2_CH_2_CH_2_), 1.27 (m, 4H, OCH_2_CH_2_CH_2_CH_2_CH_2_); Figure S7, Supplementary Materials.

FT-IR (ATR): 3450, 2920, 2850, 1740, 1470, 1400, 1340, 1280, 1250, 1030, 940, 790 cm^−1^; Figure S8, Supplementary Materials.

##### Oligo(decamethylene carbonate) Diol with a Number Average Molar Mass of 5000 g × mol^−1^ (E_OCD_5000) after the Described Synthesis Step

Synthesis of E_OCD_5000 was similar to E_OCD_3000. In this synthesis, 23.0 g (0.13 mol) of 1,10-decanediol and 50.0 g of E_BMC_8 (0.17 mol) were used, and the time required for the whole synthesis was 20 h. Number average molar mass was calculated based on ^1^H NMR (M_n_ = 5000 g × mol^−1^).

E_OCD_5000: ^1^H NMR: (CDCl_3_,400MHz): δ (ppm)= 4.10 (t, 4H, C(O)OCH_2_), 3.63 (t, 4H, CH_2_OH), 1.65 (m, 4H, HOCH_2_CH_2_CH_2_CH_2_CH_2_), 1.56 (m, 4H, HOCH_2_CH_2_CH_2_CH_2_CH_2_), 1.54 (m, 4H, OCH_2_CH_2_CH_2_CH_2_CH_2_), 1.34 (m, 4H, OCH_2_CH_2_CH_2_CH_2_CH_2_), 1.27 (m, 4H, OCH_2_CH_2_CH_2_CH_2_CH_2_); Figure S9, Supplementary Information.

FT-IR (ATR): 3450, 2920, 2850, 1740, 1470, 1400, 1340, 1280, 1250, 1030, 940, 790 cm^−1^; Figure S10, Supplementary Materials.

#### 2.2.3. Synthesis of Urethane Prepolymers PCUUs

A total of 20.00 ± 0.15 g of OCD was placed in the reaction flask equipped with a thermometer and mechanical stirrer, and it was dried under reduced pressure at 90 °C for 1.5 h. Afterwards, IPDI was added in various molar ratios relative to OCD and the reaction was continued at 80 °C [15].

FT-IR (KBr): 3670, 3380, 2960, 2910, 2260, 1750, 1720, 1240, 790 cm^−1^. Figures S11–S17 in the Supplementary Materials.

#### 2.2.4. Chain Extending of the PCURs Prepolymers with Water Vapor–Formation of PCUUs

The obtained prepolymer was poured into an open glass mold (10 cm × 10 cm) and placed in a climatic chamber at 75 °C and 5% relative humidity for 1 day, then at 70 °C and 10% relative humidity for a further 4 days, and continuing at 60 °C and 40% relative humidity for 2 days. The obtained samples were named E_PCUUX_Y for PCUU based on OCD with the number average molar mass (M_n_) equal to X g × mol^−1^ and a molar excess of IPDI to OCD used during the synthesis equal to Y. Accordingly, the sample named E_PCUU5000_3 was used for experimental PCUU based on OCD with the number average molar mass of 5000 g × mol^−1^ and a molar excess of IPDI equal to 3. The ^1^H NMR spectra of the obtained PCUUs are shown in Figures S18–S21, Supplementary Materials.

FT-IR (ATR): 3670, 3380, 2960, 2910, 1740, 1720, 1240, 790, 720, 430 cm^−1^. Figures S22–S28 in the Supplementary Information.

FTIR (transmittance): 3620, 3350, 2920, 2850, 1630, 1560, 1470, 1410, 1260, 1250, 950, 790 cm^−1^. Exemplarily for PCUU3000_3 in Figure S24 in the Supplementary Materials.

### 2.3. Characterization Techniques

The ^1^H NMR and ^13^C NMR spectra were recorded in CDCl_3_ on a Varian VXR 400 MHz Spectrometer (Palo Alto, CA, USA) and analyzed with MestReNova v.6.2.0-7238 (Mestrelab Research S.L) software.

Time-of-flight mass spectrometry (MALDI-TOF) measurements were performed on a Bruker UltraFlex MALDI TOF/TOF Spectrometer (Bremen, Germany) in a linear or reflection mode using a DHB (2,5-dihydroxybenzoic acid) matrix and Bruker Peptide Calibration Standard (1047.19–3149.57 Da) as a calibrant, and analyzed with Polymerix v.2.0 (Sierra Analytics Inc.) software. Exemplary table and graph showing the results of the performed experiment are shown in Table S1 and Figure S29, Supplementary Materials.

The GPC analysis was performed using a Malvern Viscotek GPCMax TDA 305 (Malvern Panalytical, Malvern, UK). The chromatograph was equipped with a 30 cm Jordi Gel DVB mixed bed column with an inside diameter of 7.8 mm (Jordi Labs, Mansfield, MA, USA). The system was calibrated with a polystyrene standard. Approximately 2 mg of a solid sample was dissolved in 1.5 mL of dichloromethane. After complete dissolution, the solutions were passed through a syringe filter with a PTFE membrane with a 0.2 µm pore size. The flow rate of dichloromethane was set as 1 mL∙min^−1^ and the temperature was set as 30 °C.

ATR-FT-IR spectra were recorded on a Thermo Scientific Nicolete iS5 FTIR spectrometer using an ATR iD7 accessory. Thirty-two scans were performed for each sample with a resolution of 4 cm^−1^. A sample of PCUU_3000_3 was also measured with the transmittance mode using a very thin film of the sample.

Measurements of differential scanning calorimetry (DSC) were performed with the use of TA Instruments Q1000 DSC. Samples were investigated with a constant heating and cooling rate of 10 °C∙min^−1^ at the temperature range from 90 °C to 150 °C. The purge cell gas was nitrogen with a flow rate of 50mL∙min^−1^. A total of 15–25 mg of each sample was loaded on an aluminium pan sealed with a pinhole cap.

Modulated differential scanning calorimetry (MDSC) was performed using a TA Instruments Q100 DSC at the temperature range from −90 °C to 150 °C. The four runs of heating were performed with each material: first heating run, second heating run of previously heated sample, first heating run of the elongated sample with 100% strain and the second heating run of the previously elongated sample after its return to the primary shape. The sample was loaded on an aluminium pan sealed with a pinhole cap. The heating rate was 3 K × min^−1^, modulation amplitude was 0.48 °C, and modulation period was 60 s.

Dynamic mechanical thermal analyses (DMTA) was performed with a DMTA Q800 machine from TA Instruments, New Castle, DE, USA. Measurements were performed at a fixed frequency of 1 Hz, from –90 °C to 150 °C with a heating rate of 3 K × min^−1^. Loading was carried out in the tensile mode with an amplitude of 20 μm in the controlled force mode.

Tensile tests were performed on an Instron 5566 equipped with a 100 N cell load using the standard dumbbell-shaped samples (ISO 527-2/1BB) cut from films. The average value of the tensile strength (σ), elongation at break (ε) and Young’s modulus (E_Young_) for each type of material was determined from at least five specimens. 

The gel fraction contents (G_f_) and the mass equilibrium degrees of swelling (Q_m_) were calculated based by swelling the films in 500-fold excess (related to the sample weight) of chloroform for 72 h at RT, with subsequent drying overnight. The endpoints of swelling and drying were reached when constant weights were obtained. The G_f_ values were calculated according to Equation (1), where the (m_d_) ratio of non-swollen material to extracted sample (m_ex_) was as follows:

(1) $$G_{f}=\frac{m_{\mathrm{ex}}}{m_{d}}$$

The mass equilibrium degrees of swelling (Q_m_) were calculated based on Equation (2) by comparing weights of the non-swollen samples (m_d_) and those in the swollen state (m_sw_).

(2) $$Q_{m}=\frac{m_{\mathrm{sw}}-m_{0}}{m_{d}}$$

Shape-memory effect was analyzed by means of cyclic, thermomechanical tests. For this purpose, two devices were used: a DMTA Q800 machine (TA Instruments, New Castle, DE, USA) and a tensile tester Z75 (Zwick, Ulm, Germany) equipped with a thermo-chamber (Zwick, Ulm, Germany), Eurotherm control 3508 (Eurotherm Regler, Limburg, Germany) temperature controller, and load cells suitable to determine maximum forces of 200 N. Measurements were conducted with standard samples (ISO 527–2/1BB). Five consecutive cycles consisting of heating, stress loading, relaxation, cooling, stress unloading, relaxation and heating were conducted. Shape-memory performance was evaluated by the shape fixity ratio (R_f_) and shape recovery ratio (R_r_), which stand for the ability of SMPs to fix the temporary deformed shape, and the ability to recover the permanent shape, respectively [54]. The first recorded cycle has been considered as annealing and removing the thermal history of the sample. Therefore, it was not included in the determination of shape-memory parameters. The R_f_ and R_r_ were calculated according to Equations (3) and (4), respectively. The sample of PCUU was deformed to ε_prog_ = 100% at T_prog_ with the elongation speed of 10 mm × min^−1^. After keeping this deformation for 10 min to allow relaxation, the stress was held constant, while the sample was cooled to T_low_ with a cooling rate of 5 K × min^−1^ and elongated to ε_l_. After unloading the stress (σ_0_ = 20 mN), and an equilibration time of 10 min, the temporary shape ε_u_ was fixed. Then, the sample was heated to T_prog_ with a heating rate of 5 K × min^−1^ and kept at this temperature for 20 min, resulting in shape recovery to ε_p_. Afterwards, the next cycle was performed. Based on DSC thermograms, values of T_prog_ and T_low_ were chosen as 58 °C and −5 °C, respectively. Exemplary graphs showing the course of the performed experiments are shown in Figures S29–S35, Supplementary Materials.

(3) $$R_{f}=\frac{\varepsilon_{u}\left(N\right)}{\varepsilon_{\mathrm{prog}}}$$

(4) $$R_{r}=\frac{\varepsilon_{u}\left(N\right)-\varepsilon_{p}\left(N\right)}{\varepsilon_{u}\left(N\right)-\varepsilon_{p}\left(N-1\right)}\cdot 100\%$$

### 2.4. Computer Simulations: The Model and the Method

**Figure 1:**
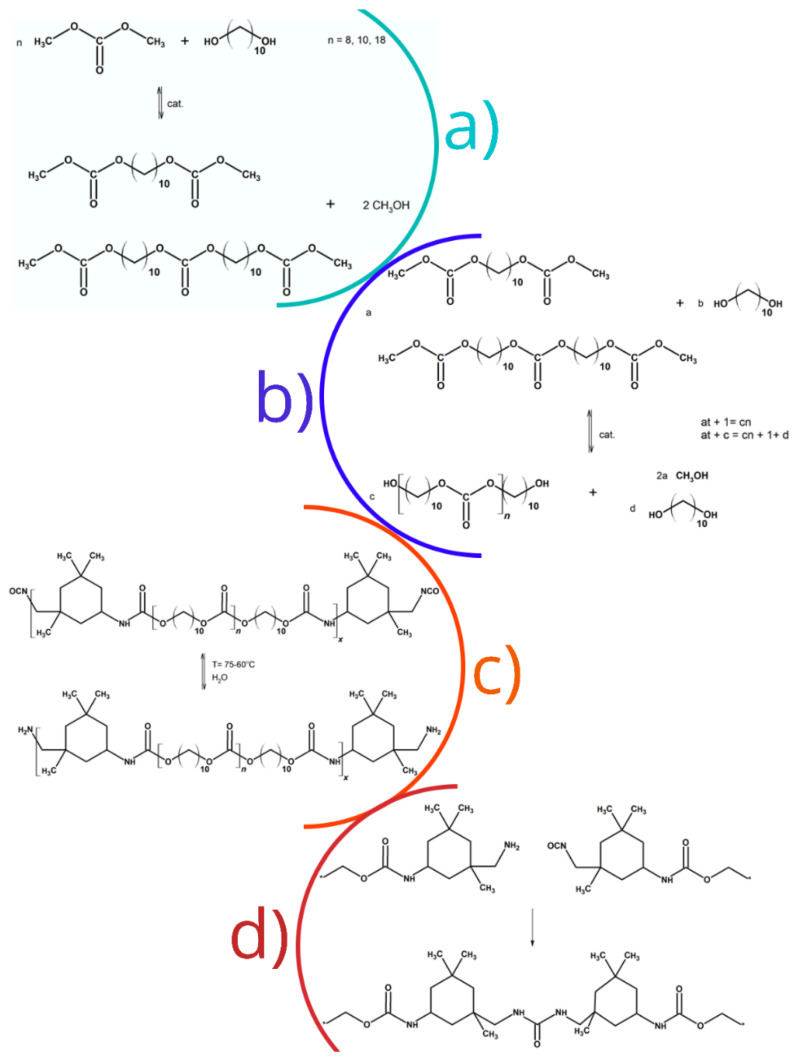
Synthesis steps of: (**a**) alkylene bis(methyl carbonate)s; (**b**) oligo(alkylene carbonate) diols; (**c**) urethane prepolymers; and (**d**) poly(carbonate-urethane-urea)s.

Monte Carlo simulations were employed for better understanding the process of the preparation of PCUU elastomers. The main focus of these computational experiments was the simulation of the process of a two-step synthesis of OCD, followed by the process in which the urethane prepolymers and final cured PCUUs were obtained (Figure 1). The model used in simulations was simplified; all atomic details were ignored and a coarse-grained representation of molecules and macromolecules was assumed. For simplicity, it was assumed that all objects in the system have the same size. These objects (beads) correspond to small molecules or chain fragments and, thus, can be considered as united atoms. The model system was athermal and the excluded volume was the only potential introduced into the system. The excluded volume was realized by prohibiting the double occupancy of lattice sites. The lattice approximation was also assumed in order to speed up the simulations and, therefore, all objects (beads) were put into vertices of a face-centered cubic lattice. The proposed model assumes that the system is completely filled with molecules and macromolecules.

For the simulations, we employed the dynamic lattice liquid (DLL) method in order to solve the adopted model of the process of polymerization. This calculational tool has already been successfully used for studies of polymerization of quite different macromolecular systems, such as star-branched macromolecules and polymer brushes [37,38,39]. The DLL model was described and discussed in detail elsewhere [35,36] and, therefore, we are going to give only a short description here. The model objects occupy all lattice sites in the system and they are not able to move at larger distances due to the crowding caused by the presence of other objects. However, it was assumed that there is a small excess volume, just enough to allow each object to vibrate around its equilibrium position, i.e., around a given lattice site. Sometimes, a long translational motion occurs as a coincidence of attempts of motion of the neighboring objects. The DLL model assumes that a cooperative displacement of the objects has a form of closed loops consisting of at least three objects. All the molecules that do not contribute to cooperative loops of motion at a given time remain at their prior positions. An attempt to simultaneously change the positions of all objects can be regarded as a Monte Carlo step (MCs, a time unit).

The model system was put into a Monte Carlo box with the edge L = 100 lattice units, and, thus, 10^6^ objects were simulated. Periodic boundary conditions were introduced in all directions in order to mimic an infinite system. The concentration of a given object was defined as the ratio of the number of elements to the total number of sites in the system. The appropriate number of elements (beads) was randomly placed in the Monte Carlo box in such a way as to correspond quantitatively to the experimental conditions. Assumptions about reagent ratios were based on stoichiometric calculations and experimental data. The appropriate number of monomer molecules were randomly placed in the Monte Carlo box in such a way as to correspond quantitatively to the experimental conditions. Assumptions about the reagent ratios were based on stoichiometric calculations and experimental data. Furthermore, the elements such as catalyst or solvent were not included directly into the model. The same concerned the resulting by-product, which in the first and second stages was methanol. To obtain stable results, five independent simulation runs for each synthesis stage were performed, which lasted around 1.6 × 10^4^, 6 × 10^5^, 4 × 10^4^, and 4 × 10^7^ MCs steps, respectively, for the first to forth stages of the PCUUs synthesis. The statistical error of calculated parameters in all of the simulated synthesis steps did not exceed 5%. A reaction could occur when a pair of objects were located in neighboring lattice sites. The probability of all reactions that occurred in the system studied was assumed to be 0.02. This assumption was based on our previous simulations of macromolecular systems within the frame of the DLL model [47,48,49], and each reaction took place with a certain probability. As the result of a reaction, the objects changed their identities, and each step of the polymerization process was assumed to be irreversible. We developed a methodology that allowed us to reflect the most important features of the copolymerization process. It assumed that the reactivity of the functional groups was always the same and, thus, it was independent from the chain length. Furthermore, no termination reactions were assumed in the second synthesis step. It has to be noted that there are elements in the system that cannot react with each other as they are terminated with the same end groups.

**Figure 2:**
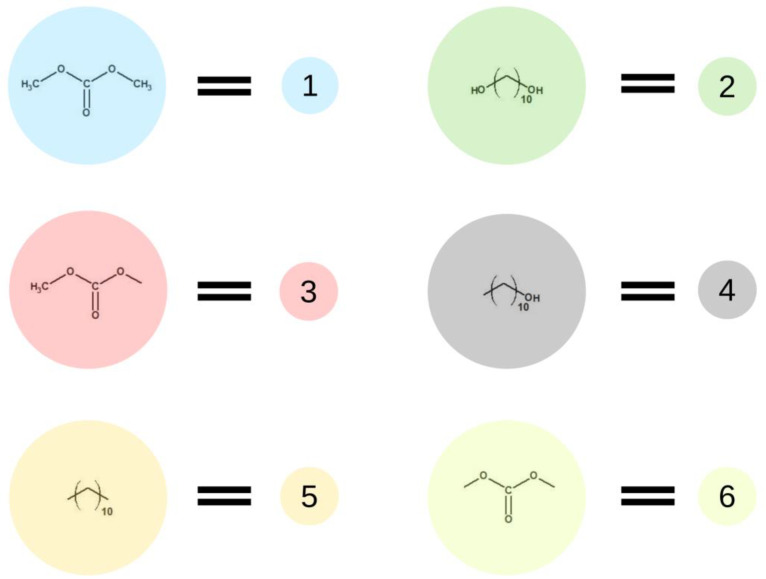
Schematic representation of base units used in the first stage of the simulation.

**Figure 3:**
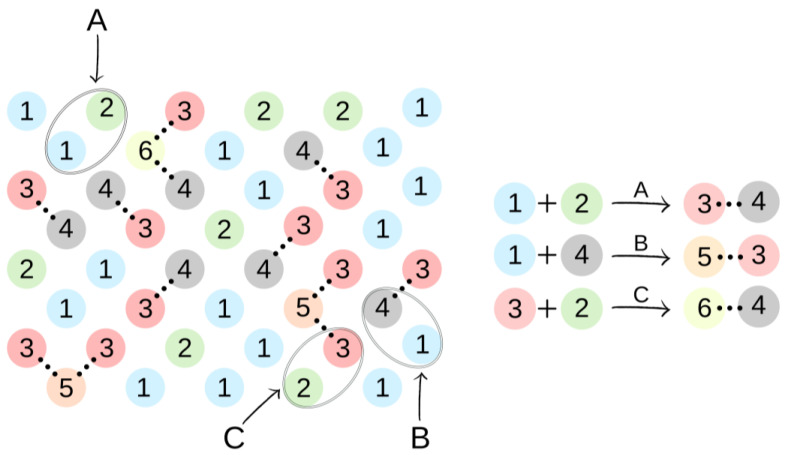
An illustration of the simulation of the synthesis of BMC. The colors and numbers correspond to the objects from Figure 2. The chemical bonds formed in reactions A–C are marked by dotted lines.

Figure 2 presents elements (lattice beads), which represent molecules and their fragments in the first stage of the simulated reaction. A possible representation of reactions of the first step of synthesis using these elements is given in Figure 3.

In the first step of the PCUU synthesis, the reaction between DMC with 1,10-decanediol was simulated. In Figure 2, DMC is illustrated as a bead (1), which, after reaction (depicted as A in Figure 3) with one of -CH_3_ groups, changes into a bead (3), while the second -CH_3_ group remains unreacted. Other elements, which have been assigned with consecutive numbers, have been described similarly. In this work, the numbers in brackets correspond to the molecules, with the numbers described in the schematic graphs. Concentrations of components in the system were assumed to be: 18:1; 10:1, and 8:1, respectively, indicating a molar ratio of DMC to diol. The element (1) was used with a different molar excess over a bead (2), which is a representation of 1,10-decanediol. The element (2) can react with (1) or (3) beads leading to the creation of (4) bead with one unreacted -OH group. Other reactions between the beads are also possible: A: reaction between -CH_3_ and -OH groups of the monomers; B: the reaction between -CH_3_ group in DMC and -OH group of the intermediate product; and C: the reaction between -OH group in diol and -CH_3_ group of the intermediate product. The simulation was carried out until all -OH groups in the system had reacted, i.e., until elements (2) and (4) were converted.

**Figure 4:**
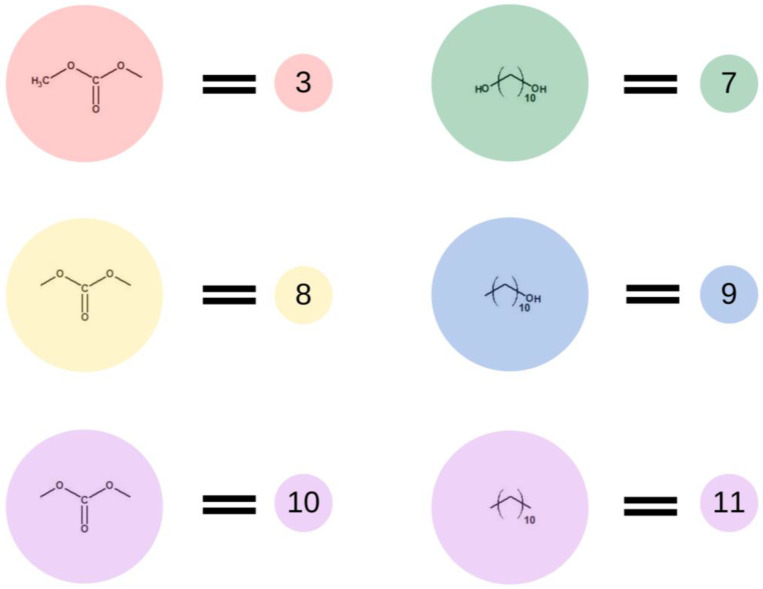
Schematic representation of the base units used in the second stage of the simulation.

**Figure 5:**
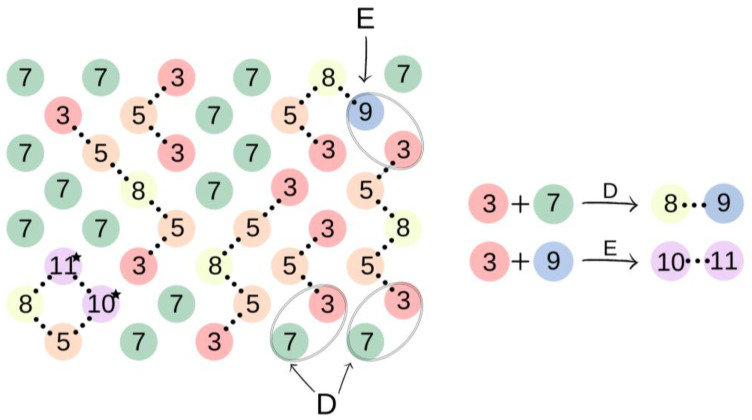
An illustration of the process of oligo(decamethylene carbonate) synthesis. In the case of elements (10) and (11) originated from that same polymeric chain the polymeric ring is formed, which was illustrated with ★ after elements numbers.

Subsequently, in the second step of the simulation process, we studied the synthesis of OCDs, in which the elongation of the polymer chains formed in the previous stage takes place. The product obtained in the first reaction reacted here with 1,10-decanediol. The main advantage of a two-step synthesis is that it allows OCD to be obtained with an estimated molar mass and assures that all the OCD molecules are terminated with hydroxyl groups [15]. In this step, a low molar mass transesterification product formation was ignored. In addition, the preset initial reagent ratios were calculated stoichiometrically. Simulations stopped when OCDs of the desired average chain length were built. This procedure allowed us to avoid simulating the second part of the reaction under reduced pressure, in which 1,10-decanediol was removed from the reaction mixture. Figure 4 presents elements involved in this stage of the reaction. It has to be mentioned that despite the same chemical structure, new markings were introduced in the second stage, which is related to the continuity of the recording, maintaining the appropriate order of insertion and appearance of subsequent elements: element (7) = element (2); element (8) = element (6); element (9) = element (4); element (10) = element (6); and element (11) = element (5). There are also new elements (10) and (11), the appearance of which results in the elongation of the chain. Element (3) is the representation of a bead that ends the BMC chain and has a CH_3_ terminal group. Similarly to the first step of the reaction, 1,10-decanediol was used and was represented as bead (7) to maintain the continuity of the numbering in the proposed coarse-grained model. Figure 5 presents two main reactions: D: the reaction between OH group in diol and -CH_3_ group of BMC; and E: -CH_3_ group of BMC and -OH groups of an already reacted chain. The reaction between (3) and (7), and (3) and (9) led to the formation of longer polymer chains. In the specific case in which (3) and (9) elements originate from that same chain, two connected segments (10 ★) and (11 ★) are created. Therefore, this latter reaction led to the formation of a polymeric ring, what was marked with * after element number.

**Figure 6:**
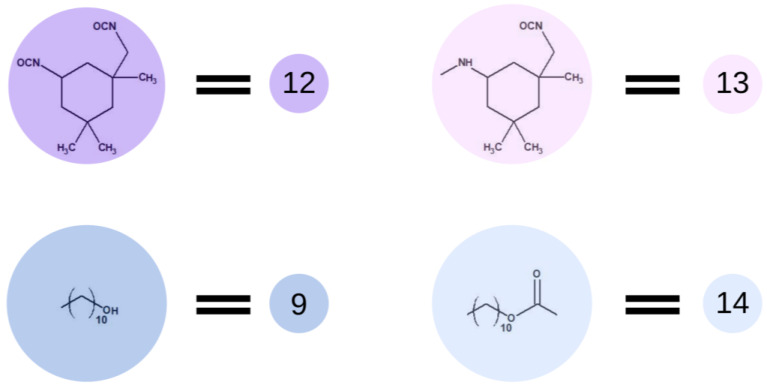
Schematic representation of the base unit participating in the prepolymer synthesis.

Then, the polyaddition between OCD chains and diisocyanate was simulated. The use of a high molar excess of diisocyanate over the OCD prevents the chain extension reaction, reducing the probability of reacting the primary aliphatic and less reactive isocyanate group of IPDI. In most literature reports [15,20], in the case of the above-mentioned materials, a three-fold molar excess of isocyanate was used. Additionally, the reactions were carried out under an inert gas atmosphere to exclude the possibility of a reaction of IPDI with moisture. The catalyst was not present, and the temperature of the reaction was around 80 °C. Operating at this temperature prevents dimerization and trimerization of the isocyanate groups as well as the formation of allophanate bonds. Therefore, for the purpose of the simulation, we decided to reduce the possibility of the formation of bonds other than those between OCD and IPDI. Following the previous terminology, in Figure 6, we presented the end-groups of OCD chains described as element (9), which changed to element (14) after the reaction. IPDI, described as element (12), is changing to element (13) while terminating the polymer chains.

**Figure 7:**
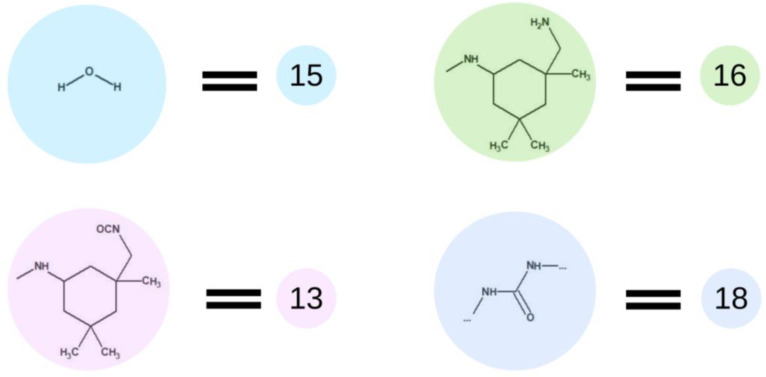
Schematic representation of the base unit participating in the curing process.

Although the curing process is particularly important when investigating final properties of the material, we decided to rely on optimized process conditions, and therefore, the curing step of all the mentioned cases was performed in that same manner. Figure 7 presents elements used in the fourth step of simulations of this process. In this step, we the water molecules described as the element (15) were added. Prepolymer interaction with water leads to hydrolysis of isocyanate groups and creation of an amino group marked as element (16). This element immediately reacts with the isocyanate group in the next molecule, i.e., with another element (15), which leads to the formation of a urea bond, represented by element (18). The urethane and urea groups form hydrogen bonds between urethane–urea, urethane–urethane and urea–urea groups. In terms of the type of materials discussed, carbonates are involved in the creation of hydrogen bonds as well. All of the discussed hydrogen bonds are leading to the creation of a physically cross-linked polymer.

## 3. Results and Discussion

### 3.1. Characterization of PCUUs

In this work, we performed computer simulations of PCUU synthesis. The detailed characterization of the structural, thermal, mechanical, and thermomechanical properties of obtained samples was also performed. Furthermore, shape-memory properties of PCUUs were investigated.

The average molar masses of soluble fractions of PCUUs were determined using GPC. The results reported in Table 1 suggest that samples were physically cross-linked, which is indicated by the large dispersity index (Đ) and high weight average molar mass (M_w_). The molar masses of final PCUUs were much higher than in the case of OCDs, which confirms the chain extension of synthesized and cured structures.

Our experiments suggest that we cannot rely on the accuracy of the GPC measurement with polystyrene-based calibration. That is why we relied on the results obtained with the use of NMR and MALDI-TOF techniques. For instance, the number average molar mass (M_n_) of E_OCD_3000, estimated based on ^1^H NMR, was equal 3000 g × mol^−1^ and was in agreement with the one obtained by means of the MALDI-TOF technique (2910 g × mol^−1^, Table S1 and Figure S29, Supplementary Materials).

In the case of samples E_PCUU5000_3, E_PCUU5000_2, and E_PCUU3000_3.5, the proposed experimental methodology led to physically cross-linked structures, while the remaining samples were soluble in chloroform. Gel fraction (G_f_) and the degree of swelling (Q_m_) were determined in this solvent (Table 2). Presented results show that a high excess of diisocyanate compound is not solely responsible for the creation of a polymeric gel. In the case of discussed PCUUs, volumetric deformation leading to gel creation seems to also be strongly related to the length of the OCD chain. E_PCUU3000_3.5 had a high capacity to absorb chloroform with Q_m_ of approximately 8400%. Samples based on E_OCD_5000 had significantly lower swelling ratios: Q_m_ = 4480% for E_PCUU5000_3, and 5840% in the case of E_PCUU5000_2. We have observed a higher content of gel fraction in the case of higher IPDI excess, which leads to a decrease in chain mobility. Therefore, the swelling ability decreased with decreasing cross-linking of the material, resulting in the general rule that higher values of Q_m_ are connected with a lower G_f_ [55]. PCUUs based on E_OCD_3000 prepared with a molar excess of IPDI below 3.5 were soluble in the chloroform, or G_f_ was insignificant enough to be undetected using the described method.

On the other hand, hard segment (HS) content in the sample is an important factor when analyzing the solubility of PCUUs; results indicate that the OCD chain length plays a significant role as well. It seems possible that these results are due to a higher amount of carbonyl groups present in the soft chain segment, which can form hydrogen bonds. Therefore, carbonyl–carbonyl interactions among hard and soft segments are possible in greater intensity along the length of the chain.

### 3.2. Mechanical Tests

To investigate the mechanical properties of PCUUs, tensile tests were performed, while the tensile strength (σ), elongation at break (ε), as well as Young’s modulus (E) were determined (Table 2, Figures S36–S42 in the Supplementary Materials). It was shown that PCUUs based on OCD with E_OCD_3000 exhibited σ > 20 MPa and ε > 800%. Similarly, PCUUs based on OCD with E_OCD_5000 have shown σ over 20 MPa and ε around 1000%. It should be mentioned that, due to the high flexibility of the samples, the phenomenon of sliding out of the grips occurred. The parameters are given for the maximum recorded values.

### 3.3. Thermal Analyses

**Figure 8:**
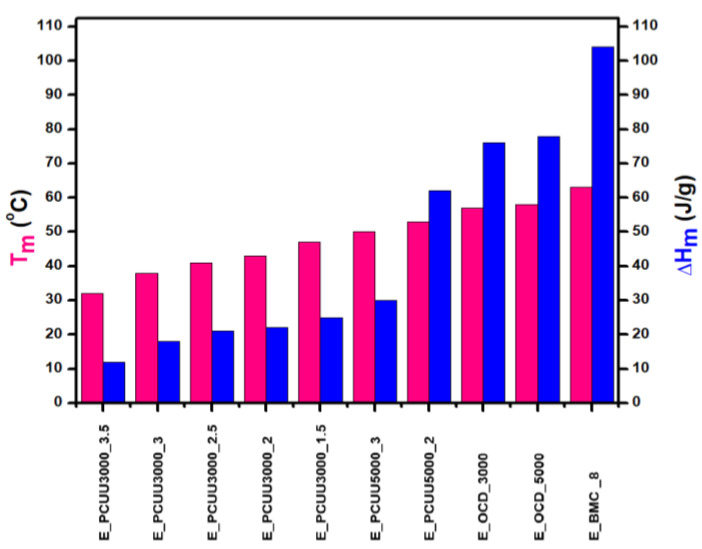
Thermal properties determined based on DSC measurements of PCUUs, OCDs, and BMC: melting points T_m_ (pink color) and melting enthalpies ΔH_m_ (blue color).

Thermal analyses of BMC, OCDs, and PCUUs were performed using DSC (Table 3, Figure 8, and Figures S43–S54, Supplementary Materials). The results have clearly shown that the crystallization temperature (T_c_) is strongly related to the content of hard segments in PCUU structures. The highest T_c_ of 19 °C was observed in the case of E_PCUU_5000_2, containing the lowest content of HS among the synthesized PCUUs. In the case of E_PCUU_5000_3, a slightly lower T_c_ of 11 °C was observed. PCUUs based on E_OCD_3000 showed T_c_ values in the range of 12 °C to 0 °C, which decreased with an increase in HS content in the structure. The T_c_ of E_BMC_8 and OCDs were much higher, at around 40 °C. Enthalpy of crystallization (ΔH_c_), in the case of PCUUs, decreased with higher HS content in the samples based on the same OCD. E_PCUU5000_3 and E_PCUU5000_2 exhibited higher ΔH_c_ values for a similar HS share in samples based on E_OCD_3000. A decrease in the ΔH_c_ value can be observed with the subsequent steps of the synthesis from 123 J × g^−1^ for E_BMC_8. After the second step of the synthesis, enthalpy declined to around 80 J × g^−1^, and then from 13 J × g^−1^ to 35 J × g^−1^ for E_PCUU3000_3.5 to E_PCUU5000_2, respectively. As the subsequent steps of the PCUU synthesis were carried out, the less crystalline phase was formed. This may be due to the disturbance of the regular structure introduced by a long chain and the content of rigid segments, which in the case of IPDI are asymmetric.

**Figure 9:**
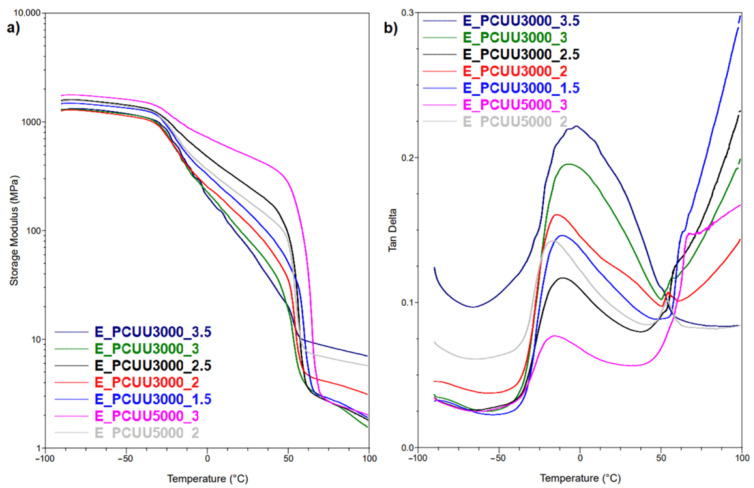
DMTA plots for (**a**) storage modulus and (**b**) Tan Delta as a function of temperature of PCUUs.

**Figure 10:**
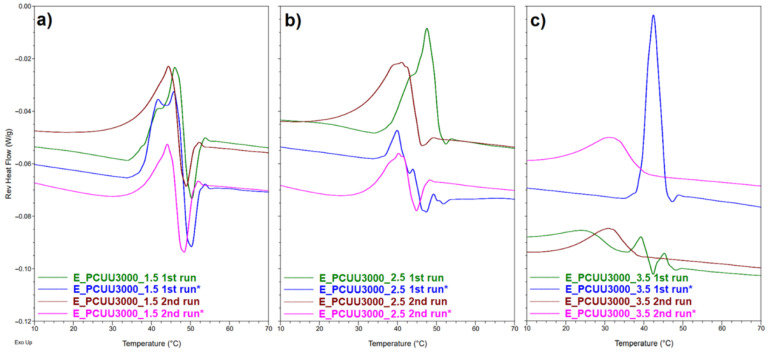
Reversible heat flow curves based on MDSC measurements of (**a**) E_PCUU3000_1.5; (**b**) E_PCUU3000_2.5; and (**c**) E_PCUU_3.5. The * symbol refers to sample elongated with 100% strain (1st run *) and the second heating run of the previously elongated sample after its return to the primary shape (2nd run *).

DSC technique provides relatively quick and easy quantitative and qualitative analysis of transitions occurring in tested materials. Although extensive research has been carried out using this technique, studies such as these do not necessarily show subtle changes, or the precision essential for the analysis of shape-memory materials. MDSC applies the sinusoidally modulated heating rates. Therefore, the possibility to determine reversible events associated with glass transition and the melting process, as well as non-reversible changes such as crystal rearrangement, annealing, cold crystallization, and relaxation during glass transition, is introduced in this method [56,57]. On the other hand, DMTA allows the determination of material properties as a function of stress, frequency, time, and/or temperature. In the presented study, we determine the glass transition temperature (T_g_) and the melting point (T_m_) of PCUUs using MDSC and DMTA (Table 4 , Table 5, Figure 9 and Figure 10, and Figures S55–S62 in the Supplementary Materials).

To investigate T_g_, MDSC and DMTA were used. These methods are more sensitive to changes occurring at the T_g_ in comparison to DSC, in which the mentioned thermal transition was not detected. A higher molar excess of the IPDI caused an increase in the T_g_ (based on Tan Delta) from −15 °C in the case of E_PCUU3000_2, to −2 °C for E_PCUU3000_3.5 (Table 4 and Figure 9). This could be explained by the effect of main chain stiffness dependence on IPDI excess. In the case of more rigid polymer structures, containing a higher isocyanate content, a higher T_g_ was recorded. A similar tendency was observed for T_g_ based on the MDSC (Table 5); however, the trend was not so clear.

DSC and MDSC (Table 3 and Figure 10) show consistently that T_m_ decreased with increasing isocyanate excess used in the synthesis. The T_m_ values of E_PCUU3000_3.5 increased from 32 °C and 42 °C in DSC and MDSC measurements, respectively, to 47 °C for E_PCUU_1.5 in the case of both techniques. Interestingly, PCUUs based on E_OCD_3000 analyzed with DMTA showed that T_m_ was maintained at around 50 °C for samples from E_PCUU3000_3.5 to E_PCUU3000_2, with an increase to 57 °C for E_PCUU3000_1.5.

### 3.4. Shape-Memory Properties

Shape-memory properties of the synthesized materials were investigated using the ε_prog_ = 100% (Table 6). Data show that there is a strong correspondence between the OCD average chain length and shape recovery and fixity ratios, which was reported previously [20]. In the case of E_PCUU5000_3 and E_PCUU5000_2, thermocycle measurements were conducted with the use of a tensile testing device due to the inability to determine the parameters that would allow the tests to be carried out using a DMTA analyzer. All samples have exhibited satisfactory R_f_ and R_r_ parameters of around 99% (Table 6 and Figures S30–S35 in the Supplementary Materials). It is important to highlight the fact that, in the case of longer polymer chains, the degree of crystallinity is also higher. This conclusion supports the fact that using longer switching segment chains leads to a greater possibility of crystallization within the switching segment domains. What is more, the R_f_ values increases and R_r_ slightly decreases with a lower content of hard segments in PCUUs based on the E_OCD_3000.

Recently we showed that higher values of ε_prog_ of PCUUs led to an increase in crystallinity, and values of T_m_ and ΔH_m_ [20]. Here, we investigated samples elongated to 100%. As can be seen from Table 5, the reported T_m_ has not changed significantly. However, for samples from E_PCUU3000_3.5 to E_PCUU3000_2, an increase in ΔH_m_ can be observed in the first cycle, which can suggest elongation-induced crystallization related to the orientation of polymer chains. On the other hand, lower ΔH_m_ values were recorded in the second run for all samples. In addition, for almost all stretched samples, smaller values of ΔH_m_ can be observed than for cast films. Most probably, this originates from the fact that deformed samples need more time after heating and melting to recrystallize under the measurement conditions. Furthermore, the majority of samples stretched by ε_prog_ = 100% had lower values of T_g_ in comparison to the initial samples but the differences were not significant. In Figure 10, reversible heat flow curves based on MDSC show that, in the second heating cycle, the thermal history of the sample is removed, thus reducing the degree of crystallinity induced by stretching the sample. It can be observed that with the increase in HS, the differences for the cast and stretched samples are more visible.

### 3.5. Results of Monte Carlo Simulations

#### 3.5.1. Synthesis of Bis(methylcarbonate)decamethylene

**Figure 11:**
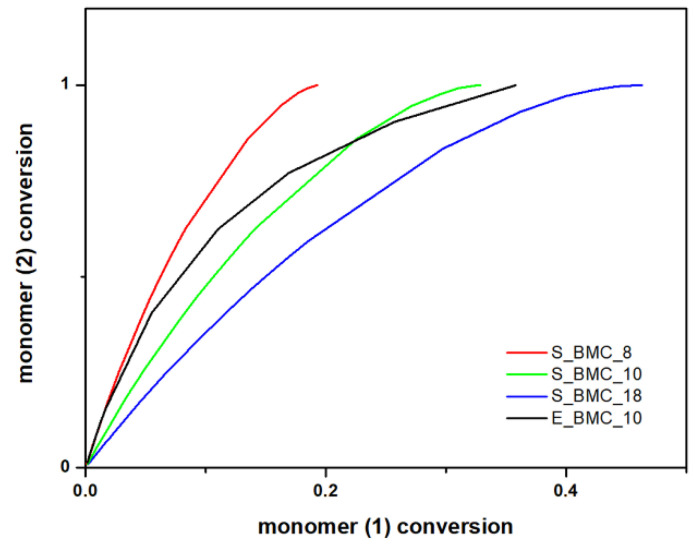
DLL simulation and experimental data of the conversion of 1,10-decanodiol versus the conversion of the DMC.

The simulation results obtained within the frame of the DLL model allow not only the prediction of the molar masses of the synthesized products, but also acquaintance with the progress of the reaction regarding the individual components. Figure 11 presents the conversion of the diol component to DMC. As one would expect, all the diol molecules and part of the second starting material reacted, as the latter was added in significant molar excess. Along with the course of the reaction and creation of longer chain structures, the curve slope, i.e., the conversion rate of element (2) in relation to element (1), decreases with the course of the reaction. The curve began to flatten near 0.35 in the case of E_BMC_10, and 0.3 for S_BMC_10, which suggests that, in the final stage of the discussed synthesis, some part of the unreacted DMC was still in the system, which is consistent with the applied synthetic route (Figure S63 in the Supplementary Materials). There is evidence that experimental studies are more accurate because of the sensitivity of the registration process, especially in the final stages of the reaction. Implemented synthesis assumes that, after the discussed step, the DMC excess is removed from the reaction mixture.

**Figure 12:**
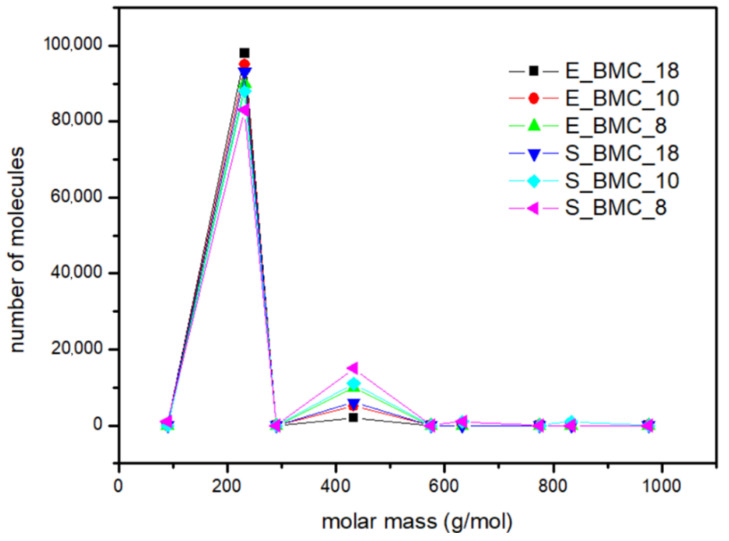
The mass distribution of the BMC: summary of experimental results and simulations for various input parameters.

The main focus of the experiments at this stage was to calculate the mass distribution of synthesized BMC. Based on the ^1^H NMR results (Figures S1–S3 in the Supplementary Materials), the fraction of dimers and longer homologues obtained in the reaction were determined. The comparison of starting materials and products obtained in the reaction and simulation presented in Table 7 and Figure 12 shows that slightly longer structures are formed in the simulation. Interestingly, the higher excess of DMC in the initial solution was observed, and the higher dimer content in the final product was observed for both the simulations and experiments. Figure 12 presents the molar mass distribution obtained in the experiments and simulations of the synthesis process. In this figure, more extended structures, in the case of experimental studies, have been presented as trimers. It reveals good agreement between both methods; the main characteristics of the curves are similar. Moreover, using the adopted simulation model enables determination of the quantitative shares of the remaining reaction products, which are often too negligible to be detected in less sensitive analytical tests. In the case of S_BMC_18, the longest created structures were octamers, whereas for S_BMC_8 it was decamers. Interestingly, the greater amount of DMC in the reaction mixture led to reduction in longer structures, whereas the dimer mass contribution for S_BMC_18 was 93%, while in the case of S_BMC_8, it decreased to 84%. This is consistent with experimental data, where E_BMC_18 had a 98% dimer mass contribution that decreased to 90% for E_BMC_8 (Figure S1, Supplementary Materials).

**Figure 13:**
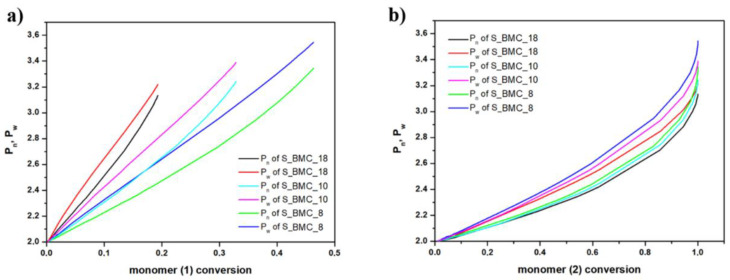
DLL simulation of (**a**) the growth of number average chain length (P_n_) and weight average (P_w_) with DMC (**b**) and 1,10-decanodiol conversions.

In Figure 13, the growth of the number average chain length (P_n_) and weight average (P_w_) are compared with conversion of elements (1) and (2). One can observe from Figure 13a, that the molar mass depends on the conversion of element (1) almost linearly. This agrees with our expectations as we have used a significant molar excess of element (1), and these elements could gradually react at almost unchanged conditions throughout this reaction stage. This conclusion is also based on the fact that, in the case of conversion of elements (2) presented in Figure 13b, the dependency of the degree of polymerization (DP) on the conversion of element (2) is approximately exponential. The rapid increase in DP above the conversion 0.8, i.e., in the last stages of the synthesis, suggests formation of longer copolymer structures (mainly trimers). As expected, P_n_ and P_w_ have similar characteristics for individual trials. Since the molar excess of monomer (1) was introduced, the biggest changes were recorded. As dilution of the monomer increases, the P_n_ and P_w_ gradually took higher values, indicating the creation of longer structures. These observations also agree with the experimental results outlined in Figure 12.

**Figure 14:**
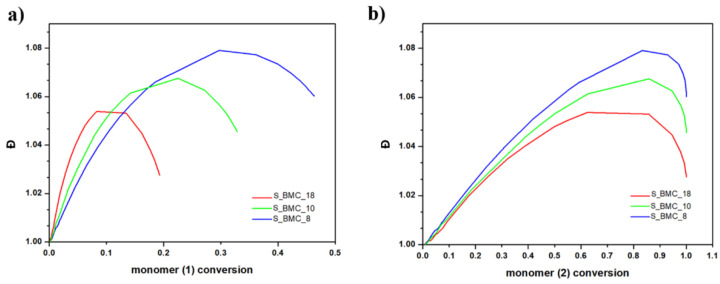
DLL simulation of dispersity index (Đ) with conversion of (**a**) (1) monomer and (**b**) (2) monomer.

For both monomers with increasing conversion, an increase in dispersity index (Đ) was observed up to 1.08 for S_BMC_8. These results led to a similar conclusion as presented above, that mostly dimers were created. It was found that Đ is substantially higher in samples with a higher monomer (1) content in the initial mixture of substrates. This also corresponds to the rapid first stage of Đ growth of conversion of element (1) in comparison to (2), since the molar excess of DMC was significant in the system (Figure 14).

#### 3.5.2. Synthesis of Oligo(decamethylene carbonate) Diols

In the second stage of synthesis, the BMC reacted with the same diol in the appropriate molar ratio, calculated based on the composition of the bis(methylcarbonate) decamethylene mixture. The process leads to the formation of linear molecules and cycles, which was taken into account in the proposed simulation model.

**Figure 15:**
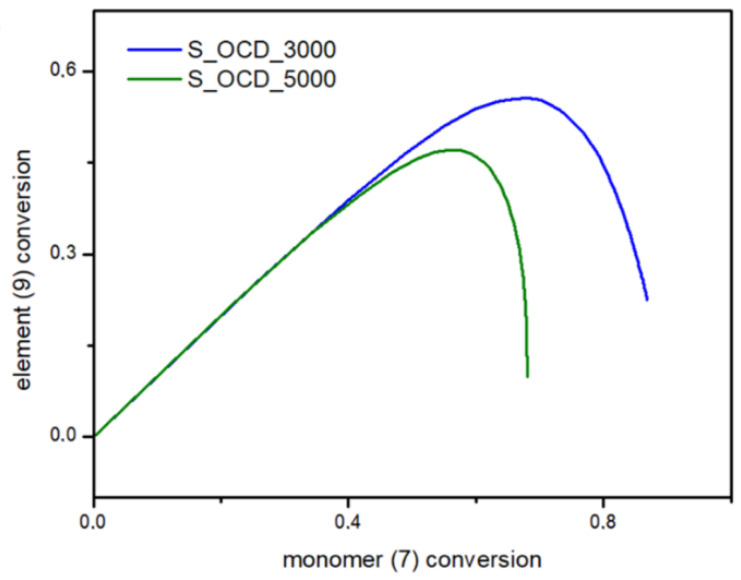
Conversion of ending groups of growing polymer chain (9) as a function of the 1,10-decanediol (7) conversion.

**Figure 16:**
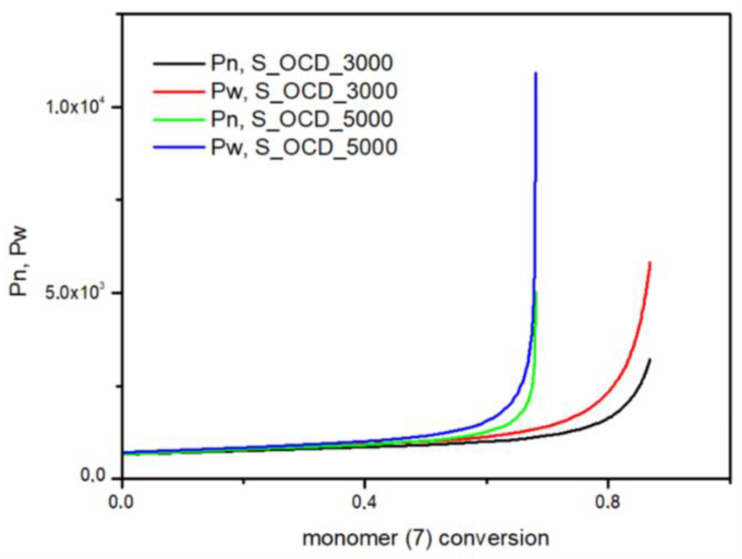
The number average molar mass (P_n_) and the weight average molar mass (P_w_) as a function of conversion of the 1,10-decanediol added in second stage of synthesis.

**Figure 17:**
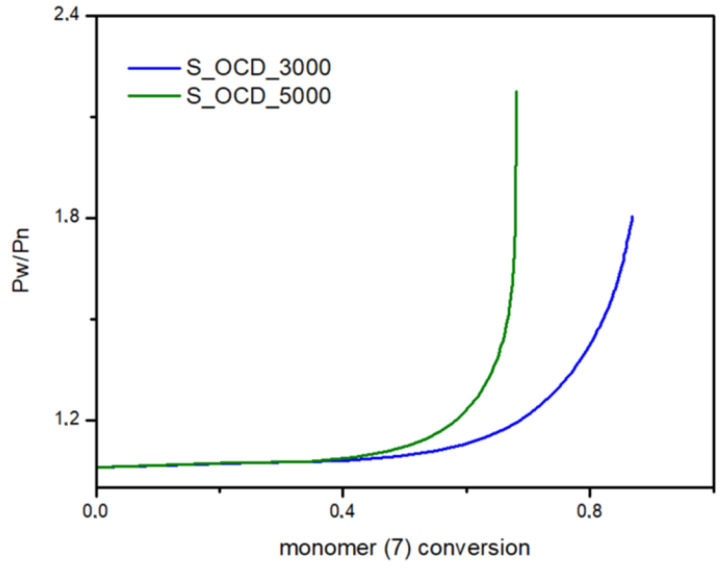
Dispersity (P_w_/P_n_) as a function of the conversion of 1,10-decanediol added in the second stage of the synthesis.

In Figure 15, the conversion of the 1,10-decanediol to one unreacted -OH group of a growing polymer chain is presented. The conversion to element (9) grows rapidly but above the conversion of the diol equal to 0.5, it starts to decline. It can be seen that S_OCD_3000 has higher conversion rates in comparison to S_OCD_5000. Figure 16 presents changes in average molar masses (P_w_ and P_n_) as a function of the 1,10-decanediol conversion. In the range of up to half of the added monomer, we notice a gradual increase in the average molar mass, and then rapid growth is observed. This behavior is expected as the growing chains start to react with each other, which leads to the formation of longer structures. A similar phenomenon was observed in the case of course of average molar masses; however, in this case P_w_ in the final phase took higher values. These results correspond with the MALDI-TOF results; it can be also seen that P_w_ is about one-third greater than the P_w_ for the final synthesis product (Table S1 in the Supplementary Materials). In Figure 17, the Đ as a function of the conversion of the monomer is presented. The experimental dispersity index was around 1.3 ± 0.1, while simulations delivered a higher Đ value, i.e., above 1.8. Based on previously mentioned assumptions, the synthesis was stopped when the average molar mass reached ca. 3000 g × mol^−1^ for E_OCD_3000 and ca. 5000 g × mol^−1^ for E_OCD_5000.

**Figure 18:**
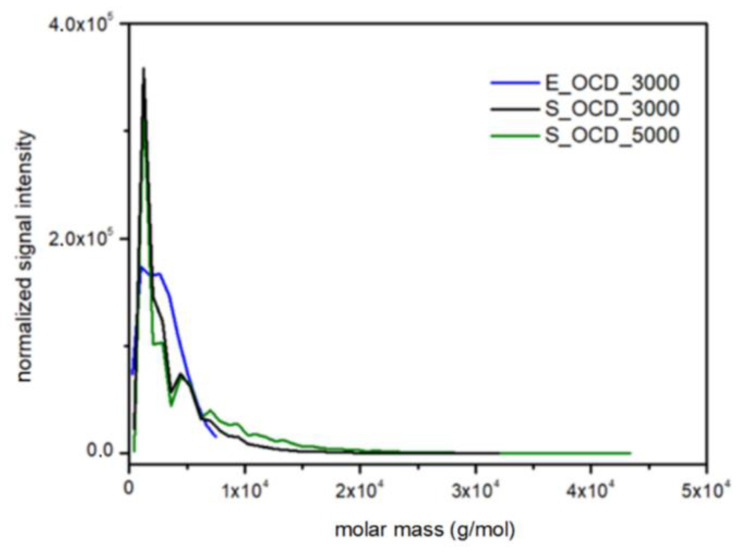
Comparison of the molar mass distributions obtained from MALDI-TOF traces for E_OCD_3000 and DLL simulations for S_OCD_3000, S_OCD_5000.

Molar mass distributions in experimental data were obtained by means of MALDI-TOF spectrometry (Table S1, Figure S29 in the Supplementary Materials). A Bruker Peptide Calibration Standard (1047.19–3149.57 Da) was used during the research, therefore, only the E_OCD_3000 sample was used in the following considerations. Figure 18 shows a normalized signal intensity as a function of the molar mass of OCDs. For DLL simulations, the molar mass was calculated using the Savitzky–Golay smoothing method in order to match experimental results. It has to be underlined that, in the latter case, chains longer than ca. 3000 g × mol^−1^ can be observed, which is not possible using the MALDI-TOF method. The scaling process influences the intensity of the highest peaks, therefore experimental results exhibit a lower normalized signal intensity in comparison to simulated results. However, the place of their occurrence and molecular mass coincides in the case of simulation and experiment.

#### 3.5.3. Urethane Prepolymers

In the next stage, the OCDs obtained in the previous step were reacted with different molar excesses of IPDI. The result of this process is the creation of prepolymer materials. As mentioned previously, molar excess of diisocyanate reduces chain extension. Therefore, in the current work, we decided that the IPDI molecule can only react with the ending groups of oligocarbonate chains. We compared the results of the proposed method with the reference in the experiment. Polymer chains with specific molecular weights from 3000 to 5000 g × mol^−1^ were formed in the previous steps. In addition, the IPDI molecules are described as element (12), where one molecule corresponds to one element. As in the previous stages, the experimental calculations were converted to match the described simulation elements.

#### 3.5.4. Curing of Urethane Prepolymers with Water Vapor

Finally, the processes of curing the PCUU prepolymers were simulated. Here, the curing of all the mentioned cases was performed in that same manner based on optimized process conditions. Therefore, we were able to evaluate the influence of the excess IPDI and average chain lengths used in the stages discussed above, as if excluding the direct influence of the curing process. Kozakiewicz et al. [58] investigated the influence of curing parameters such as temperature, humidity, and its values on the final material characterization and found the improvement of mechanical properties with an increase in curing temperature and relative air humidity. The results of this experiment indicate that the increase in the mentioned conditions led to better macromolecule organization, and therefore to the increase in the probability of covalent as well as physical cross-link formation. Gaymans et al. studied the relationship between hard segment content and tensile properties in PURs and polyamides [59,60,61]. There was a significant correlation between the increase in HS content, and yield strength and tensile strengths.

**Figure 19:**
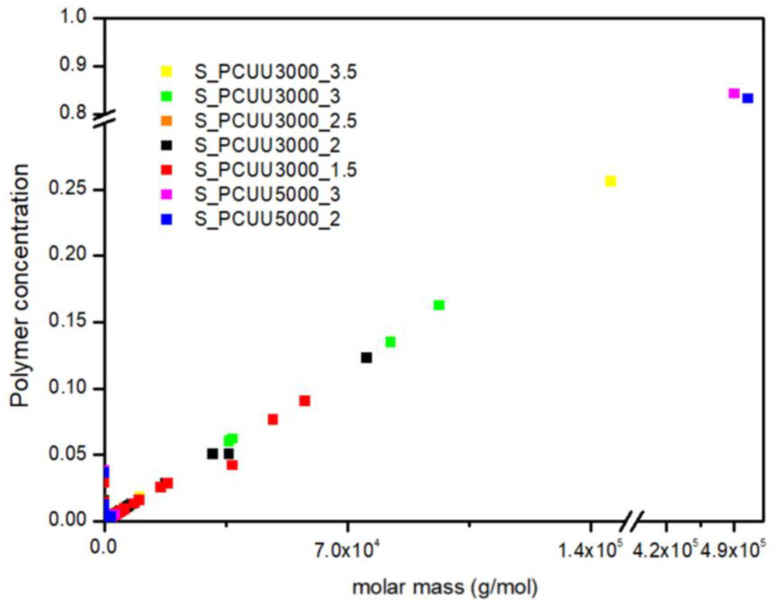
Polymer concentration profiles for PCUU samples based on OCD with molar masses of 3000 g × mol^−1^ and 5000 g × mol^−1^.

In Figure 19, we presented the polymer concentration profiles for simulated PCUU samples. This study shows that the used IPDI excess influences the synthesis process, while in the case of PCUU based on S_OCD_3000, the higher excess led to the creation of longer structures. Surprisingly, almost no differences in the molar mass of created structures were found in the case of DLL structures based on S_OCD_5000. This is due to a smaller number of end groups for the entire chain, and thus a smaller amount of the reagent used, which makes it more difficult to enter subsequent reactions. This, in turn, leads to a rapid increase in the length of formed structures.

Similarly to the experimental data, simulated PCUUs based on S_OCD_5000 were cross-linked, with a simulated G_f_ of approximately 80% (Figure 19). Samples E_PCUU5000_3 and E_PCUU5000_2 were characterized with G_f_ = 62 ± 18% and G_f_ = 51 ± 12%, respectively, which corresponds to the simulated data. The gelation leads to a reduction in the concentration of longer structures in the system, in favor of the formation of the biggest gels. However, when comparing our experimental results to those of DLL studies, it must be pointed out that, up from a molar mass range from approximately 2.5 × 10^7^ g × mol^−1^, the gel was formed. The presented results for E_PCUU3000_3.5 with G_f_ = 45 ± 4% confirmed a smaller gel fraction content in comparison to samples based on E_OCD_5000 (Table 2).

## 4. Conclusions

In the presented research we investigated the multi-step synthesis of PCUUs. Experimental data have been compared with computer simulations, supplemented by predictions of the latter for each step. For this purpose, we developed the DLL model, in which molecular transport was assumed as cooperative movements. This approach enables simulations of high-density systems, and thus, are suitable for studies of macromolecular synthesis.

In the case of BMC synthesis, the higher excess of DMC in the initial solution was used and the higher dimer content in the final product was observed, both in the experiment and in the simulation. The conversion of monomers was in satisfactory agreement between the simulation and experiment. In the discussed synthesis, some part of the unreacted methylcarbonate groups was still in the system, which is consistent with the applied synthetic route. Simulations based on the introduced DLL model enable observations of the local and global dynamics. Therefore, this kind of treatment was applied to all steps of the PCUU synthesis. By conducting the simulations, we were able to determine how the number average molar mass, weight average molar mass, and dispersity change, in relation to the conversion of monomers. Moreover, the influence of IPDI excess on the properties of the final products was investigated. The quantity of diisocyanate compound has an influence on the HS content and therefore physical cross-linking density, which has an influence on the mechanical properties of the materials and their solubility, but also on their shape-memory properties. Gelation occurs in the case of PCUUs based on longer OCDs (with M_n_ = 5000 g × mol^−1^) and for E_PCUU3000_3.5, which, in association with simulation studies, led to the determination of the polymer concentration limit, above which the system gels. What is more, the analysis of the simulated polymer concentration profiles shows that a reduction in the concentration of longer structures in the system favors the formation of the highest gel fractions. The synthesized materials exhibited good mechanical properties; in the case of PCUUs based on E_OCD_5000, σ > 20 MPa, ε≃1000%, and for PCUUs based on E_OCD_3000, σ > 20 MPa, ε > 800%. All of the synthesized samples exhibited R_f_ > 94% and R_r_ > 99%; however, the samples with a lower HS content had a tendency to flow, which meant that samples based on E_OCD_5000 had to be tested with a testing machine instead of a DMTA analyzer.

In summary, the DLL model can provide sufficient prediction and explanation of the synthesis of PCUUs based on OCDs. The proposed methodology can be directly applied to analyze the products of reactions of other polymeric syntheses carried out in a crowded environment and performed with various substrate ratios.

## Figures and Tables

**Table 1:** Molar mass and dispersity index.

Sample	M_n_	M_w_	Đ
	g × mol^−1^	g × mol^−1^	-
E_PCUU3000_3	23,550 ^a^	2.008 × 10^6 a^	85.3 ^a^
E_PCUU3000_2.5	56,290 ^a^	1.674 × 10^6 a^	29.7 ^a^
E_PCUU3000_2	43,750 ^a^	2.665 × 10^6 a^	60.9 ^a^
E_PCUU3000_1.5	64,590 ^a^	1.169 × 10^6 a^	18.1 ^a^
E_OCD_5000	7610 ^b^	19,220 ^b^	2.5 ^b^
E_OCD_3000	5950 ^b^ 2910 ^c^	13,850 ^b^ 3970 ^c^	2.3 ^b^ 1.4 ^c^

^a^ Results based on GPC measurements of the soluble fraction of the polymer. ^b^ Measurements based on GPC. ^c^ Measurement based on MALDI-TOF.

**Table 2:** Hard segment content (HS), swelling behavior, and mechanical properties of PCUU samples.

Sample	G_f_	Q_m_	HS ^a^	σ	ε	E_Young_
	%	%	wt.%	MPa	%	MPa
E_PCUU3000_3.5	45 ± 4	8430 ± 700	21 ± 1	28 ± 3	840 ± 40	67 ± 15
E_PCUU3000_3	0	-	18 ± 1	24 ± 3	990 ± 35	91 ± 7
E_PCUU3000_2.5	0	-	15 ± 1	23 ± 3	950 ± 20	108 ± 11
E_PCUU3000_2	0	-	13 ± 1	27 ± 2	960 ± 40	112 ± 9
E_PCUU3000_1.5	0	-	10 ± 1	34 ± 5	1070 ± 40	164 ± 58
E_PCUU5000_3	61 ± 18	4480 ± 670	12 ± 1	23 ± 4	1100 ± 70	172 ± 30
E_PCUU5000_2	51 ± 12	5840 ± 290	8 ± 1	22 ± 3	1020 ± 45	101 ± 22

G_f_—gel fraction content in chloroform. Q_m_—mass equilibrium swelling ratio in chloroform. ^a^—calculated based on the amounts of OCD and IPDI used in the synthesis of PCUU.

**Table 3:** Thermal properties determined based on DSC measurements of PCUUs, OCDs, and BMC.

Sample	T_c_	ΔH_c_	T_m_	ΔH_m_
	°C	J × g^−1^	°C	J × g^−1^
E_PCUU3000_3.5	−12 ± 1	13 ± 1	32 ± 1	12 ± 1
E_PCUU3000_3	−7 ± 1	19 ± 1	38 ± 1	18 ± 1
E_PCUU3000_2.5	−9 ± 1	16 ± 1	41 ± 1	21 ± 1
E_PCUU3000_2	−6 ± 1	20 ± 1	43 ± 1	22 ± 1
E_PCUU3000_1.5	0 ± 1	24 ± 1	47 ± 1	25 ± 1
E_PCUU5000_3	11 ± 1	30 ± 1	50 ± 1	30 ± 1
E_PCUU5000_2	19 ± 1	35 ± 1	53 ± 1	62 ± 1
E_OCD_3000	40 ± 1	77 ± 1	57 ± 1	76 ± 1
E_OCD_5000	39 ± 1	79 ± 1	58 ± 1	78 ± 1
E_BMC _8	42 ± 1	123 ± 1	63 ± 1	104 ± 1

**Table 4:** T_g_ and melting point based on DMTA.

Sample	T_g_ tanδ	T_g_ E′	T_g_ E″	T_m_
	°C	°C	°C	°C
E_PCUU3000_3.5	−2 ± 1	−29 ± 1	−18 ± 1	51 ± 1
E_PCUU3000_3	−7 ± 1	−33 ± 1	−22 ± 1	51 ± 1
E_PCUU3000_2.5	−11 ± 1	−31 ± 1	−21 ± 1	49 ± 1
E_PCUU3000_2	−15 ± 1	−33 ± 1	−23 ± 1	51 ± 1
E_PCUU3000_1.5	−11 ± 1	−32 ± 1	−22 ± 1	57 ± 1
E_PCUU5000_3	−16 ± 1	−31 ± 1	−23 ± 1	51 ± 1
E_PCUU5000_2	−17 ± 1	−29 ± 1	−25 ± 1	50 ± 1

T_g_ tanδ—Tg based on Tan Delta. T_g_ E′—Tg based on storage modulus. T_g_ E′′—Tg based on loss modulus.

**Table 5:** T_g_ and melting points based on MDSC for cast PCUU samples and PCUU samples elongated 100%. The * symbol refers to sample elongated with 100% strain (1st run *) and the second heating run of the previously elongated sample after its return to the primary shape (2nd run *).

Sample	T_g_		T_m_		ΔH_m_			
	1st Run	1st Run *	1st Run	1st Run *	1st Run	2nd Run	1st Run *	2nd Run *
	°C		°C		J × g^−1^			
E_PCUU3000_3.5	−27 ± 2	−31 ± 2	42 ± 2	42 ± 2	17 ± 1	23 ± 1	25 ± 1	23 ± 1
E_PCUU3000_3	−30 ± 2	−34 ± 2	43 ± 2	43 ± 2	25 ± 1	27 ± 1	40 ± 1	20 ± 1
E_PCUU3000_2.5	−30 ± 2	−34 ± 2	46 ± 2	44 ± 2	44 ± 1	40 ± 1	45 ± 1	34 ± 1
E_PCUU3000_2	−37 ± 2	−35 ± 2	44 ± 2	43 ± 2	40 ± 1	35 ± 1	48 ± 1	27 ± 1
E_PCUU3000_1.5	−32 ± 2	−34 ± 2	47 ± 2	47 ± 2	46 ± 1	36 ± 1	37 ± 1	31 ± 1
E_PCUU5000_3	−33 ± 2	−36 ± 2	55 ± 2	53 ± 2	70 ± 1	41 ± 1	60 ± 1	28 ± 1

**Table 6:** Shape-memory properties of PCUU samples.

Sample	R_r_	R_f_
	%	%
E_PCUU3000_3.5	99.6 ± 0.2	94.5 ± 0.1
E_PCUU3000_3	99.6 ± 0.1	95.8 ± 0.1
E_PCUU3000_2.5	99.4 ± 0.4	98.8 ± 0.1
E_PCUU3000_2	99.0 ± 0.6	98.2 ± 0.5
E_PCUU3000_1.5	nd	nd
E_PCUU5000_3	99.3 ± 0.0 *	99.3 ± 0.6 *
E_PCUU5000_2	98.5 ± 0.7 *	98.6 ± 0.3 *

nd—no data. Rr—Shape recovery ratio. Rf—Shape fixity ratio. *—Measurements conducted with a tensile testing machine.

**Table 7:** Molar ratios of substrates and products in the first stage of the synthesis.

Sample	Molar Ratio DMC:Diol	Mass Contribution Dimers: Longer Homologues
E_BMC_18	18:1	98:2
E_BMC_10	10:1	95:5
E_BMC_8	8:1	90:10
S_BMC_18	18:1	93:7
S_BMC_10	10:1	88:12
S_BMC_8	8:1	84:16

## Data Availability

Not applicable.

## Supplementary Materials

The following supporting information can be downloaded at: https://www.mdpi.com/article/10.3390/ijms23137064/s1.

## References

[1] Polyurethane Elastomer Market by End-User and Geography—Forecast and Analysis 2022–2026. www.technavio.com.

[2] Akindoyo J.O., Beg M.D.H., Ghazali S., Islam M.R., Jeyaratnam N., Yuvaraj A.R. (2016). Polyurethane Types, Synthesis and Applications—A Review. RSC Adv..

[3] Ge C., Lian D., Cui S., Gao J., Lu J. (2019). Highly Selective CO_2_ Capture on Waste Polyurethane Foam-Based Activated Carbon. Processes.

[4] Gavgani J.N., Adelnia H., Gudarzi M.M. (2014). Intumescent Flame Retardant Polyurethane/Reduced Graphene Oxide Composites with Improved Mechanical, Thermal, and Barrier Properties. J. Mater. Sci..

[5] Kucinska-Lipka J., Gubanska I., Janik H., Sienkiewicz M. (2015). Fabrication of Polyurethane and Polyurethane Based Composite Fibres by the Electrospinning Technique for Soft Tissue Engineering of Cardiovascular System. Mater. Sci. Eng. C.

[6] Bil M., Kijeńska-Gawrońska E., Głodkowska-Mrówka E., Manda-Handzlik A., Mrówka P. (2020). Design and in Vitro Evaluation of Electrospun Shape Memory Polyurethanes for Self-Fitting Tissue Engineering Grafts and Drug Delivery Systems. Mater. Sci. Eng. C.

[7] Janik H., Marzec M. (2015). A Review: Fabrication of Porous Polyurethane Scaffolds. Mater. Sci. Eng. C.

[8] Davis F.J., Mitchell G.R. (2008). Polyurethane Based Materials with Applications in Medical Devices. Bio-Materials and Prototyping Applications in Medicine.

[9] George Drobny J. (2014). 9—Thermoplastic Polyurethane Elastomers. Handbook of Thermoplastic Elastomers.

[10] Mazurek-Budzyńska M., Behl M., Razzaq M.Y., Nöchel U., Rokicki G., Lendlein A. (2019). Hydrolytic Stability of Aliphatic Poly(Carbonate-Urea-Urethane)s: Influence of Hydrocarbon Chain Length in Soft Segment. Polym. Degrad. Stab..

[11] Christenson E.M., Anderson J.M., Hiltner A. (2004). Oxidative Mechanisms of Poly(Carbonate Urethane) and Poly(Ether Urethane) Biodegradation: In Vivo and In Vitro Correlations. J. Biomed. Mater. Res. Part A.

[12] Srivastava R., Srinivas D., Ratnasamy P. (2006). Syntheses of Polycarbonate and Polyurethane Precursors Utilizing CO_2_ over Highly Efficient, Solid as-Synthesized MCM-41 Catalyst. Tetrahedron Lett..

[13] Ubaghs L., Fricke N., Keul H., Höcker H. (2004). Polyurethanes with Pendant Hydroxyl Groups: Synthesis and Characterization. Macromol. Rapid Commun..

[14] Tomczyk K.M., Parzuchowski P.G., Rokicki G. (2010). Oligocarbonate Diols from Ethylene Carbonate—Optimization of the Synthesis Process. J. Appl. Polym. Sci..

[15] Tomczyk K.M., Parzuchowski P.G., Kozakiewicz J., Przybylski J., Rokicki G. (2010). Synthesis of Oligocarbonate Diols from a “Green Monomer”—Dimethyl Carbonate—As Soft Segments for Poly(Urethane-Urea) Elastomers. Polimery.

[16] Khan I., Smith N., Jones E., Finch D.S., Cameron R.E. (2005). Analysis and Evaluation of a Biomedical Polycarbonate Urethane Tested in an in Vitro Study and an Ovine Arthroplasty Model. Part I: Materials Selection and Evaluation. Biomaterials.

[17] Chen W., Meng F., Cheng R., Deng C., Feijen J., Zhong Z. (2014). Advanced Drug and Gene Delivery Systems Based on Functional Biodegradable Polycarbonates and Copolymers. J. Control. Release.

[18] Elsner J.J., Mezape Y., Hakshur K., Shemesh M., Linder-Ganz E., Shterling A., Eliaz N. (2010). Wear Rate Evaluation of a Novel Polycarbonate-Urethane Cushion Form Bearing for Artificial Hip Joints. Acta Biomater..

[19] Mazurek-Budzyńska M., Razzaq M.Y., Rokicki G., Behl M., Lendlein A. (2017). High-Strain Shape-Memory Properties of Poly(Carbonate-Urea-Urethane)s Based on Aliphatic Oligocarbonates and L-Lysine Diisocyanate. Soft Mater. Biomater..

[20] Mazurek-Budzyńska M., Yasar M., Tomczyk K., Rokicki G., Behl M., Lendlein A. (2016). Poly(Carbonate-Urea-Urethane) Networks Exhibiting High-Strain Shape-Memory Effect ^†^. Polym. Adv. Technol..

[21] Mazurek-Budzyńska M., Behl M., Neumann R., Lendlein A. (2022). 4D-Actuators by 3D-Printing Combined with Water-Based Curing. Mater. Today Commun..

[22] Balko J., Fernández-D’Arlas B., Pöselt E., Dabbous R., Müller A.J., Thurn-Albrecht T. (2017). Clarifying the Origin of Multiple Melting of Segmented Thermoplastic Polyurethanes by Fast Scanning Calorimetry. Macromolecules.

[23] Fernández-D’Arlas B., Balko J., Baumann R.P., Pöselt E., Dabbous R., Eling B., Thurn-Albrecht T., Müller A.J. (2016). Tailoring the Morphology and Melting Points of Segmented Thermoplastic Polyurethanes by Self-Nucleation. Macromolecules.

[24] Fernández-D’Arlas B., Baumann R.P., Pöselt E., Müller A.J. (2017). Influence of Composition on the Isothermal Crystallisation of Segmented Thermoplastic Polyurethanes. CrystEngComm.

[25] Asensio M., Costa V., Nohales A., Bianchi O., Gómez C.M. (2019). Tunable Structure and Properties of Segmented Thermoplastic Polyurethanes as a Function Offlexible Segment. Polymers.

[26] Yilgor I., Yilgor E., Guler I.G., Ward T.C., Wilkes G.L. (2006). FTIR Investigation of the Influence of Diisocyanate Symmetry on the Morphology Development in Model Segmented Polyurethanes. Polymer.

[27] Castro J.M., López-Serrano F., Camargo R.E., Macosko C.W., Tirrell M. (1981). Onset of Phase Separation in Segmented Urethane Polymerization. J. Appl. Polym. Sci..

[28] Yilgör I., Yilgör E., Wilkes G.L. (2015). Critical Parameters in Designing Segmented Polyurethanes and Their Effect on Morphology and Properties: A Comprehensive Review. Polymer.

[29] Sun X.D., Sung C.S.P. (1996). Cure Characterization in Polyurethane and Model Urethane Reactions by an Intrinsic Fluorescence Technique. Macromolecules.

[30] Lee K.J., Eichinger B.E. (1990). Computer Simulation of the Structure and Elasticity of Polyurethane Networks: 1. Polyoxypropylene Tetrols and Hexamethylene Diisocyanate. Polymer.

[31] Lee K.J., Eichinger B.E. (1990). Computer Simulation of the Structure and Elasticity of Polyurethane Networks: 2. Polyoxypropylene Triols and 4,4′-Diphenylmethane Diisocyanate. Polymer.

[32] Lempesis N., In’T Veld P.J., Rutledge G.C. (2017). Atomistic Simulation of a Thermoplastic Polyurethane and Micromechanical Modeling. Macromolecules.

[33] Yildirim E., Yurtsever M. (2014). The Role of Diisocyanate and Soft Segment on the Intersegmental Interactions in Urethane and Urea Based Segmented Copolymers: A DFT Study. Comput. Theor. Chem..

[34] Tobushi H., Okumura K., Hayashi S., Ito N. (2001). Thermomechanical Constitutive Model of Shape Memory Polymers. Mech. Mater..

[35] Park S., Moon J., Kim B., Cho M. (2021). Multi-Scale Coarse-Grained Molecular Dynamics Simulation to Investigate the Thermo-Mechanical Behavior of Shape-Memory Polyurethane Copolymers. Polymer.

[36] van Duin A.C.T., Dasgupta S., Lorant F. (2001). ReaxFF: A Reactive Force Field for Hydrocarbons. J. Phys. Chem..

[37] Yong X., Kuksenok O., Balazs A.C. (2015). Modeling Free Radical Polymerization Using Dissipative Particle Dynamics. Polymer.

[38] Gavrilov A.A., Chertovich A.V. (2017). Copolymerization of Partly Incompatible Monomers: An Insight from Computer Simulations. Macromolecules.

[39] D’Hooge D.R., Van Steenberge P.H.M., Reyniers M.F., Marin G.B. (2016). The Strength of Multi-Scale Modeling to Unveil the Complexity of Radical Polymerization. Prog. Polym. Sci..

[40] Wang L., Broadbelt L.J. (2011). Tracking Explicit Chain Sequence in Kinetic Monte Carlo Simulations. Macromol. Theory Simul..

[41] Turgman-Cohen S., Genzer J. (2011). Simultaneous Bulk- and Surface-Initiated Controlled Radical Polymerization from Planar Substrates. J. Am. Chem. Soc..

[42] Genzer J. (2006). In Silico Polymerization: Computer Simulation of Controlled Radical Polymerization in Bulk and on Flat Surfaces. Macromolecules.

[43] Marien Y.W., Van Steenberge P.H.M., D’Hooge D.R., Marin G.B. (2019). Particle by Particle Kinetic Monte Carlo Tracking of Reaction and Mass Transfer Events in Miniemulsion Free Radical Polymerization. Macromolecules.

[44] Drache M., Drache G. (2012). Simulating Controlled Radical Polymerizations with McPolymer—A Monte Carlo Approach. Polymers.

[45] Polanowski P., Pakula T. (2002). Studies of Polymer Conformation and Dynamics in Two Dimensions Using Simulations Based on the Dynamic Lattice Liquid (DLL) Model. J. Chem. Phys..

[46] Polanowski P., Jeszka J.K., Sikorski A. (2014). Dynamic Properties of Linear and Cyclic Chains in Two Dimensions. Computer Simulation Studies. Macromolecules.

[47] Polanowski P., Jeszka J.K., Matyjaszewski K. (2014). Synthesis of Star Polymers by “Core-First” One-Pot Method via ATRP: Monte Carlo Simulations. Polymer.

[48] Polanowski P., Jeszka J.K., Matyjaszewski K. (2013). Star Polymer Synthesis and Gelation in ATRP Copolymerization: Monte Carlo Simulations. Polymer.

[49] Polanowski P., Hałagan K., Pietrasik J., Jeszka J.K., Matyjaszewski K. (2017). Growth of Polymer Brushes by “Grafting from” via ATRP—Monte Carlo Simulations. Polymer.

[50] Hałagan K., Banaszak M., Jung J., Polanowski P., Sikorski A. (2021). Dynamics of Opposing Polymer Brushes: A Computer Simulation Study. Polymers.

[51] Halagan K., Banaszak M., Jung J., Polanowski P., Sikorski A. (2021). Polymerization and Structure of Opposing Polymer Brushes Studied by Computer Simulations. Polymers.

[52] Bain E.D., Turgman-Cohen S., Genzer J. (2013). Progress in Computer Simulation of Bulk, Confined, and Surface-Initiated Polymerizations. Macromol. Theory Simul..

[53] Tobita H., Yanase F. (2007). Monte Carlo Simulation of Controlled/Living Radical Polymerization in Emulsified Systems. Macromol. Theory Simul..

[54] Kratz K., Madbouly S.A., Wagermaier W., Lendlein A. (2011). Temperature-Memory Polymer Networks with Crystallizable Controlling Units. Adv. Mater..

[55] Çaykara T., Turan E. (2006). Effect of the Amount and Type of the Crosslinker on the Swelling Behavior of Temperature-Sensitive Poly(N-Tert-Butylacrylamide-Co-Acrylamide) Hydrogels. Colloid Polym. Sci..

[56] Shieh Y.-T., Liu G.-L. (2007). Effects of Carbon Nanotubes on Crystallization and MeltingBehavior of Poly(L-Lactide) via DSC and TMDSC Studies. J. Polym. Sci. Part B Polym. Phys..

[57] Yan B., Gu S., Zhang Y. (2013). Polylactide-Based Thermoplastic Shape Memory Polymer Nanocomposites. Eur. Polym. J..

[58] Kozakiewicz J., Rokicki G., Przybylski J., Sylwestrzak K., Parzuchowski P.G., Tomczyk K.M. (2011). Studies on the Effect of Curing Conditions on the Curing Rate and Mechanical Properties of Moisture-Cured Poly(Urethane-Urea) Elastomers Containing Oligocarbonate Segments. Polimery.

[59] Arun A., Baack K.K.J., Gaymans R. (2010). Polyurethane Tri-Block Copolymers—Synthesis, Mechanical, Elastic, and Rheological Properties. Polym. Eng. Sci..

[60] Gaymans R.J. (2011). Segmented Copolymers with Monodisperse Crystallizable Hard Segments: Novel Semi-Crystalline Materials. Prog. Polym. Sci..

[61] Husken D., Gaymans A.R.J. (2009). The Tensile Properties of Poly(Ethylene Oxide)-Based Segmented Block Copolymers in the Dry and Wet State. J. Mater. Sci..

