# Supplementary material for: Synthesis of Shape-Memory Polyurethanes: Combined Experimental and Simulation Studies
Source: Int J Mol Sci. 2022 Jun 25;23(13):7064. doi: 10.3390/ijms23137064 (PMC9266580; doi:10.3390/ijms23137064)
Supplement: Supplementary file 1 [file ijms-23-07064-s001.zip › ijms-1779689-supplementary.pdf]

SUPPLEMENTARY MATERIALS

Synthesis of shape-memory polyurethanes: combined experimental and simulation studies

Karolina Rolińska[*a,b], Magdalena Mazurek-Budzyńska[a], Paweł G. Parzuchowski[a], Dominik Wołosz[a], Maria Balk[c], Krzysztof Gorący[d], Mirosława El Fray[d], Piotr Polanowski[e], Andrzej Sikorski[b]

[a] Faculty of Chemistry, Warsaw University of Technology, ul. Noakowskiego 3, 00-664 Warsaw, Poland;

[b] Faculty of Chemistry, University of Warsaw, ul. Pasteura 1, 02-093 Warsaw, Poland;

[c] Institute of Active Polymers, Helmholtz-Zentrum Hereon, Kantstraße 55, Teltow 14513, Germany;

[d] Faculty of Chemical Technology and Engineering, West Pomeranian University of Technology, Szczecin, Piastów Avenue 42, 71-065 Szczecin, Poland;

[e] Faculty of Chemistry, Technical University of Lodz, Zeromskiego 116, 90-924 Lodz, Poland;

* *Corresponding author.*

E-mail address: karolina.rolinska.dokt@pw.edu.pl

FTIR and NMR spectra of synthesized products

E_BMC_8

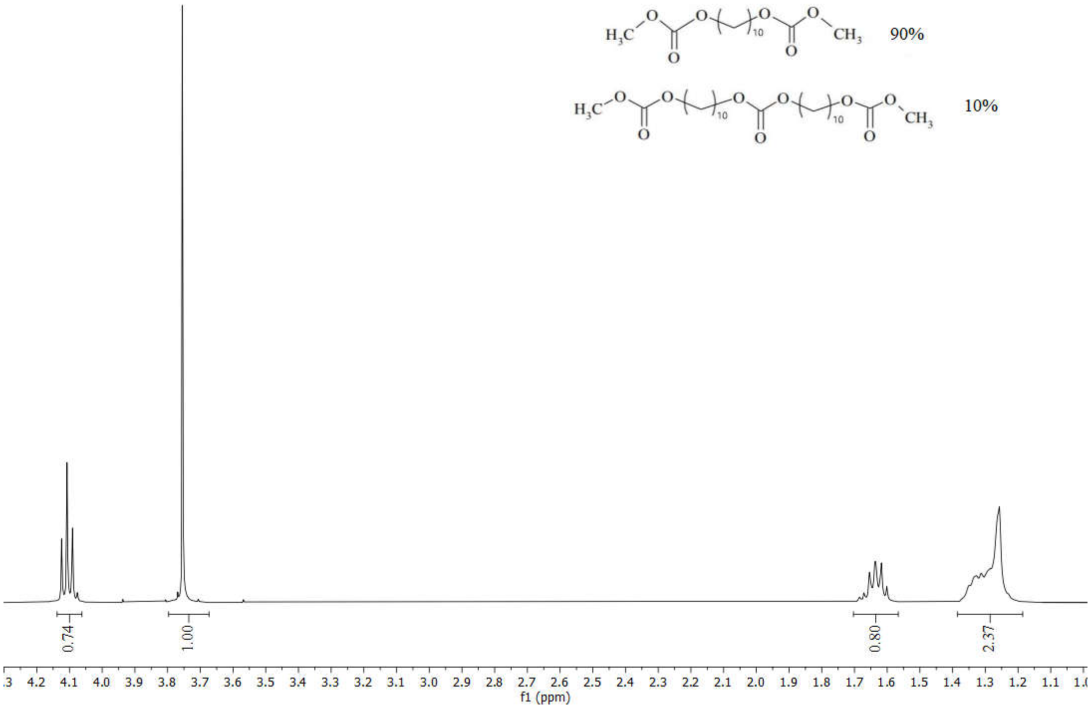

Figure S1 ^{1}H NMR spectrum of the E_BMC_8.

E_BMC_10

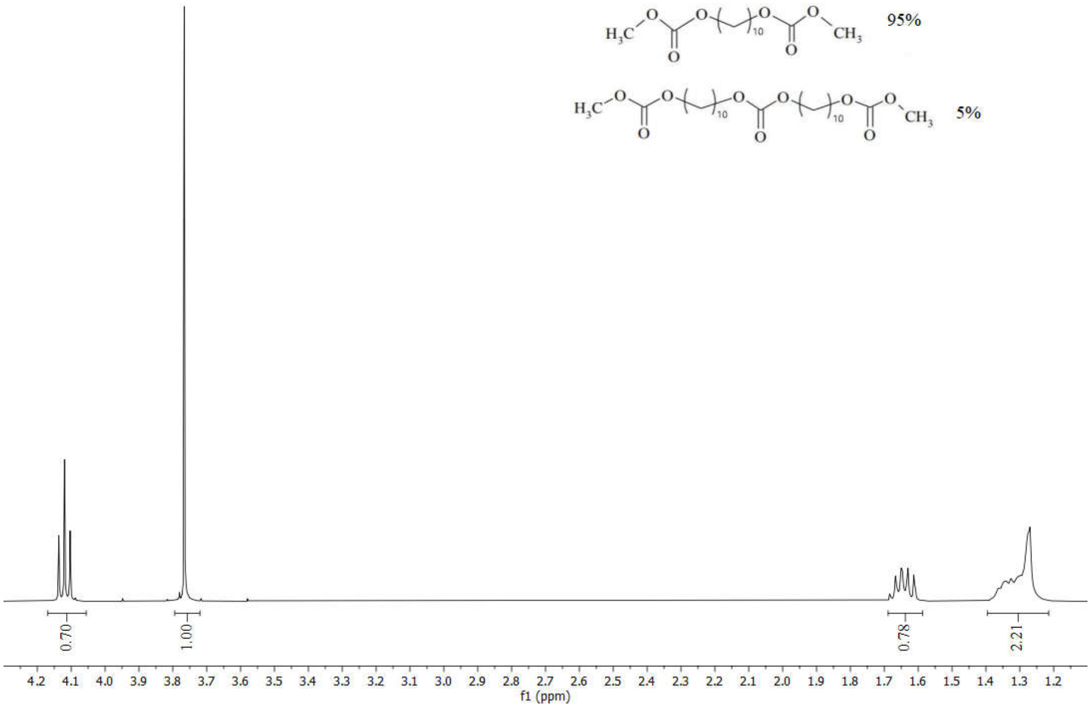

Figure S2 ^{1}H NMR spectrum of the E_BMC_10.

E_BMC_18

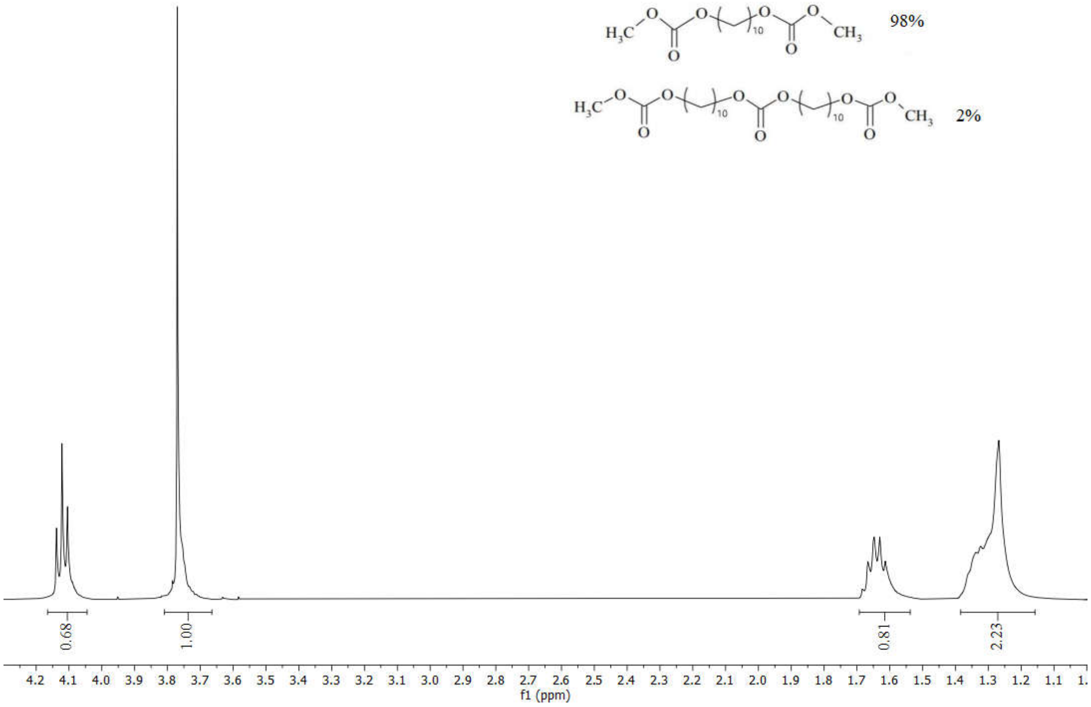

Figure S3 ^{1}H NMR spectrum of the E_BMC_18.

E_BMC_8

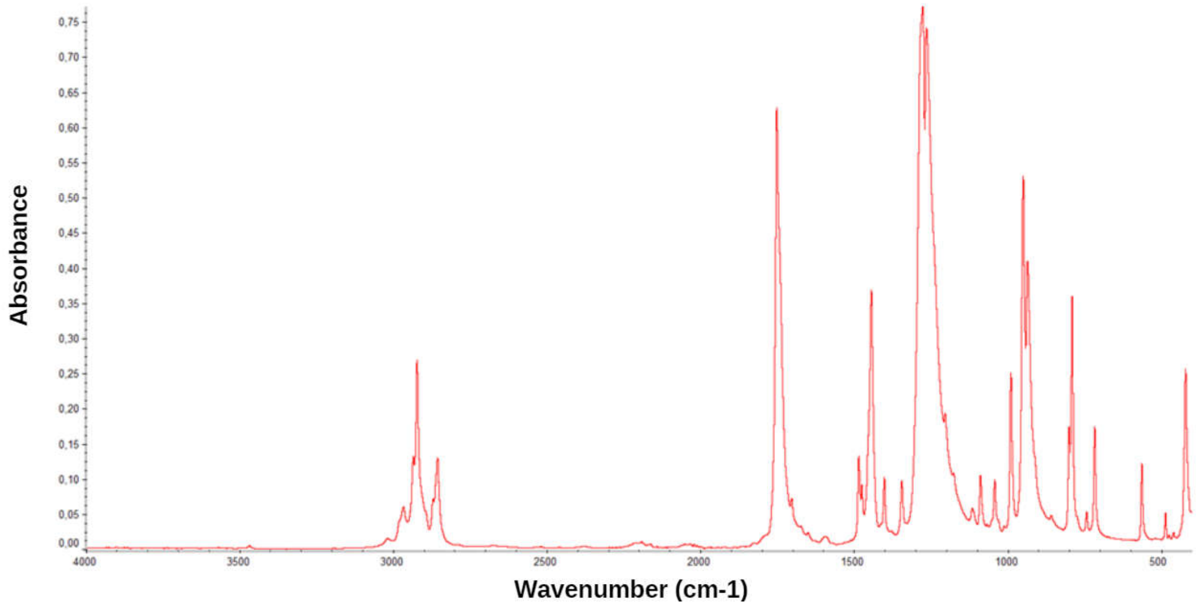

Figure S4 FTIR spectra of E_BMC_8.

E_BMC_10

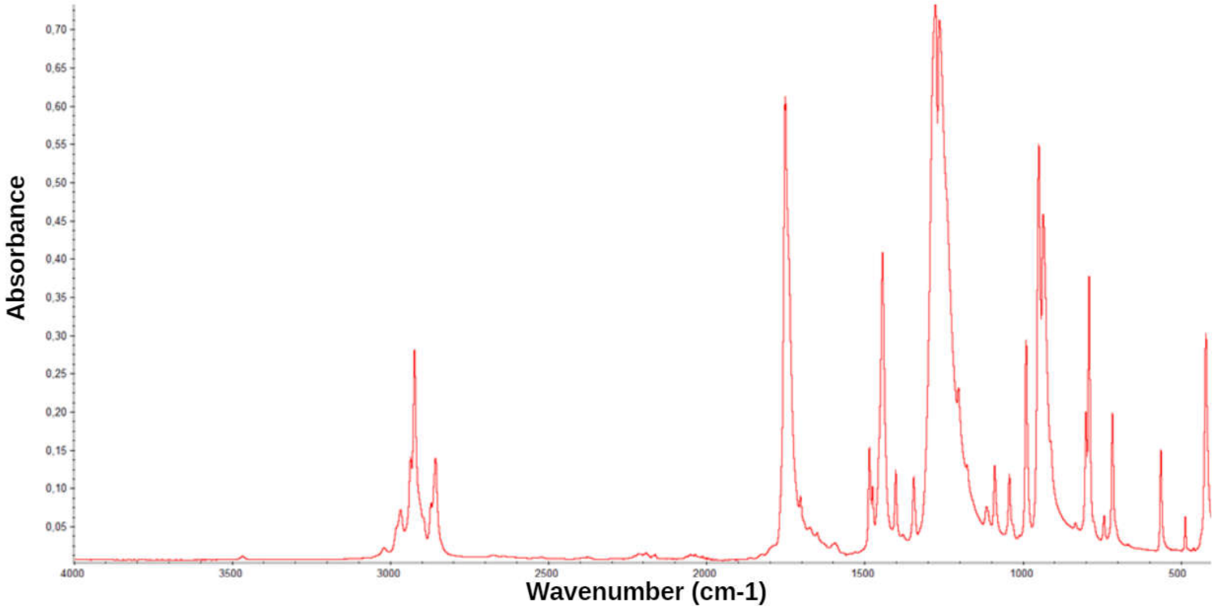

Figure S5 FTIR spectra of E_BMC_10.

E_BMC_18

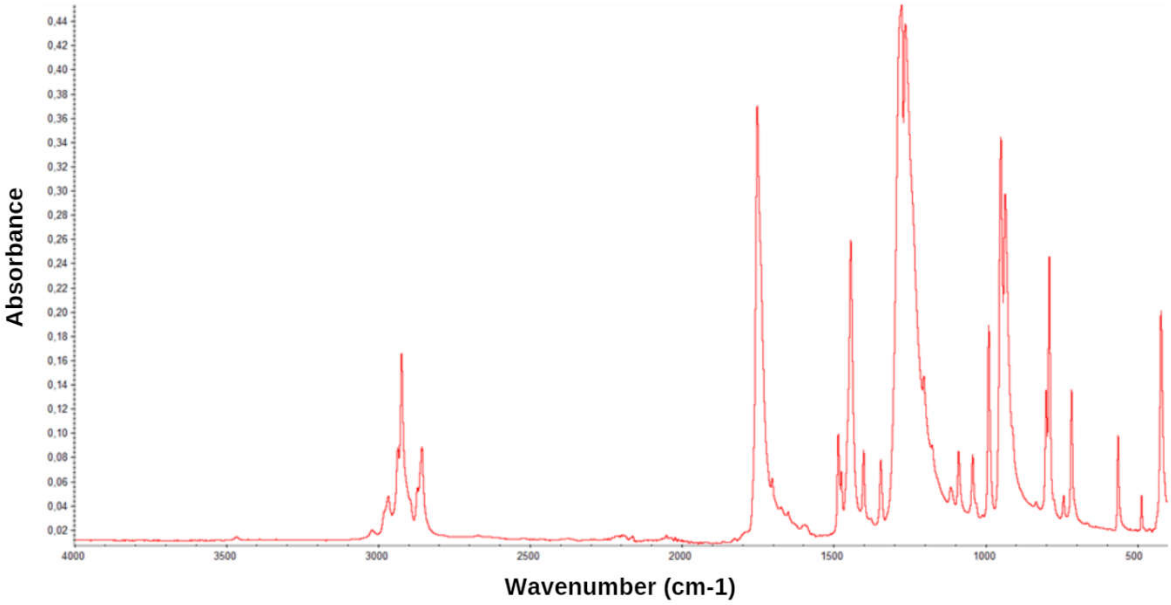

Figure S6 FTIR spectra of E_BMC_18.

E_OCD_3000

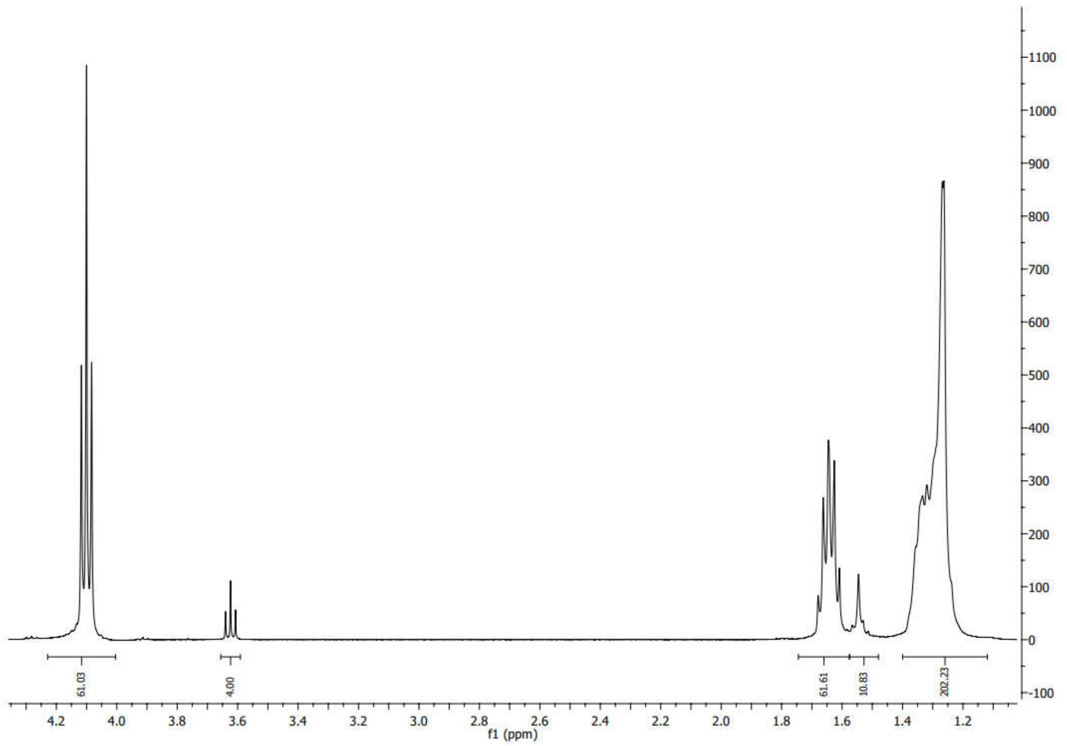

Figure S7 ^{1}H NMR spectrum of the E_OCD_3000.

E_OCD_3000

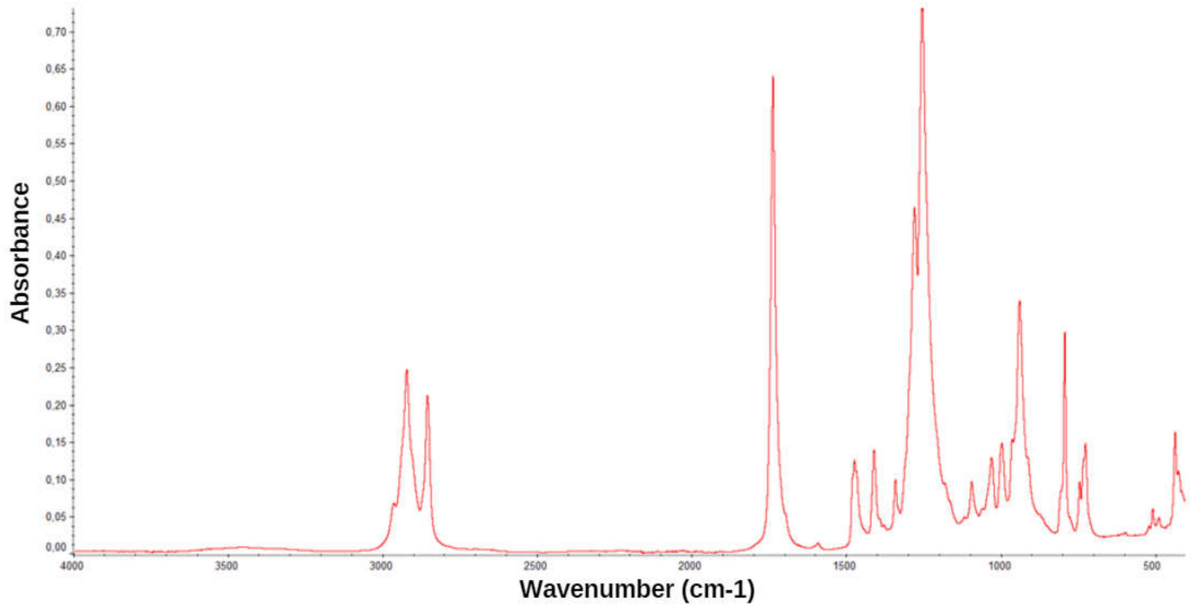

Figure S8 FTIR spectra of E_OCD_3000 oligocarbonate.

E_OCD_5000

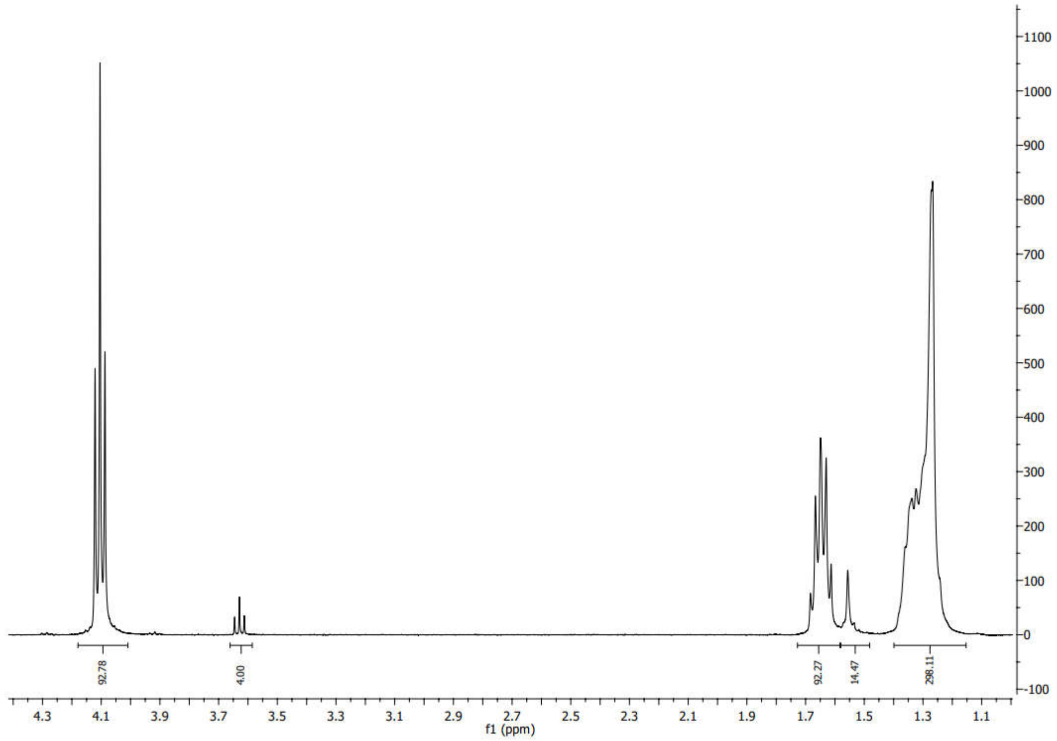

Figure S9 ^{1}H NMR spectrum of the E_OCD_5000.

E_OCD_5000

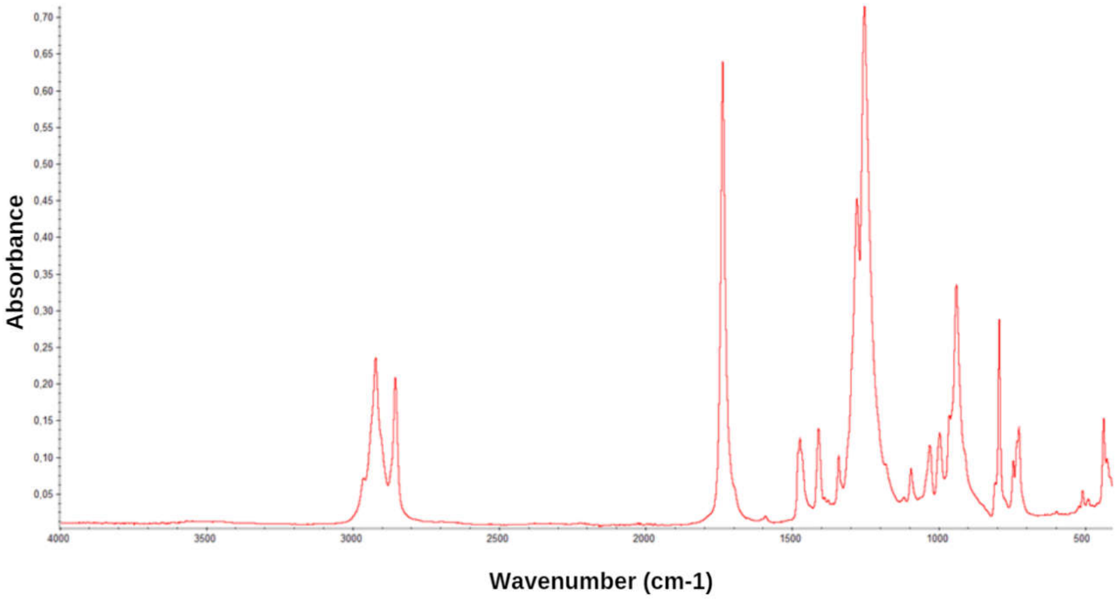

Figure S10 FTIR spectra of E_OCD_5000 oligocarbonate.

E_PCUU3000_3.5 prepolymer

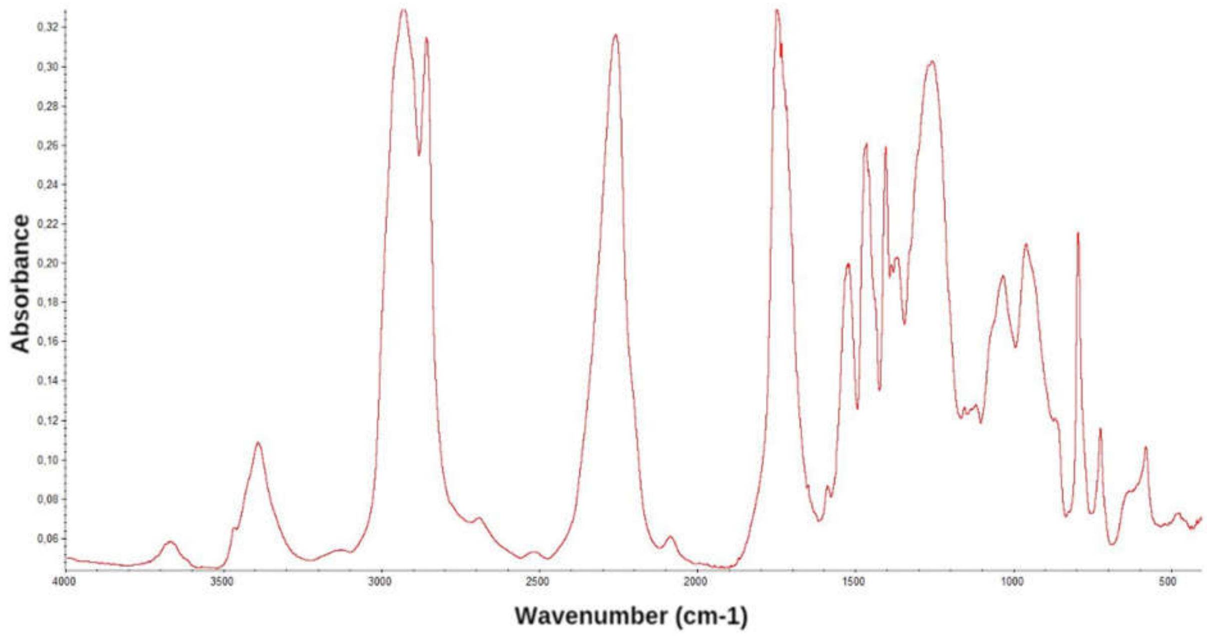

Figure S11 FTIR spectra of PCUU3000_3.5 poly(carbonate-urea-urethane) prepolymer.

E_PCUU3000_3 prepolymer

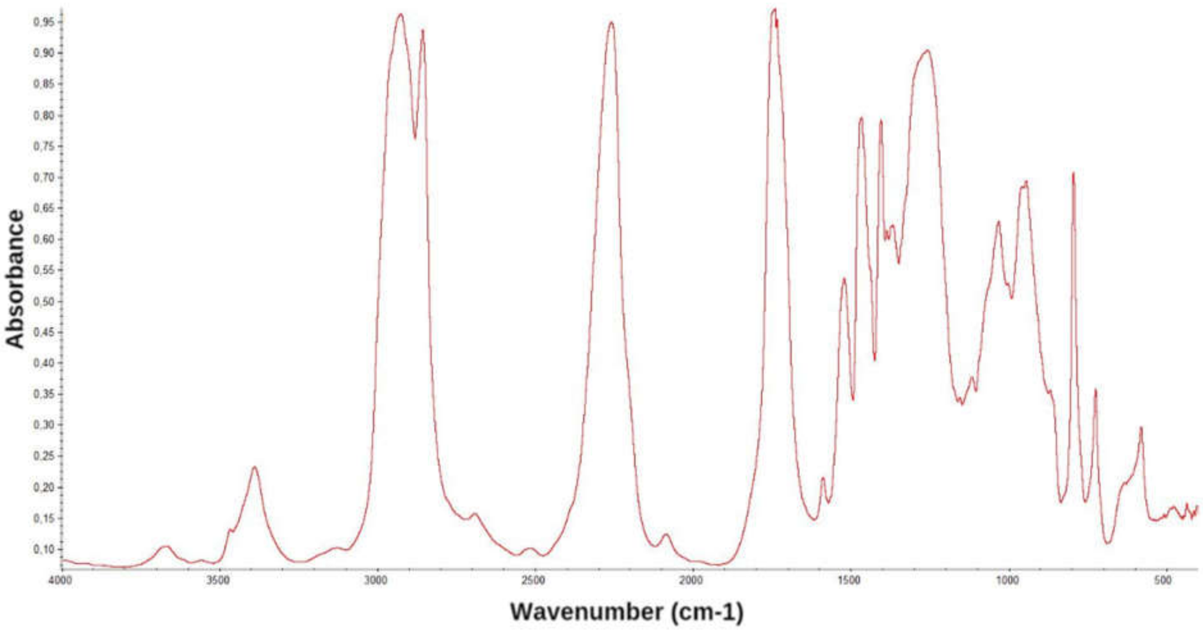

Figure 12 FTIR spectra of PCUU3000_3 poly(carbonate-urea-urethane) prepolymer.

E_PCUU3000_2.5 prepolymer

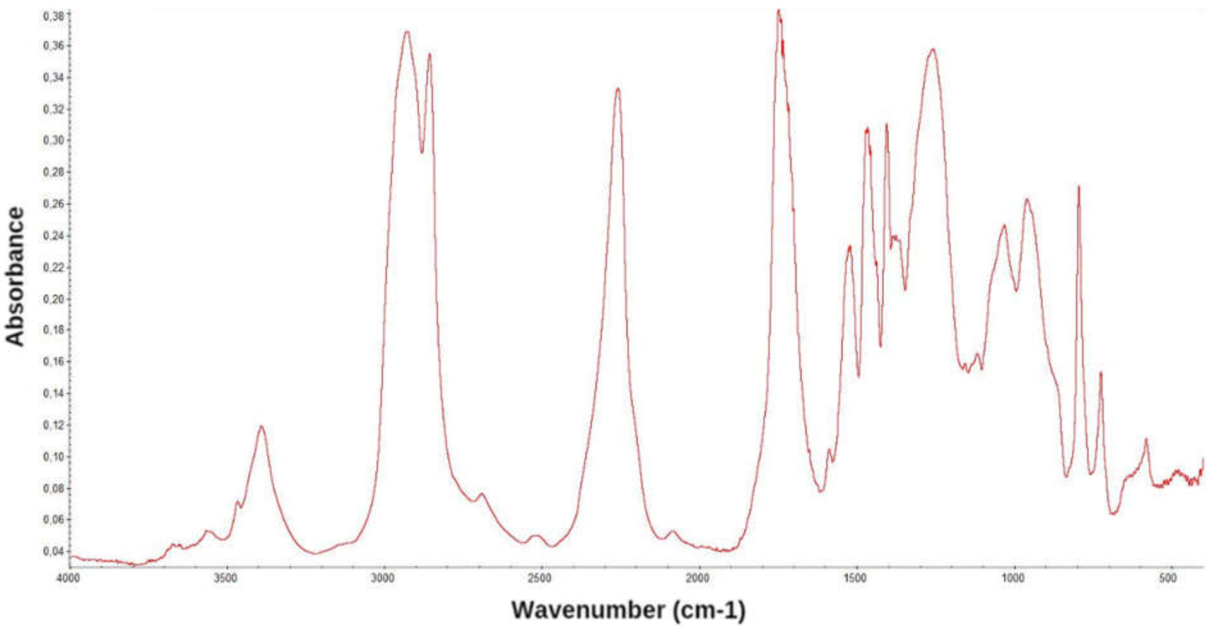

Figure S13 FTIR spectra of PCUU3000_2.5 poly(carbonate-urea-urethane) prepolymer.

E_PCUU3000_2 prepolymer

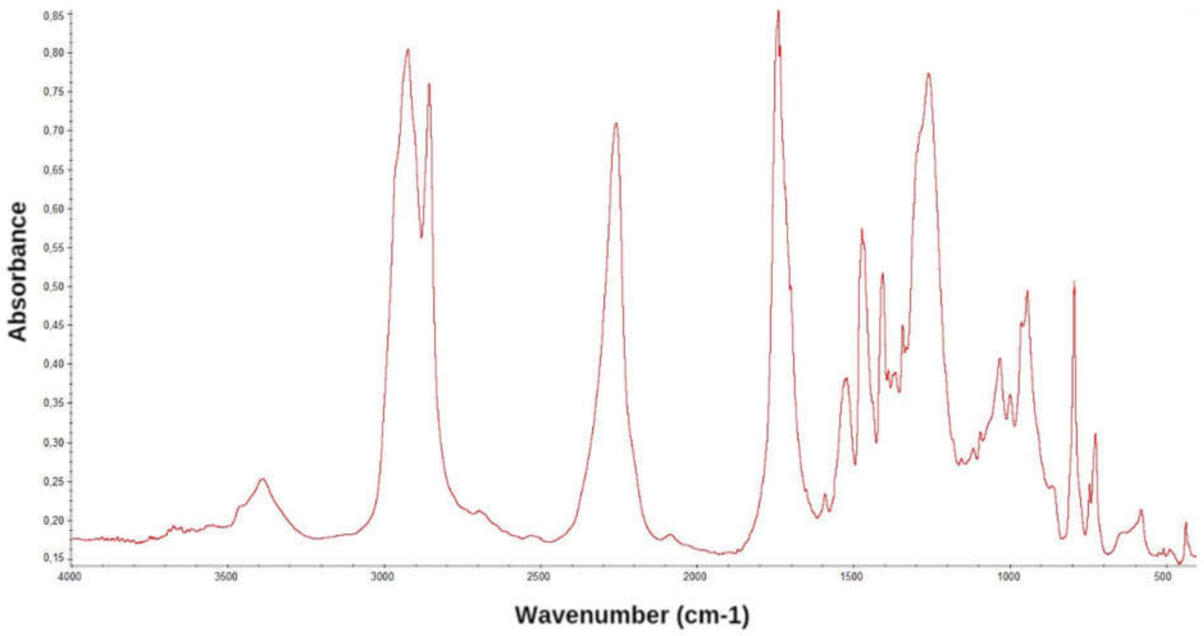

Figure S14 FTIR spectra of PCUU3000_2 poly(carbonate-urea-urethane) prepolymer.

E_PCUU3000_1.5 prepolymer

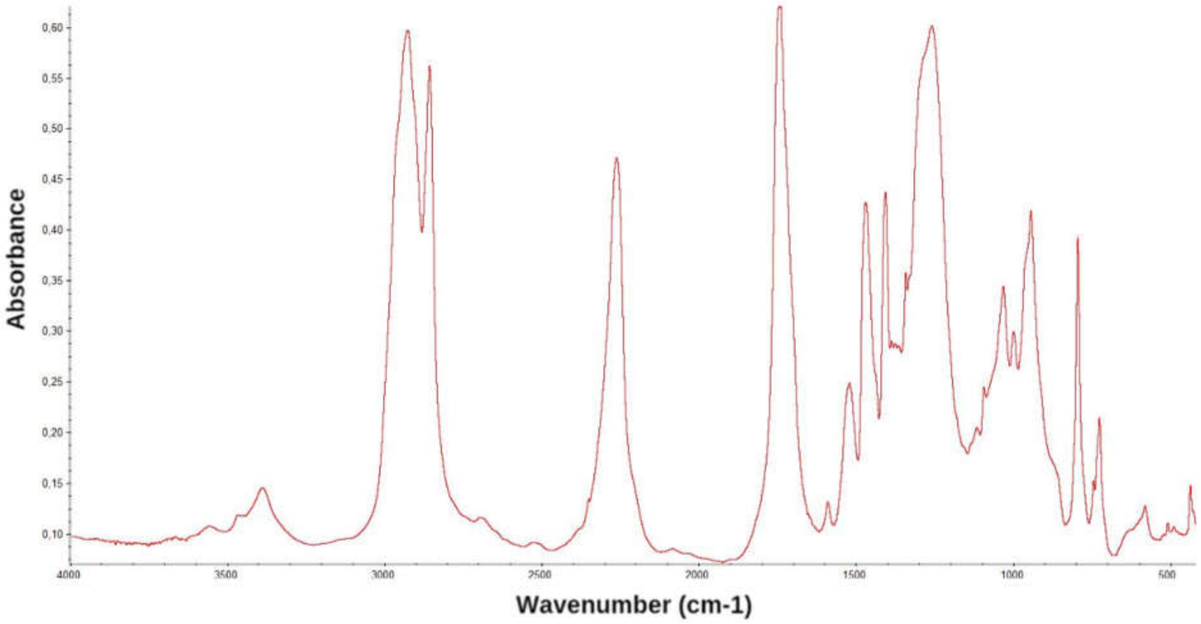

Figure S15 FTIR spectra of PCUU3000_1.5 poly(carbonate-urea-urethane) prepolymer.

E_PCUU5000_3 prepolymer

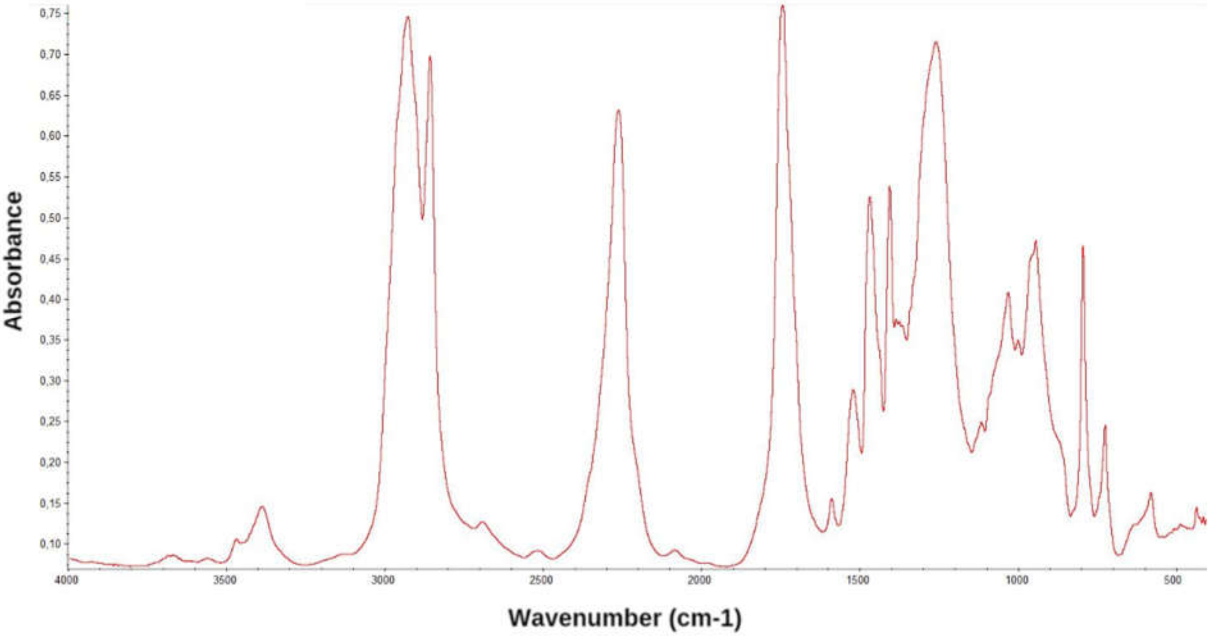

Figure S16 FTIR spectra of PCUU5000_3 poly(carbonate-urea-urethane) prepolymer.

E_PCUU5000_2 prepolymer

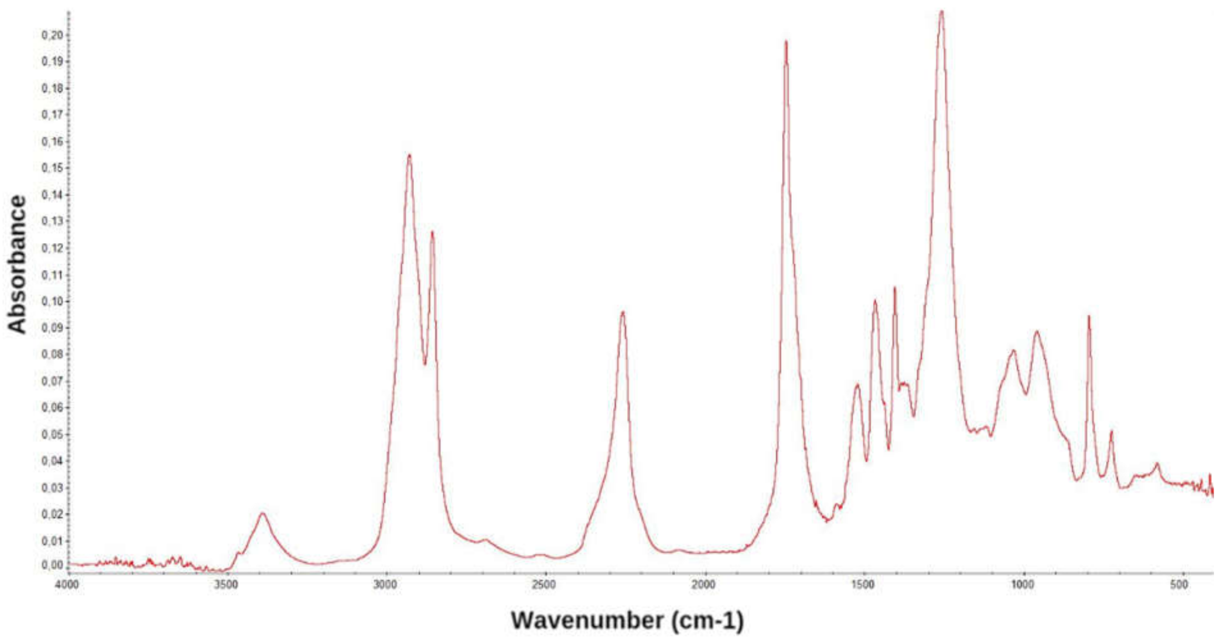

Figure S17 FTIR spectra of PCUU5000_2 poly(carbonate-urea-urethane) prepolymer.

E_PCUU3000_3

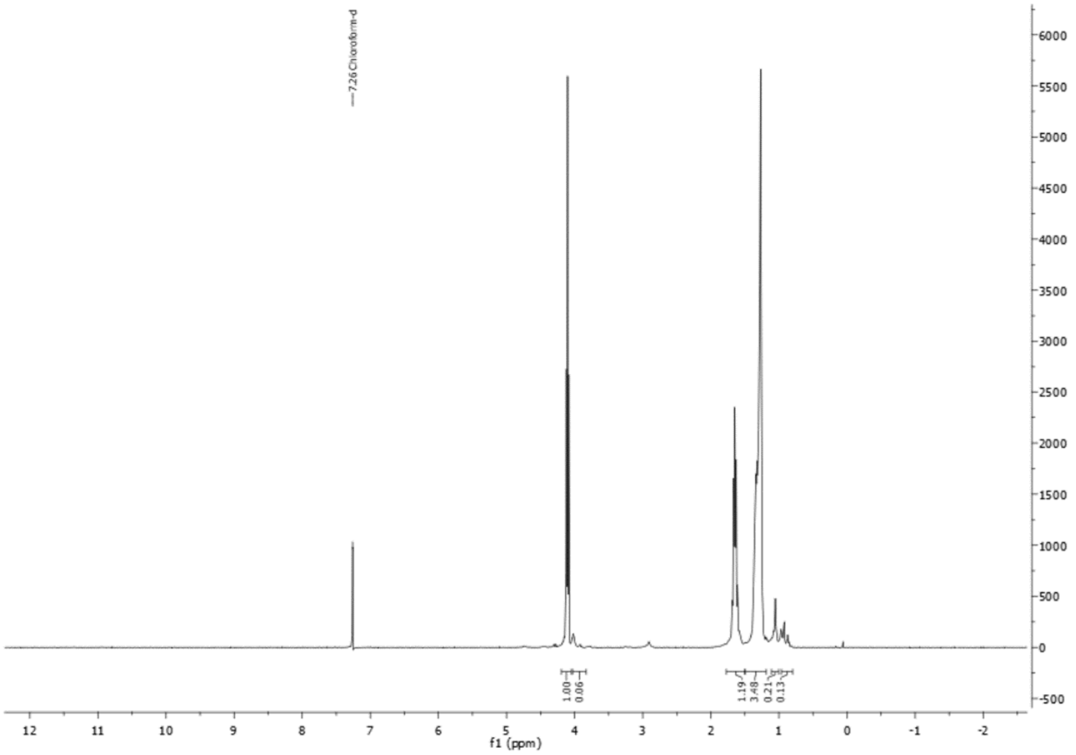

Figure S18 ^{1}H NMR spectrum of the E_PCUU3000_3.

E_PCUU3000_2.5

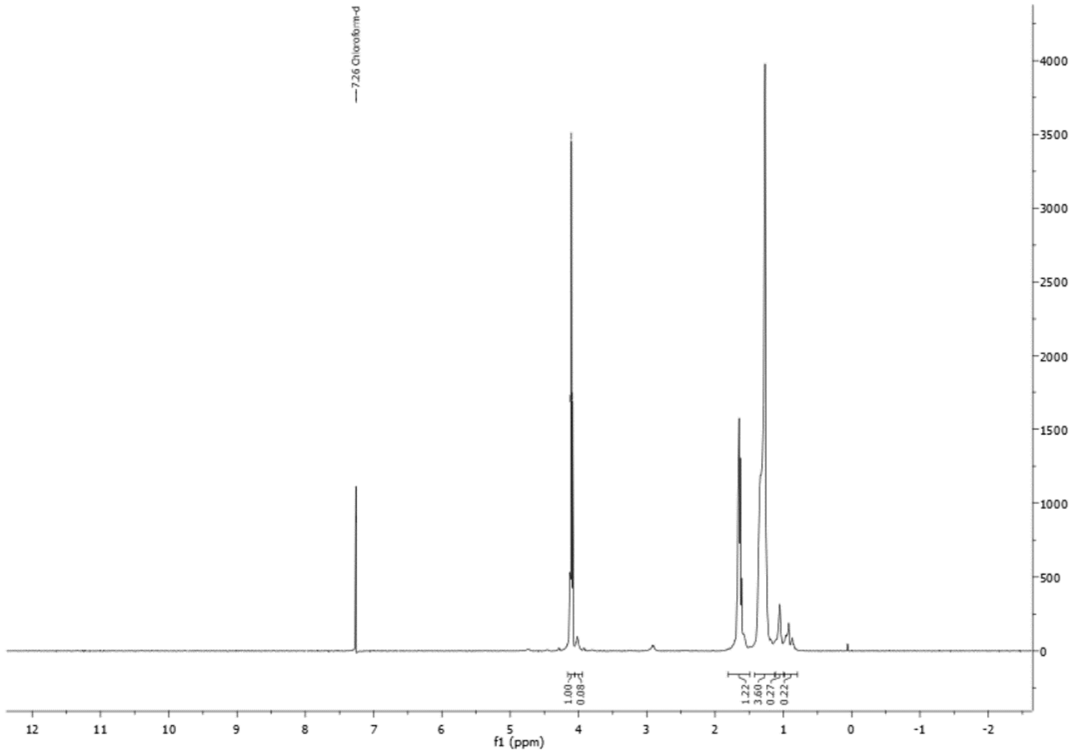

Figure S19 ^{1}H NMR spectrum of the E_PCUU3000_2.5.

E_PCUU3000_2

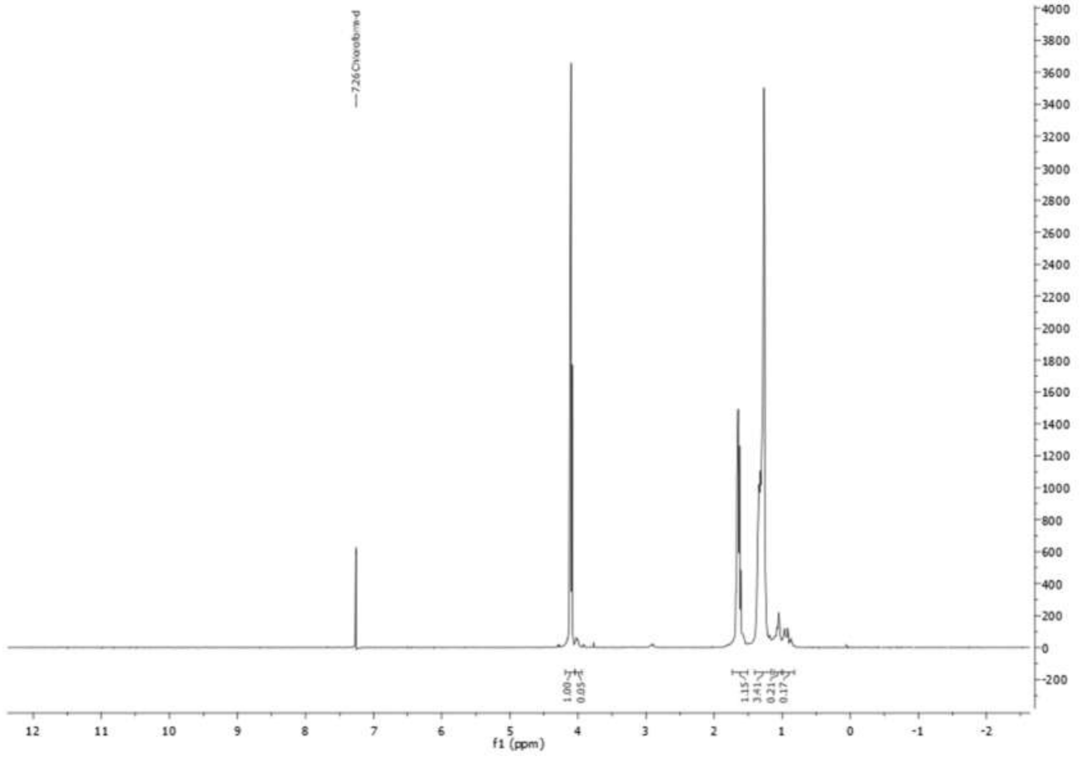

Figure S20 ^{1}H NMR spectrum of the E_PCUU3000_2.

E_PCUU3000_1.5

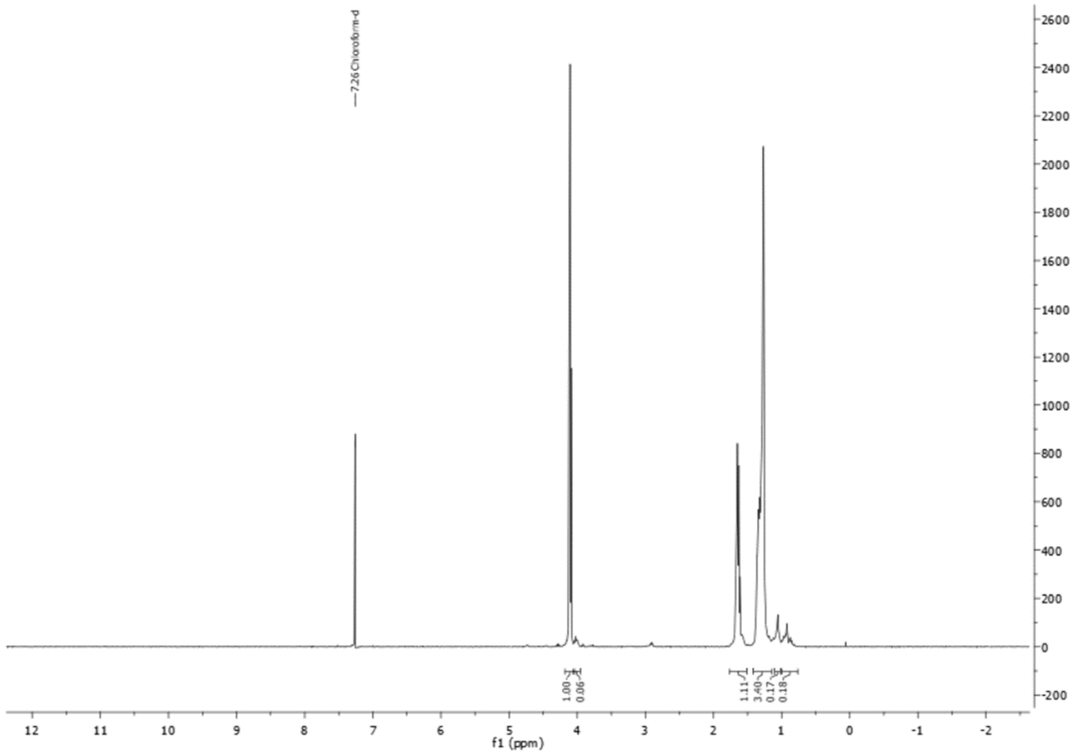

Figure S21 ^{1}H NMR spectrum of the E_PCUU3000_1.5.

E_PCUU3000_3.5

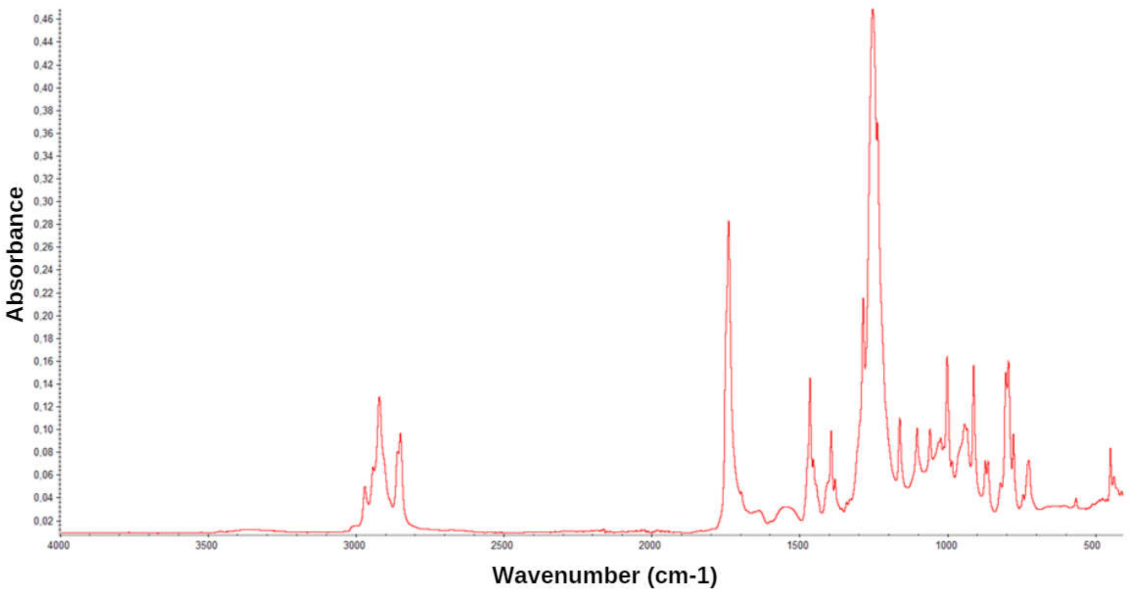

Figure S22 FTIR (ATR) spectra of PCUU3000_3.5 poly(carbonate-urea-urethane).

E_PCUU3000_3

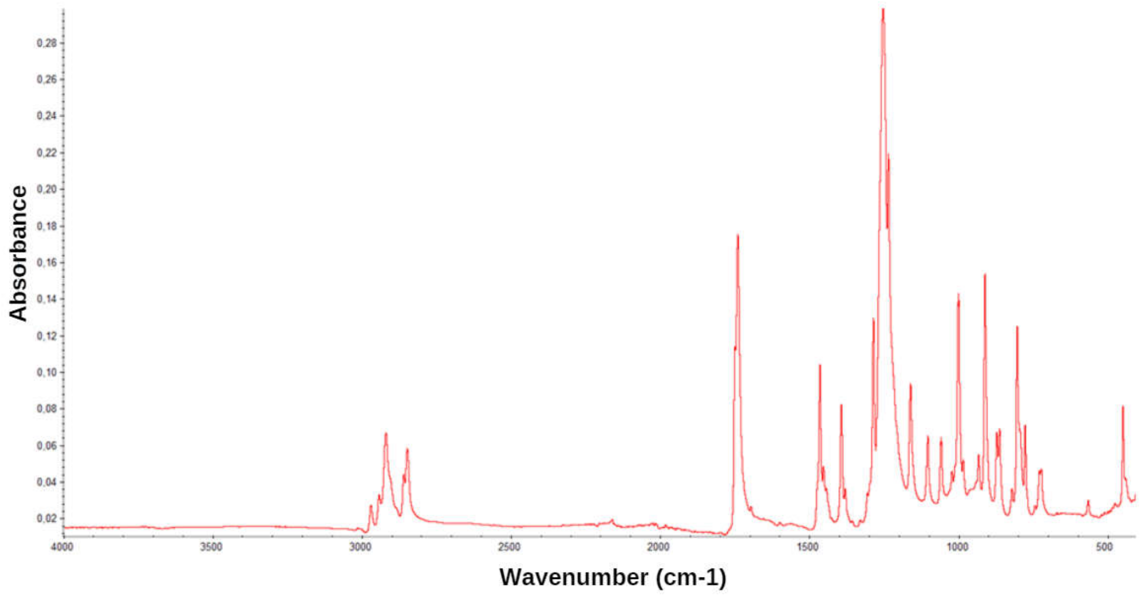

Figure S23 FTIR (ATR) spectra of PCUU3000_3 poly(carbonate-urea-urethane).

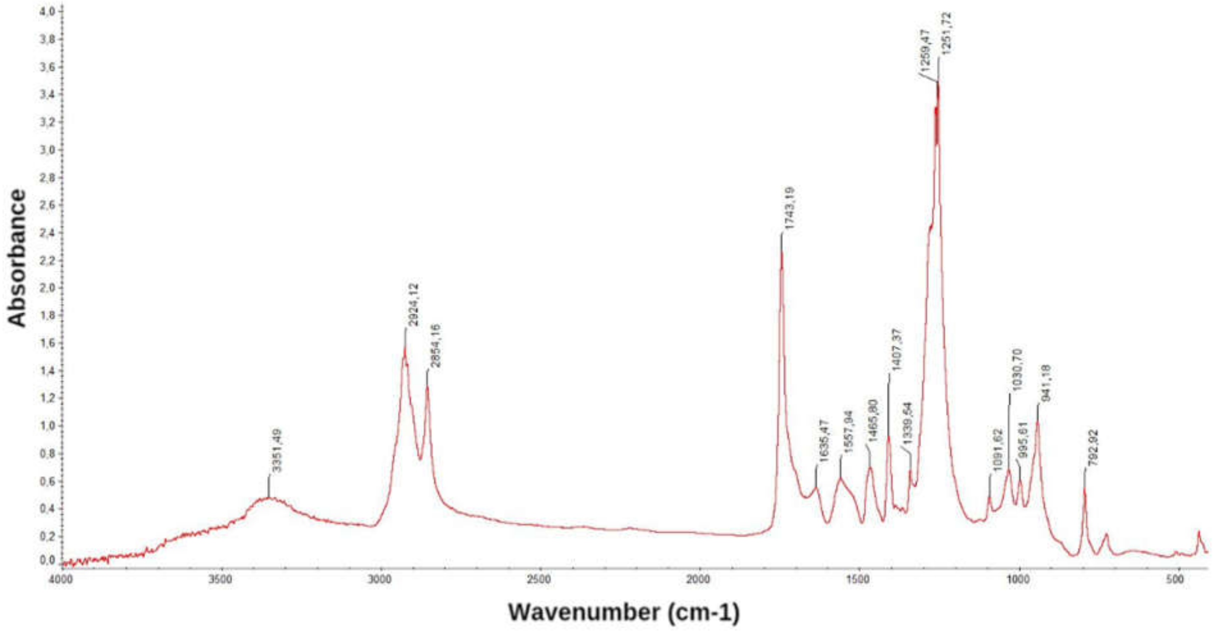

Figure S24 FTIR transmittance spectra of PCUU3000_3 poly(carbonate-urea-urethane).

E_PCUU3000_2.5

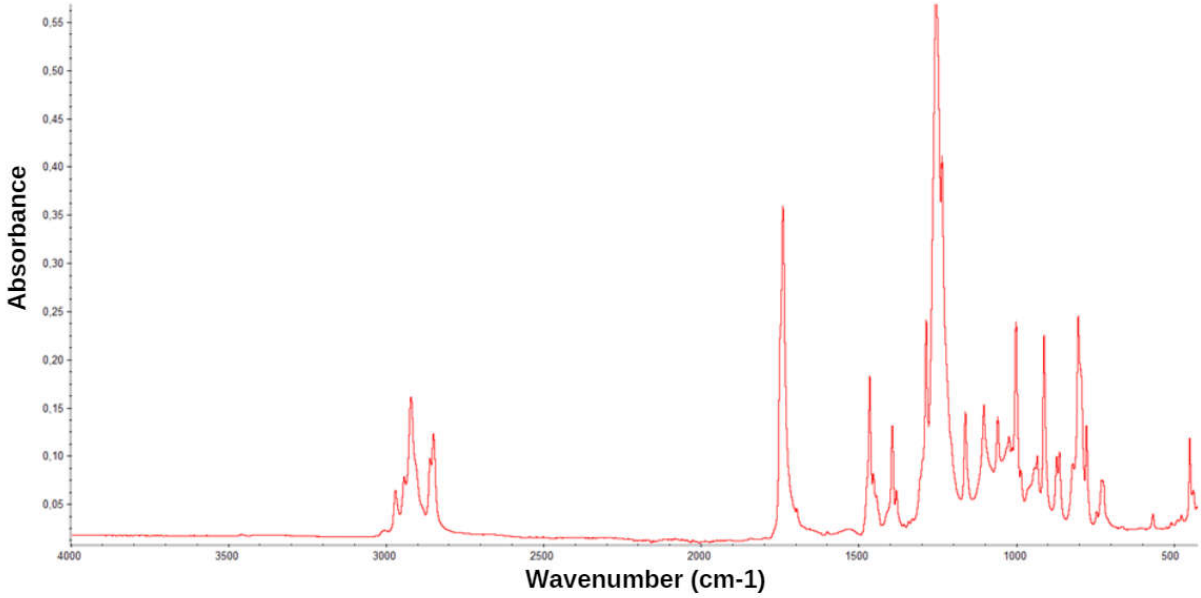

Figure S25 FTIR (ATR) spectra of PCUU3000_2.5 poly(carbonate-urea-urethane).

E_PCUU3000_2

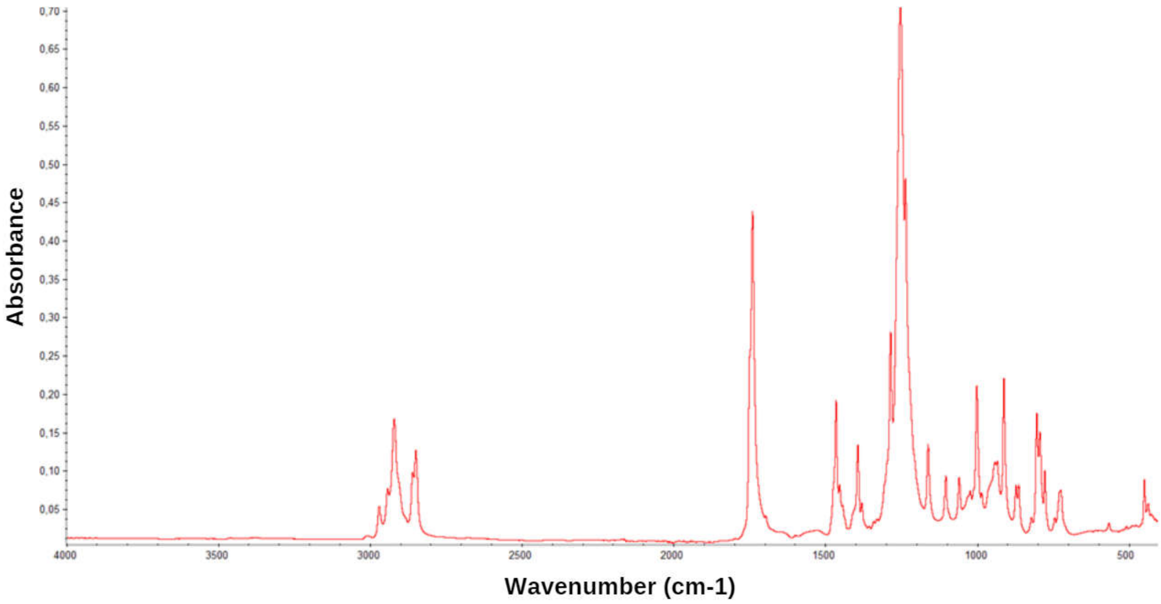

Figure S26 FTIR (ATR) spectra of PCUU3000_2 poly(carbonate-urea-urethane).

E_PCUU3000_1.5

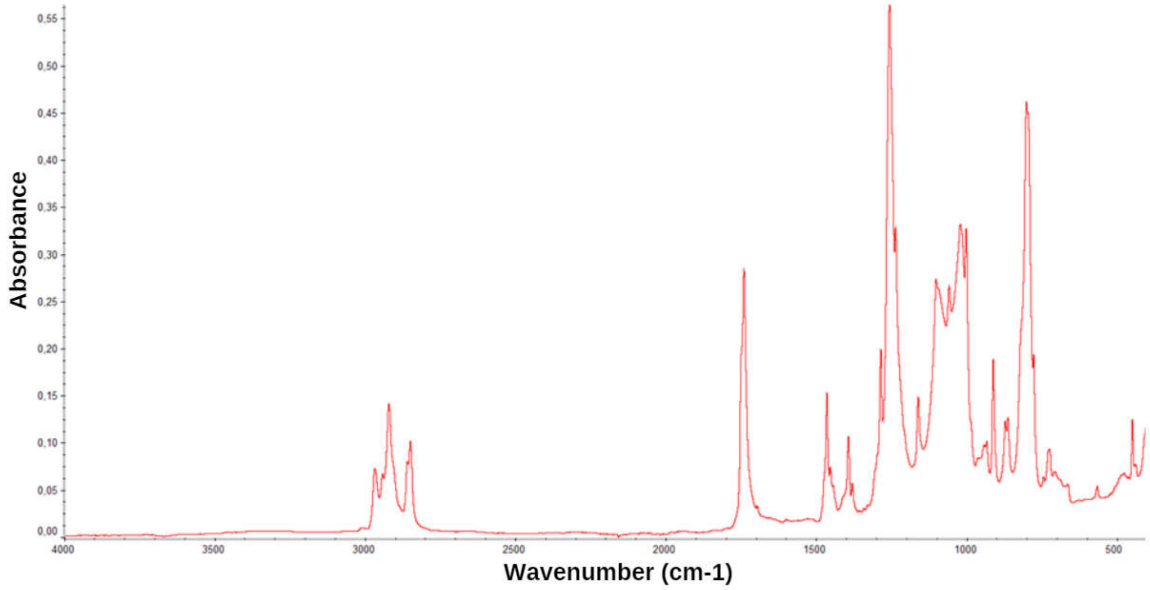

Figure S27 FTIR (ATR) spectra of PCUU3000_1.5 poly(carbonate-urea-urethane).

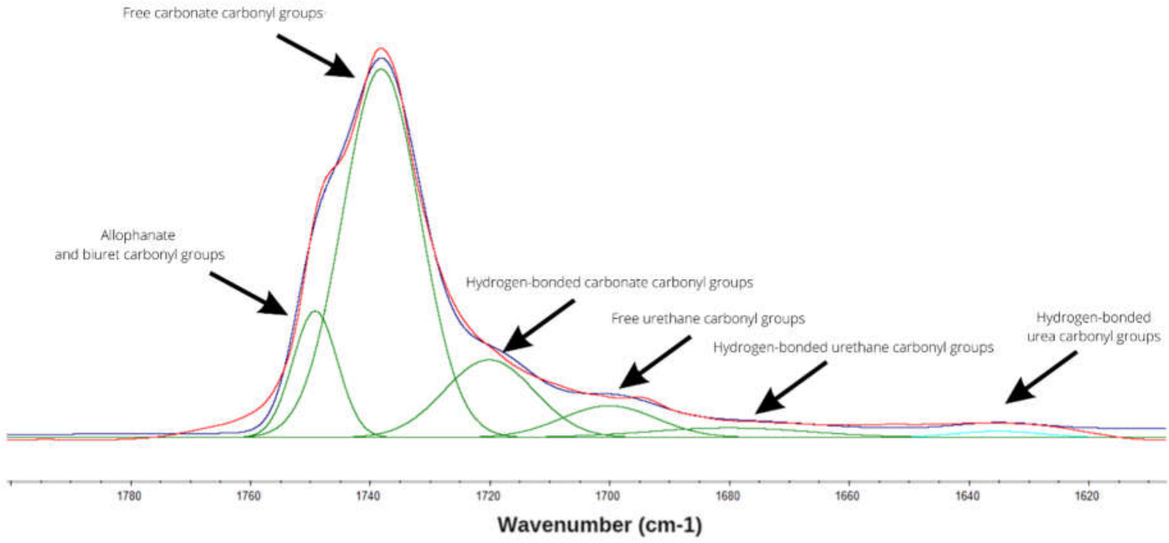

Figure S28 FTIR (ATR) Multiple absorption band distribution of E_PCUU3000_3.5.

MALDI-TOF results

E_OCD_3000

Table S1 MALDI-TOF results of the E_OCD_3000.

Pn	Pw	Pz	PD	DPn	DPw	DPz
2909	3973	4660	1,4	13,9	19,2	22,6

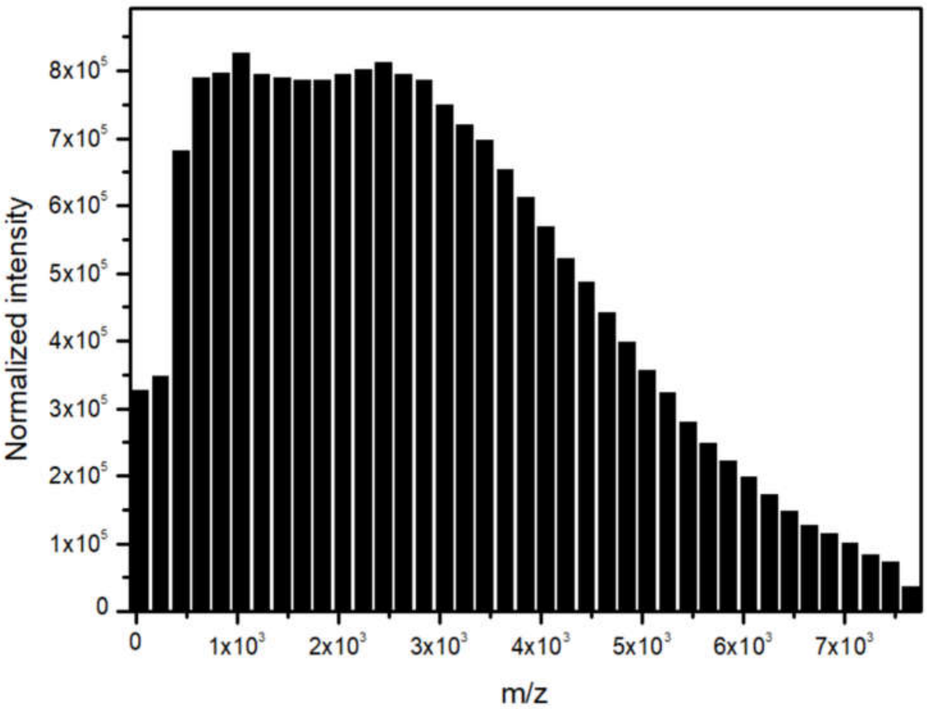

Figure S29 MALDI-TOF normalized intensity results of the E_OCD_3000.

Shape memory properties

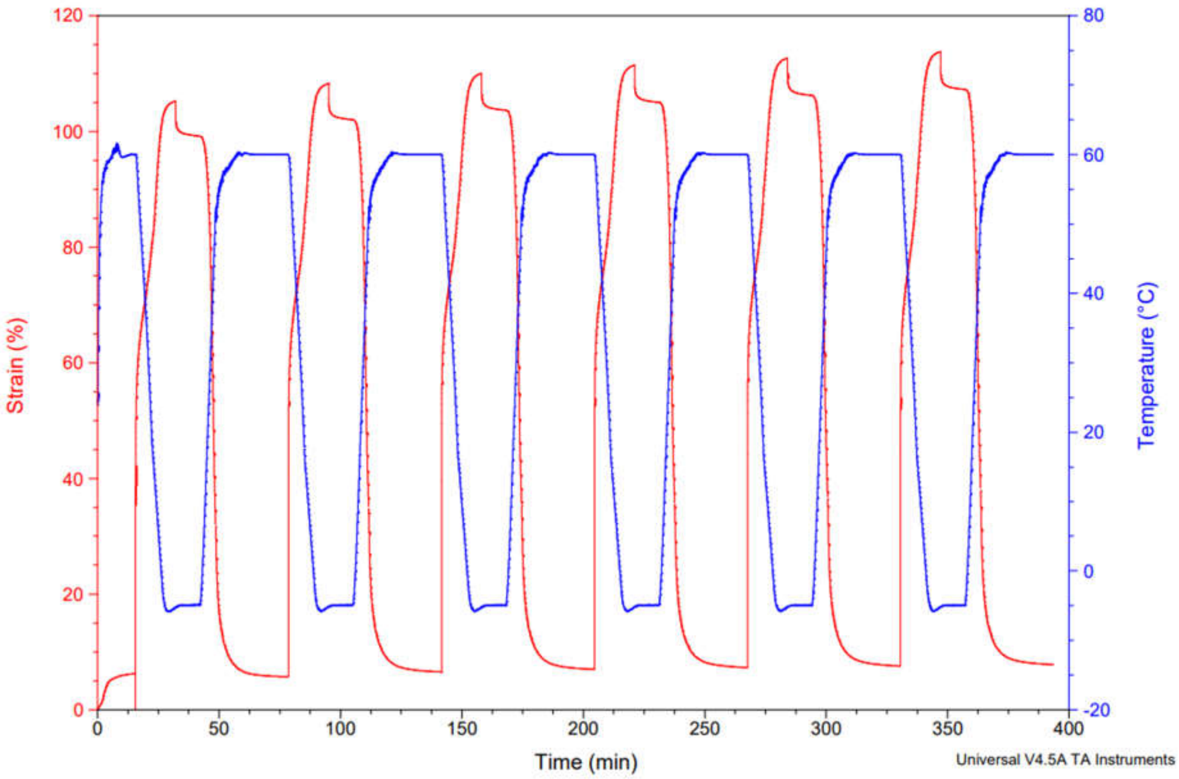

Figure S30 Thermomechanical SME measurement during 5 cycles for PCUU3000_3.5 poly(carbonate-urea-urethane)s (5N).

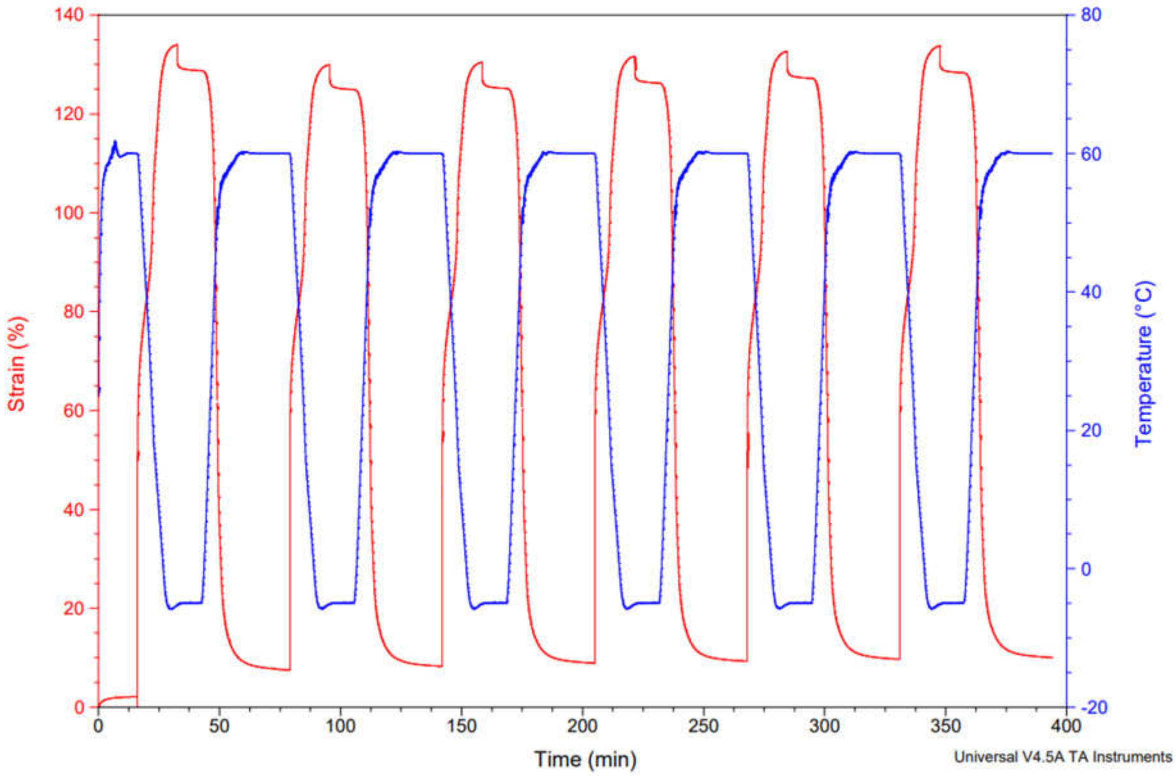

Figure S31 Thermomechanical SME measurement during 5 cycles for PCUU3000_3 poly(carbonate-urea-urethane)s (5N).

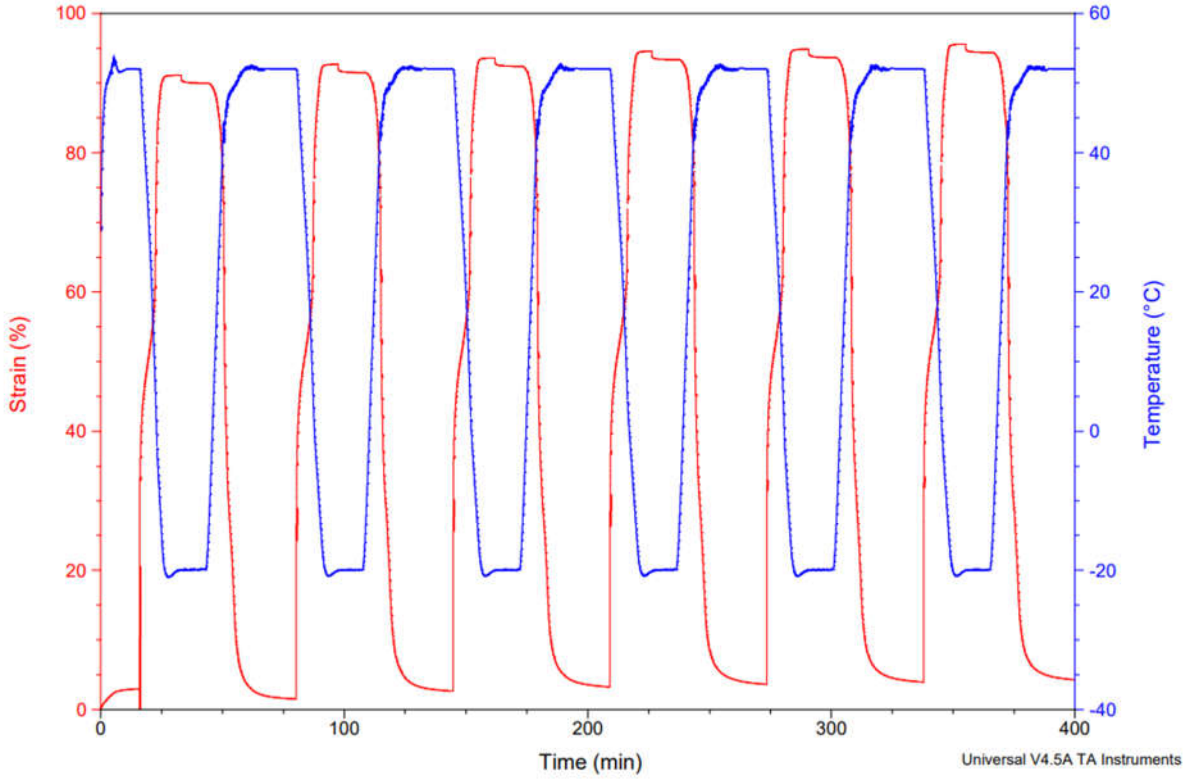

Figure S32 Thermomechanical SME measurement during 5 cycles for PCUU3000_2.5 poly(carbonate-urea-urethane)s (2N).

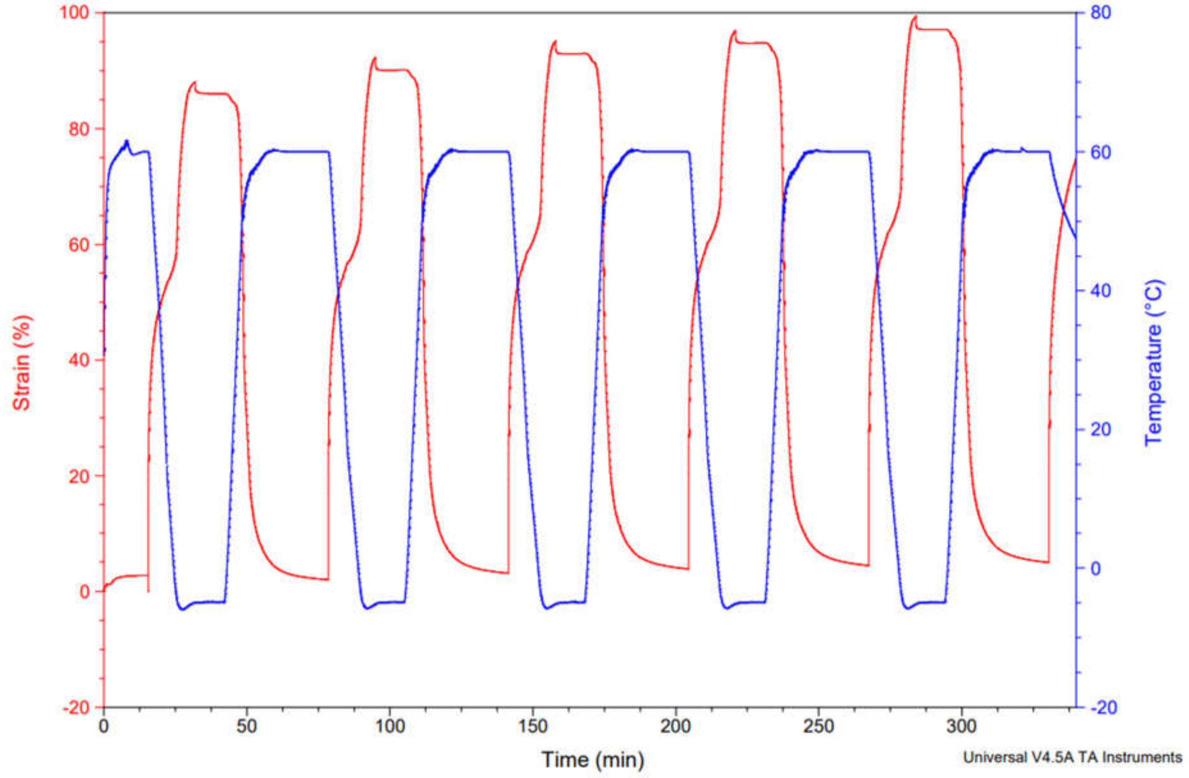

Figure S33 Thermomechanical SME measurement during 5 cycles for PCUU3000_2 poly(carbonate-urea-urethane)s (1N).

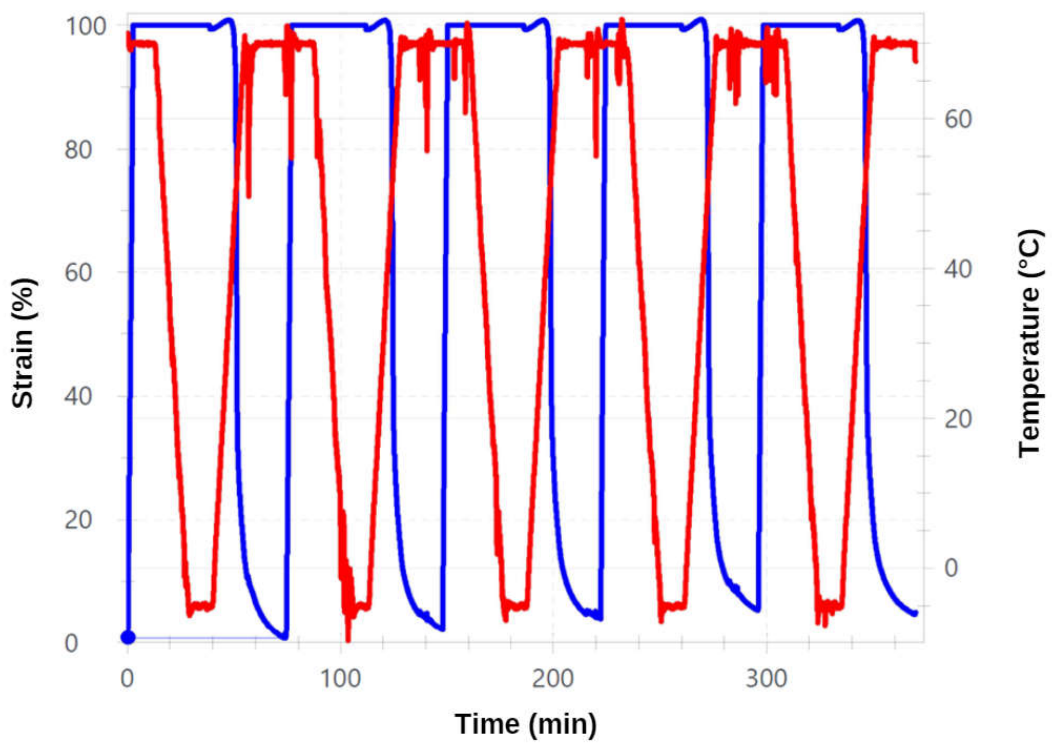

Figure S34 Thermomechanical SME measurement during 5 cycles for PCUU5000_3 poly(carbonate-urea-urethane)s (1N).

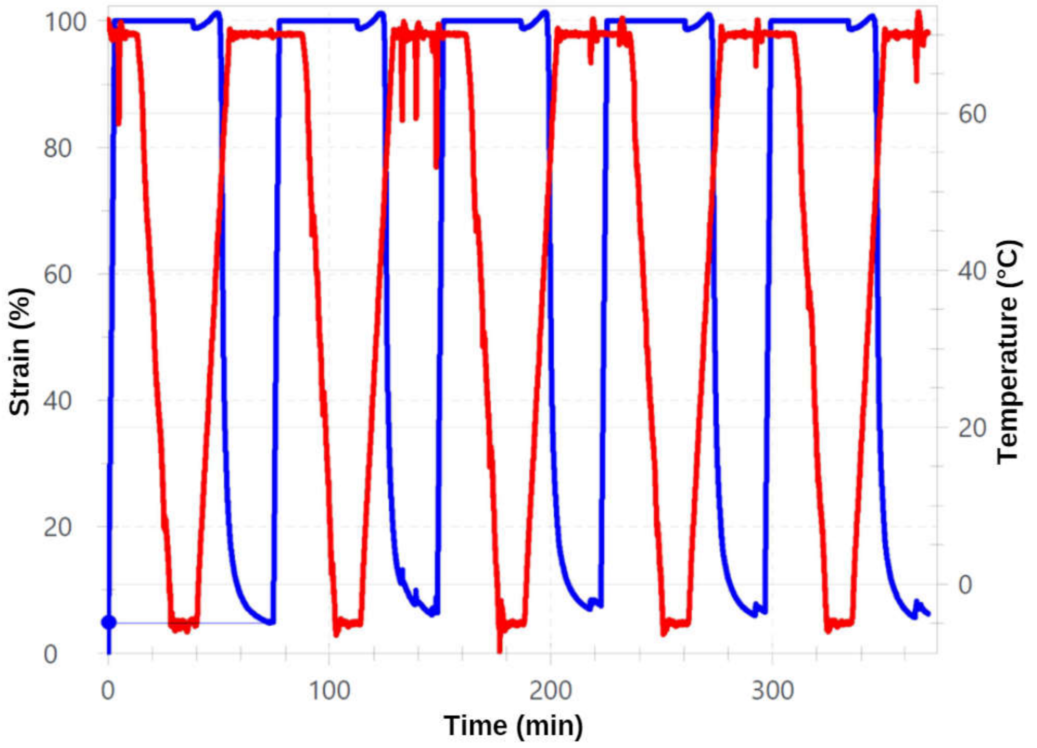

Figure S35 Thermomechanical SME measurement during 5 cycles for PCUU5000_2 poly(carbonate-urea-urethane)s (1N).

Tensile tests

E_PCUU3000_3.5

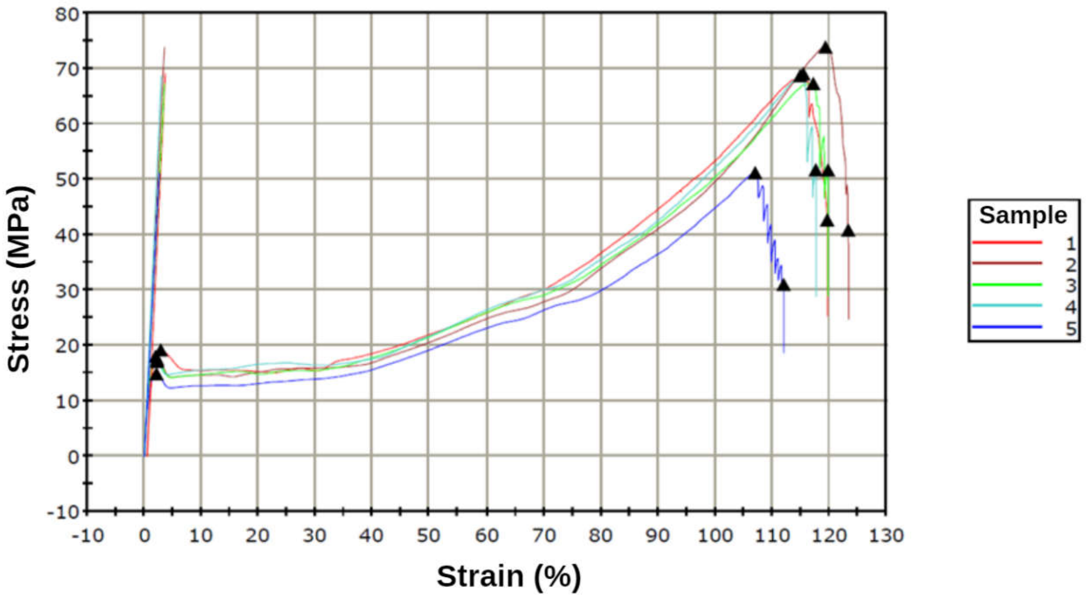

Figure S36 Stress-strain curves for E_PCUU3000_3.5 poly(carbonate-urea-urethane)s.

E_PCUU3000_3

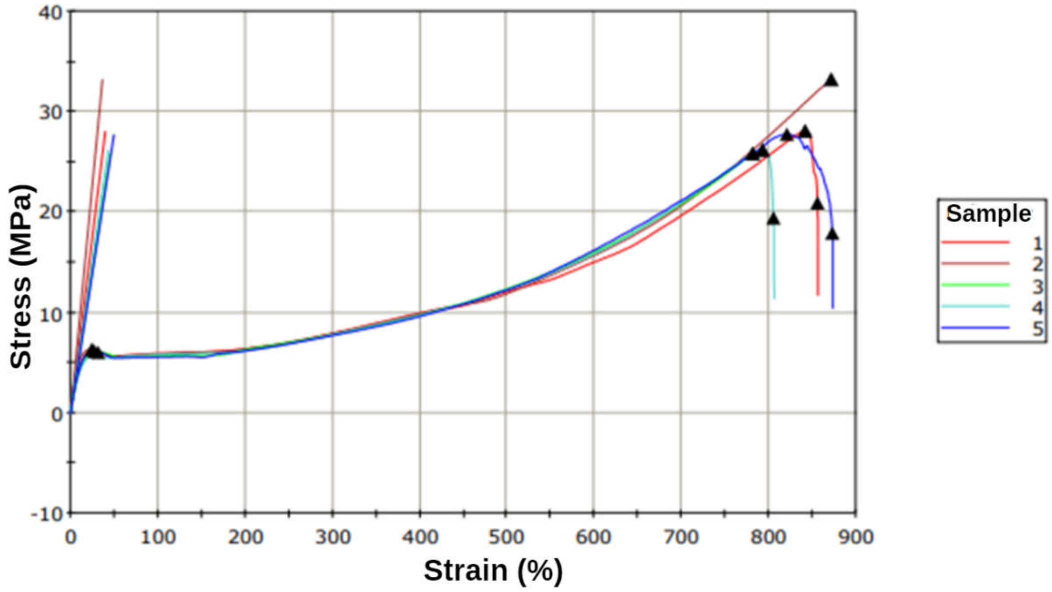

Figure S37 Stress-strain curves for E_PCUU3000_3 poly(carbonate-urea-urethane)s.

E_PCUU3000_2.5

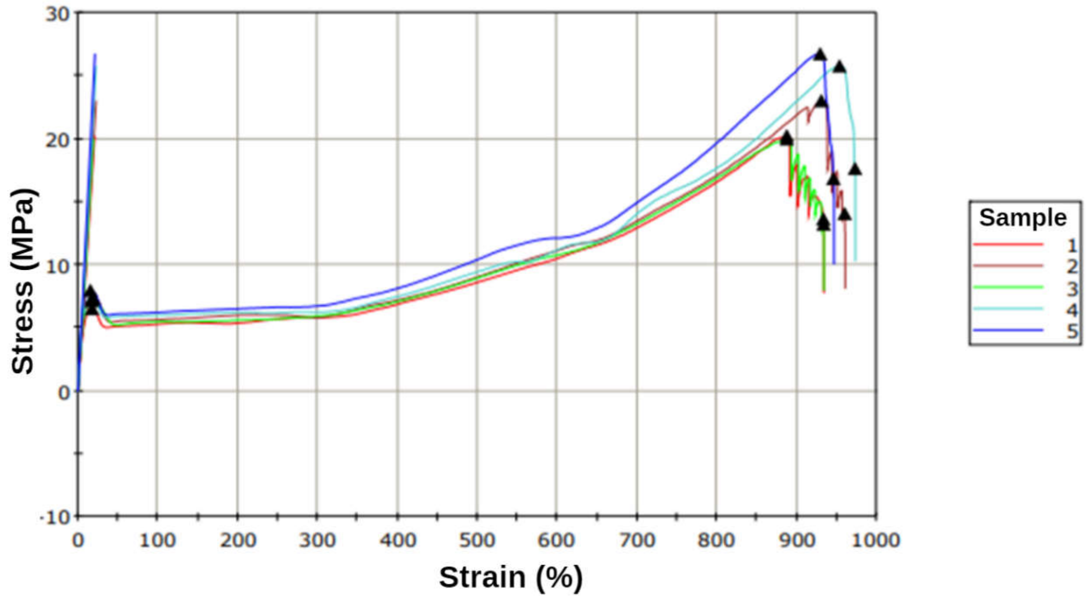

Figure S38 Stress-strain curves for E_PCUU3000_2.5 poly(carbonate-urea-urethane)s.

E_PCUU3000_2

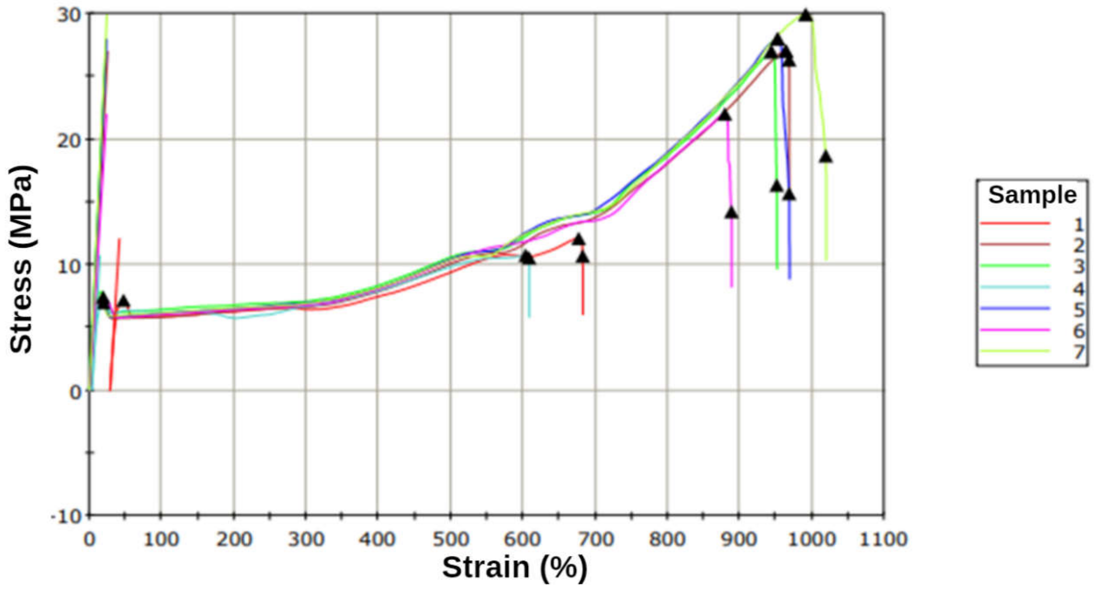

Figure S39 Stress-strain curves for E_PCUU3000_2 poly(carbonate-urea-urethane)s.

E_PCUU3000_1.5

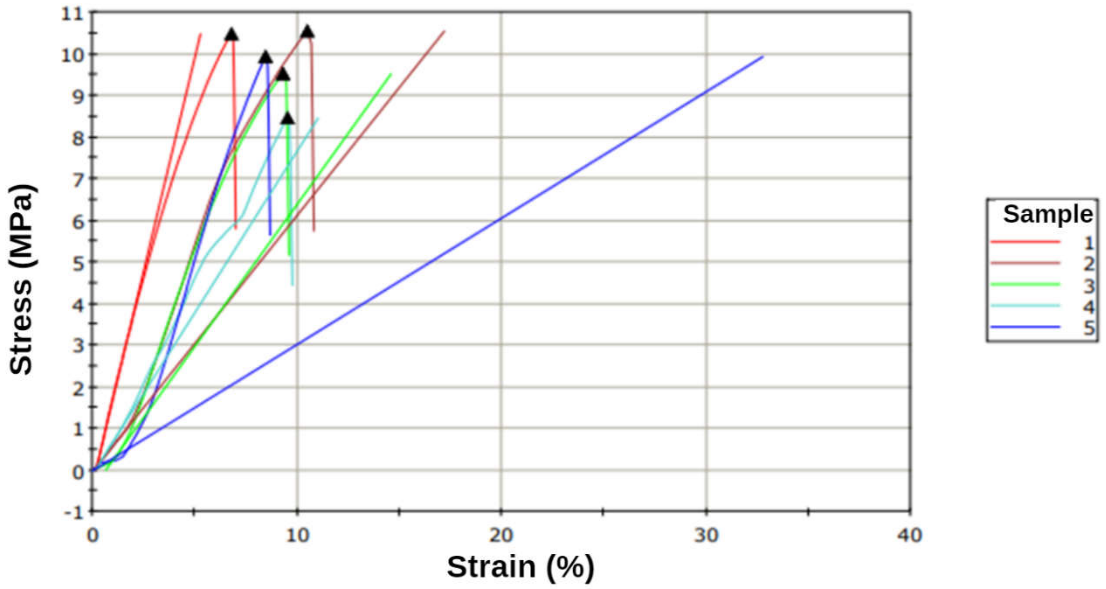

Figure S40 Stress-strain curves for E_PCUU3000_1.5 poly(carbonate-urea-urethane)s.

E_PCUU5000_3

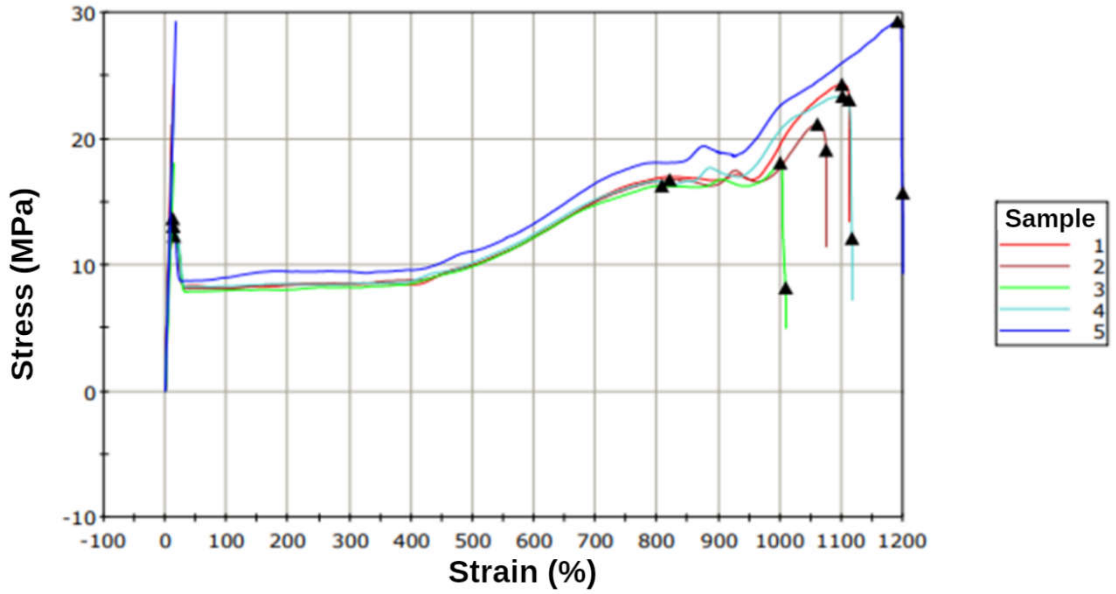

Figure S41 Stress-strain curves for E_PCUU5000_3 poly(carbonate-urea-urethane)s.

E_PCUU5000_2

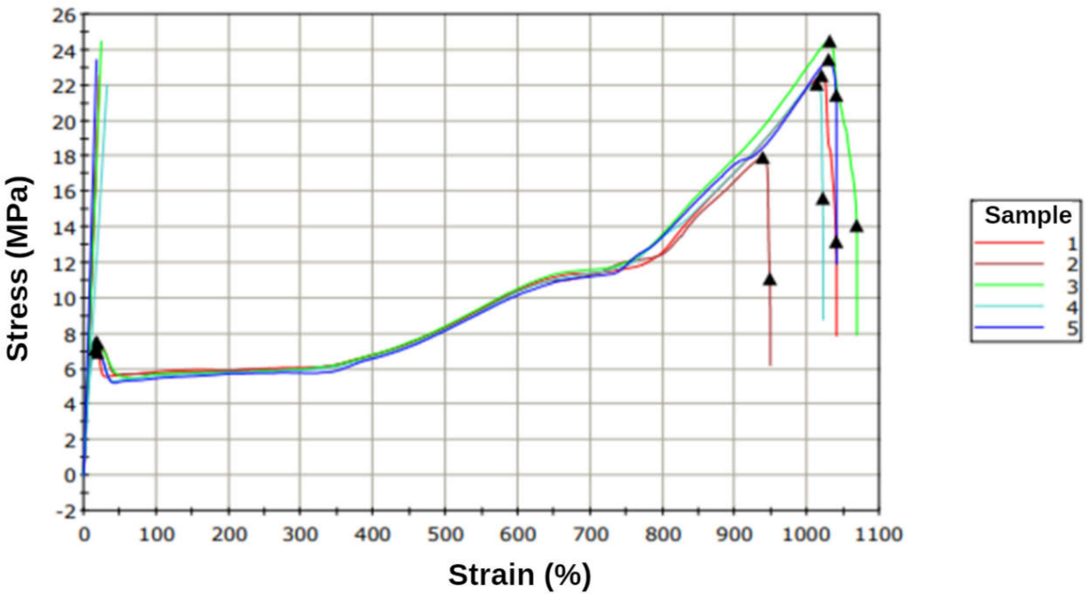

Figure S42 Stress-strain curves for E_PCUU5000_2 poly(carbonate-urea-urethane)s.

Thermal analysis

Differential scanning calorimetry (DSC)

E_PCUU3000_3.5

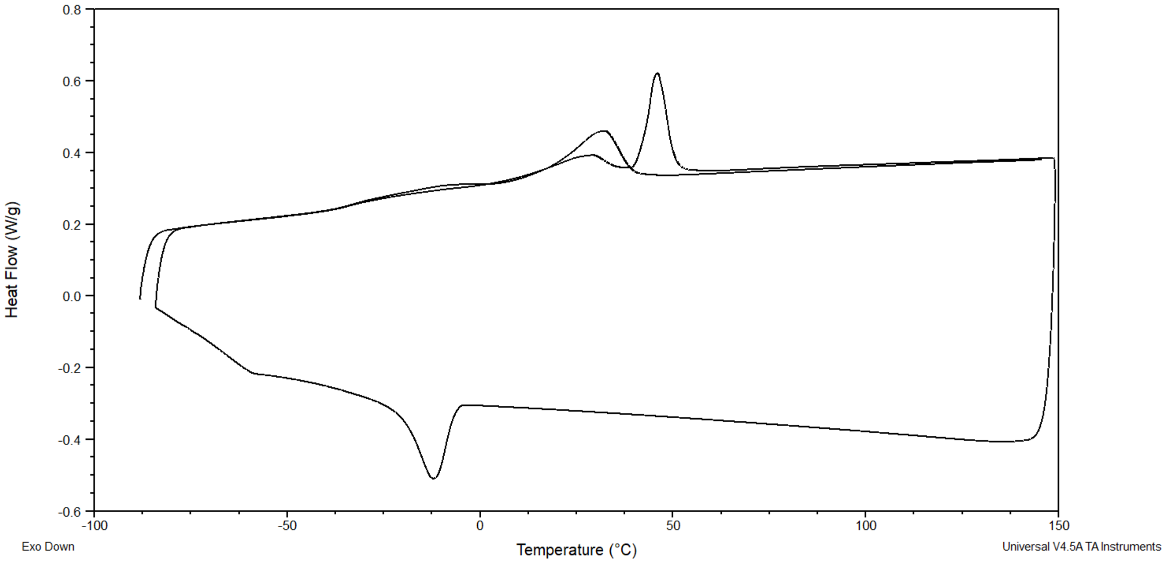

Figure S43 Heat Flow-Temperature curves for E_PCUU3000_3.5 poly(carbonate-urea-urethane).

E_PCUU3000_3

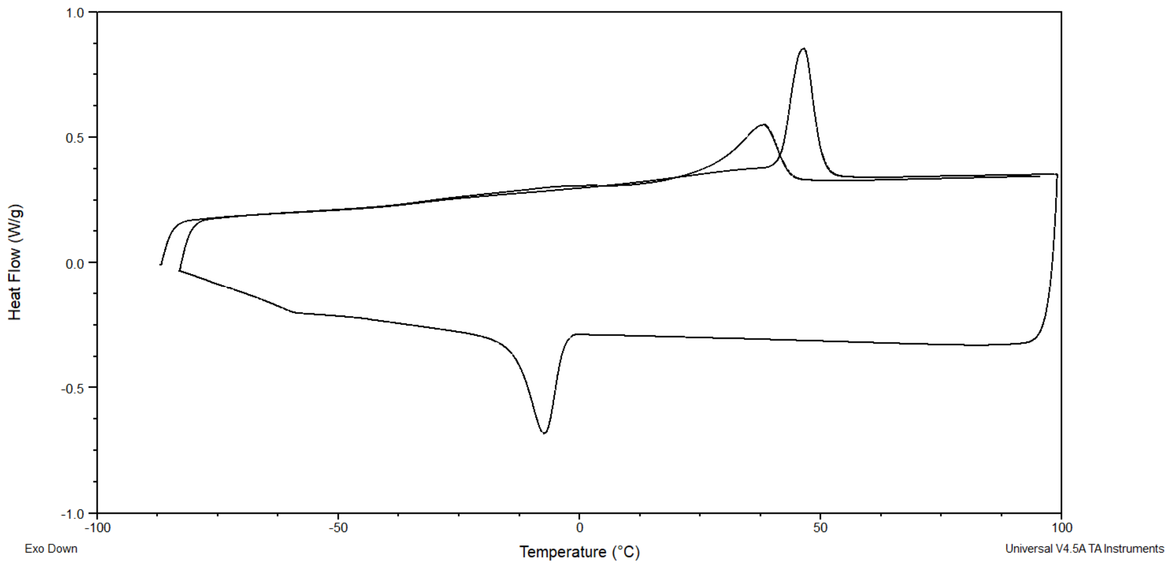

Figure S44 Heat Flow-Temperature curves for E_PCUU3000_3 poly(carbonate-urea-urethane).

E_PCUU3000_2.5

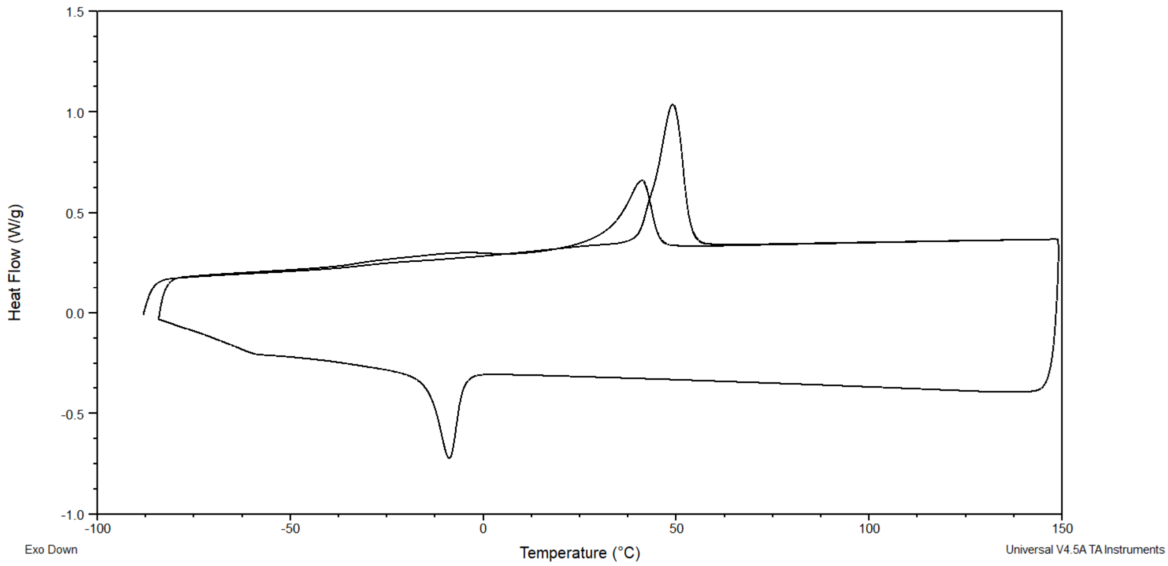

Figure S45 Heat Flow-Temperature curves for E_PCUU3000_2.5 poly(carbonate-urea-urethane).

E_PCUU3000_2

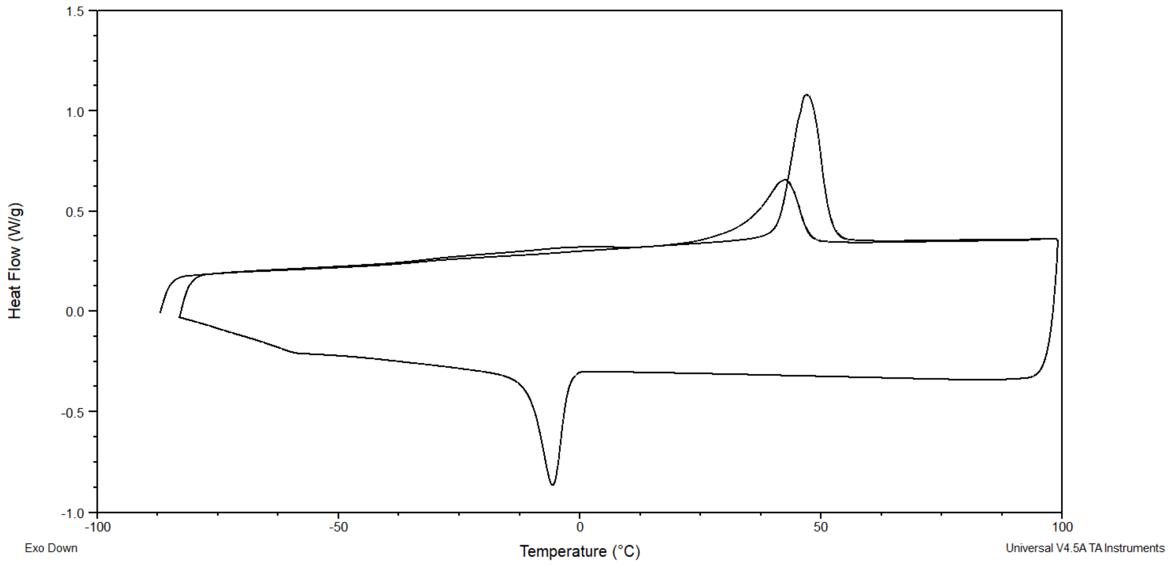

Figure S46 Heat Flow-Temperature curves for E_PCUU3000_2 poly(carbonate-urea-urethane).

E_PCUU3000_1.5

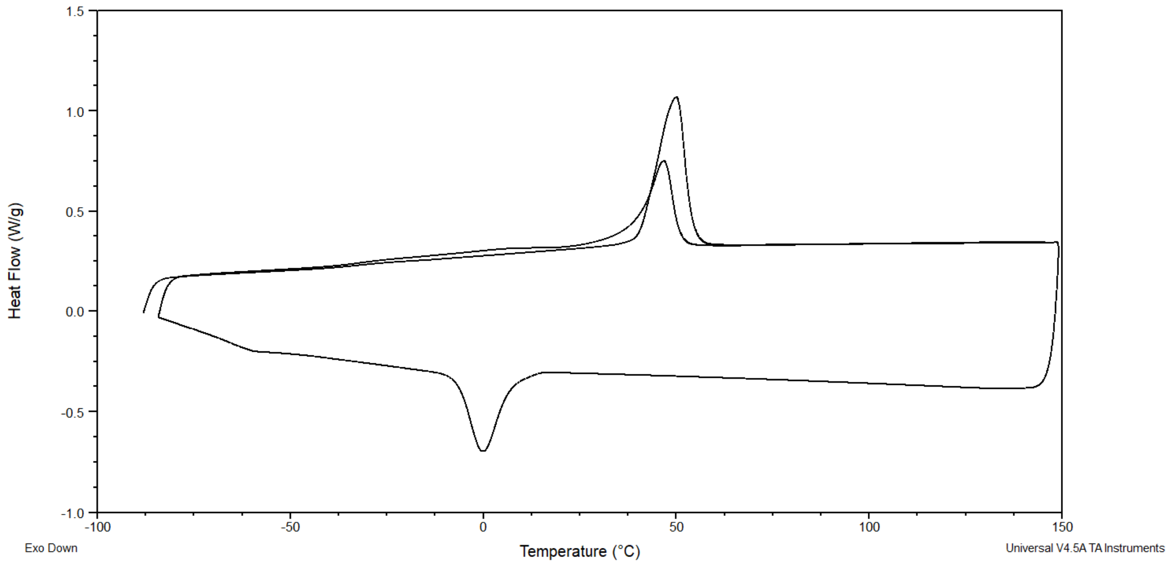

Figure S47 Heat Flow-Temperature curves for E_PCUU3000_1.5 poly(carbonate-urea-urethane).

E_PCUU3000_1.2

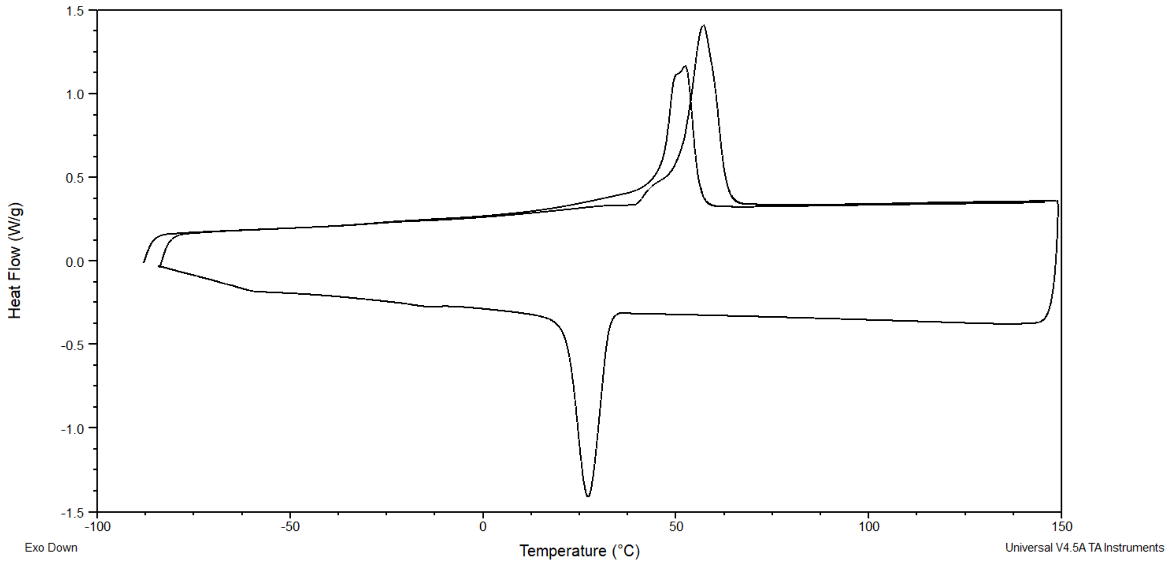

Figure S48 Heat Flow-Temperature curves for E_PCUU3000_1.2 poly(carbonate-urea-urethane).

E_PCUU5000_3

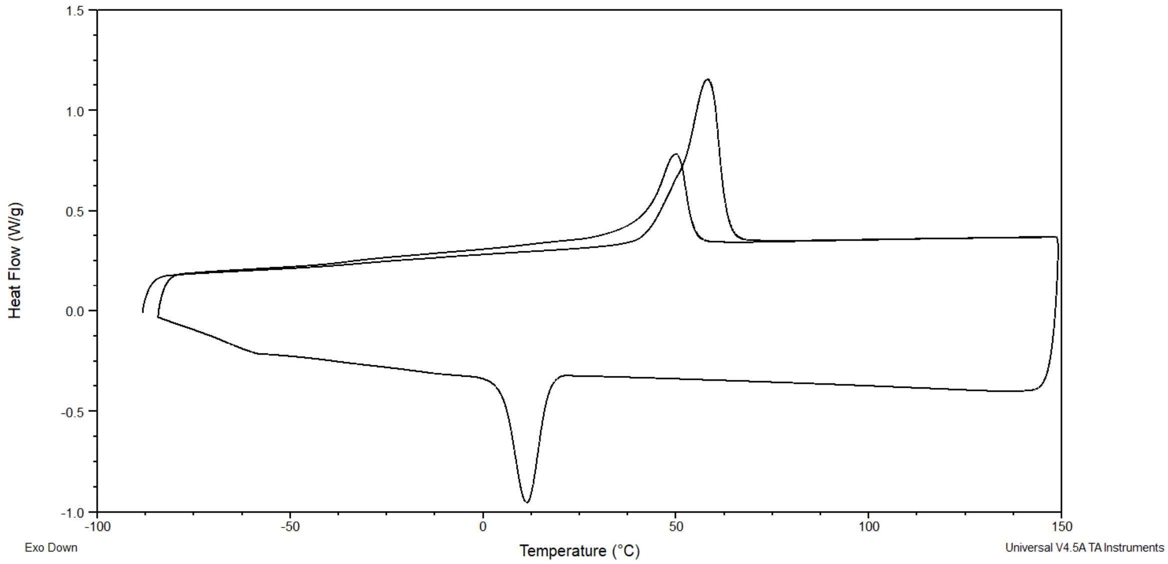

Figure S49 Heat Flow-Temperature curves for E_PCUU5000_3 poly(carbonate-urea-urethane).

E_PCUU5000_2

X-axis – Heat Flow (W/g);

Y-axis – Temperature (°C)

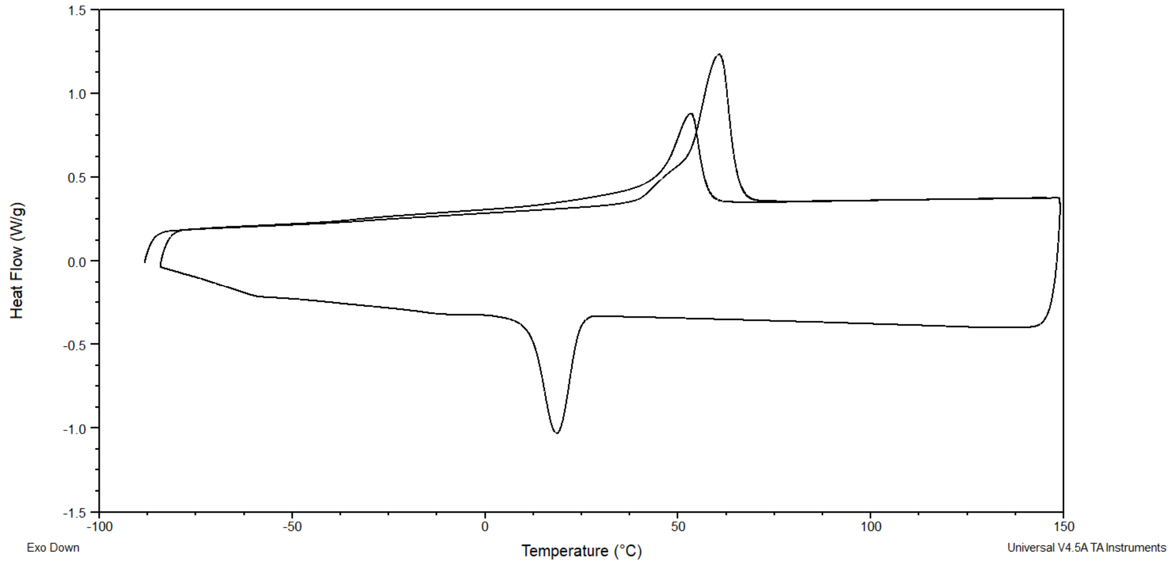

Figure S50 Heat Flow-Temperature curves for E_PCUU5000_2 poly(carbonate-urea-urethane).

E_OCD_3000

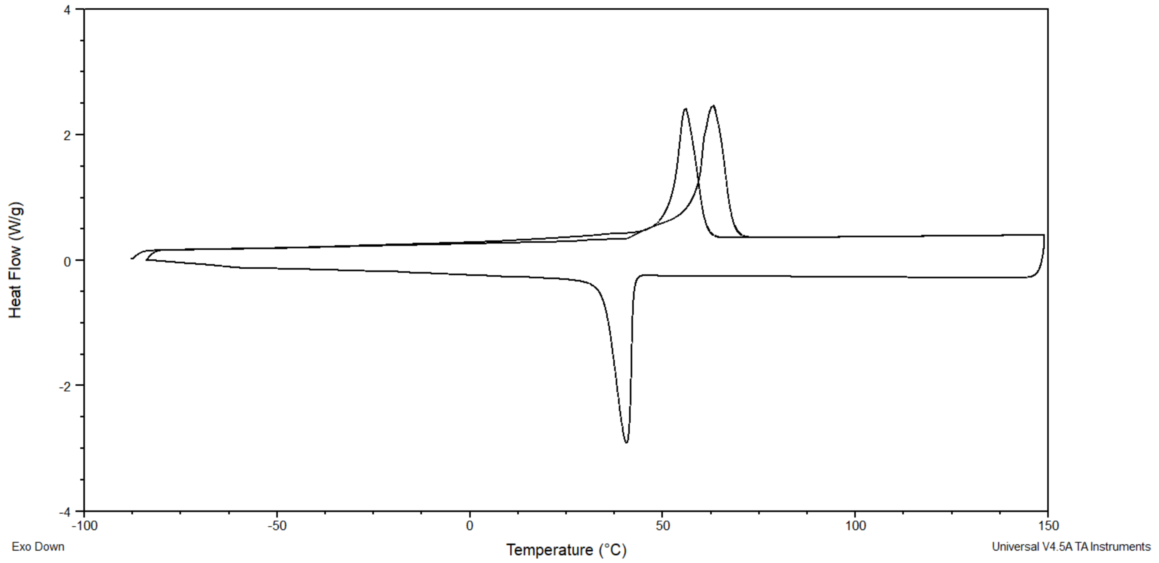

Figure S51 Heat Flow-Temperature curves for E_OCD_3000 oligocarbonate.

E_OCD_5000

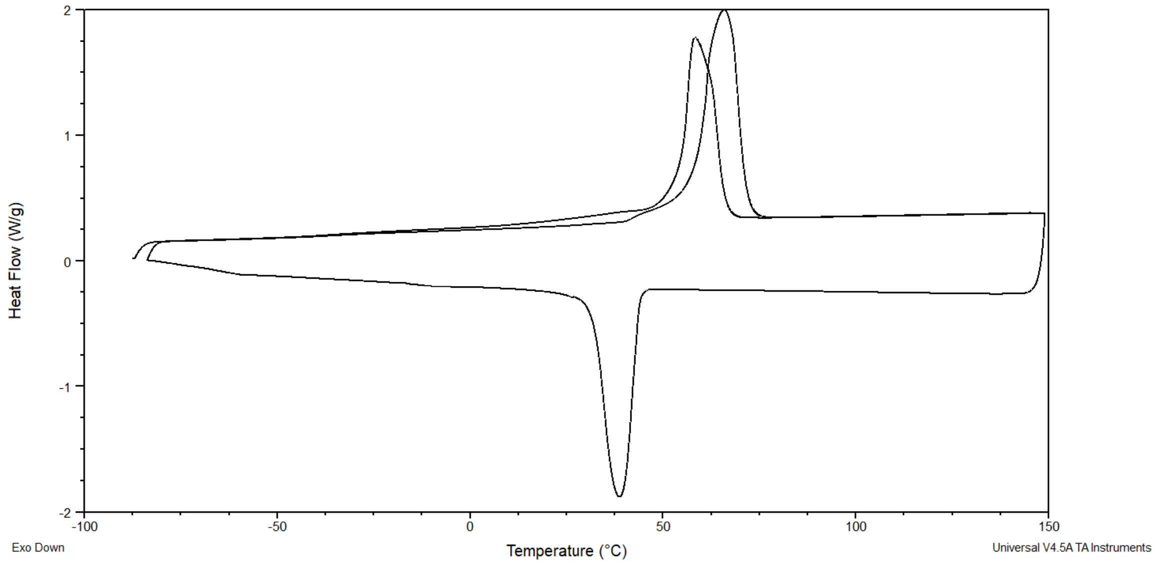

Figure S52 Heat Flow-Temperature curves for E_OCD_5000 oligocarbonate.

E_BMC_8

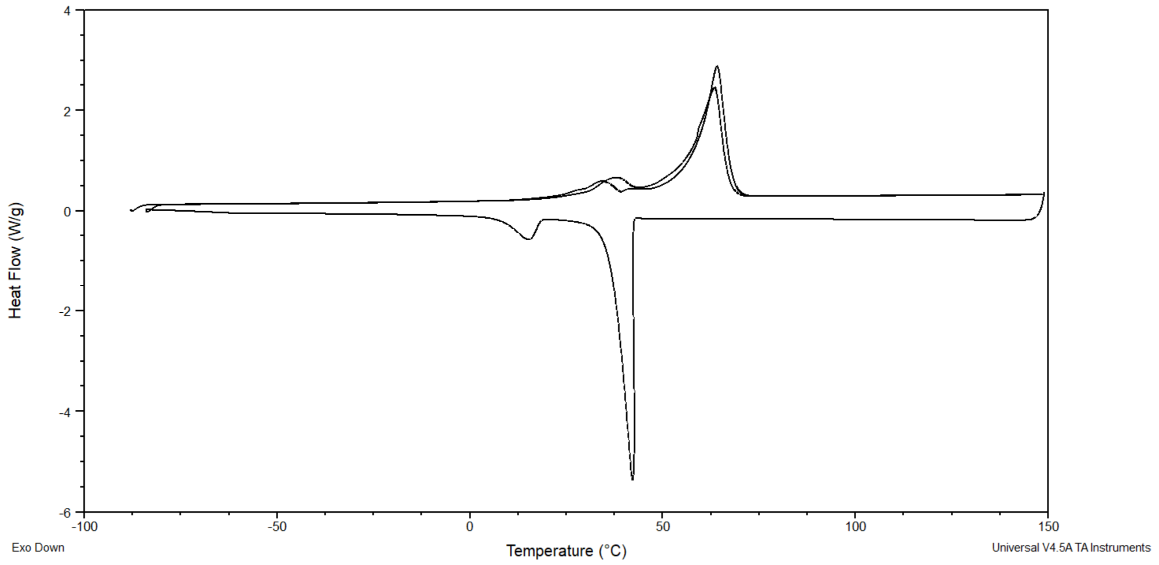

Figure S53 Heat Flow-Temperature curves for E_BMC_8 bis(methylcarbonate).

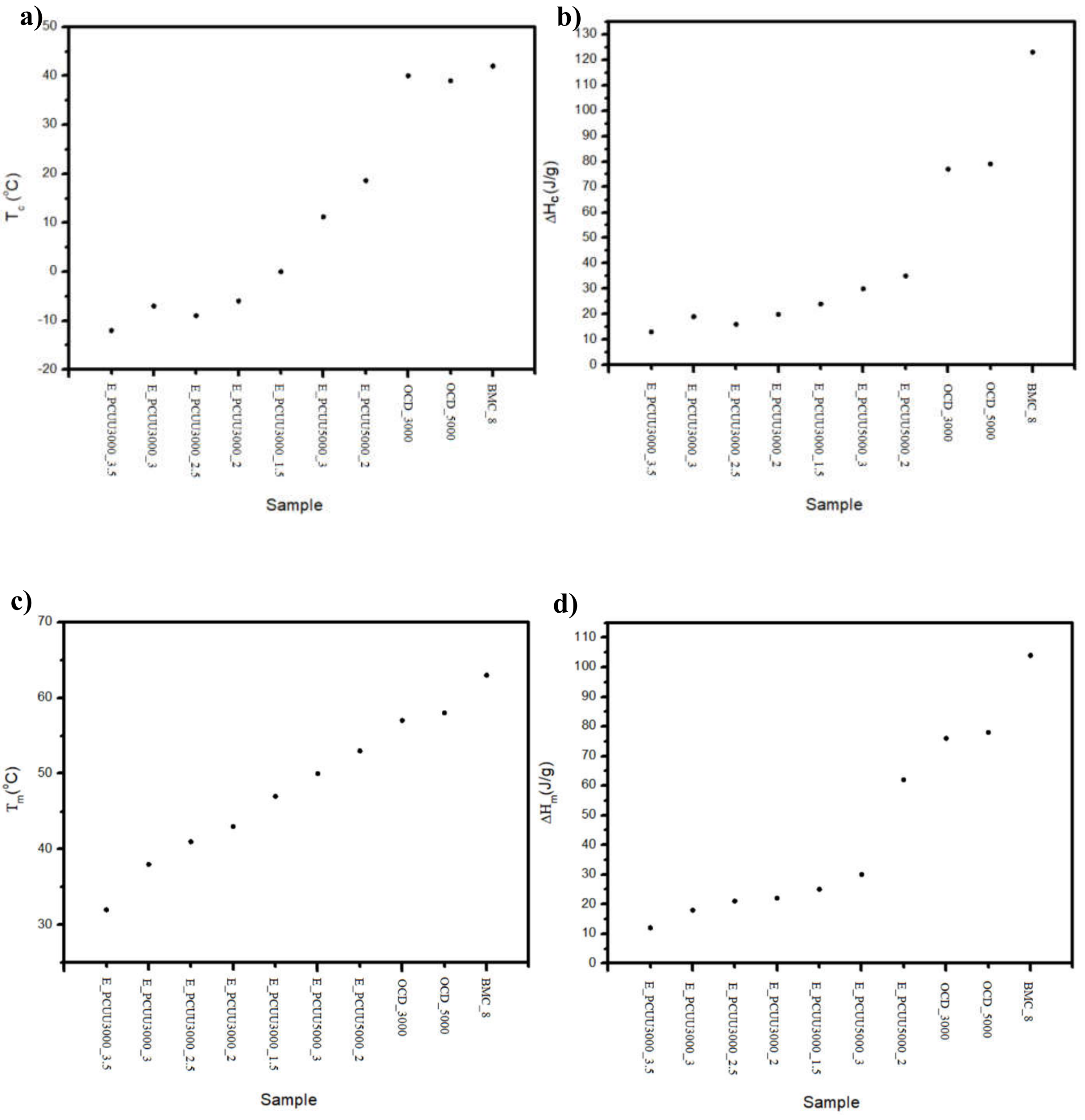

Figure S54 Thermal properties estimated from DSC measurements of poly(carbonate-urethanes-urea)s, oligocarbonate diol and alkylene bis(methylcarbonate): a) crystallization temperatures; b) crystallization enthalpies ; c) melting points; d) melting enthalpies.

Dynamic mechanical thermal analyses (DMTA)

E_PCUU3000_3.5

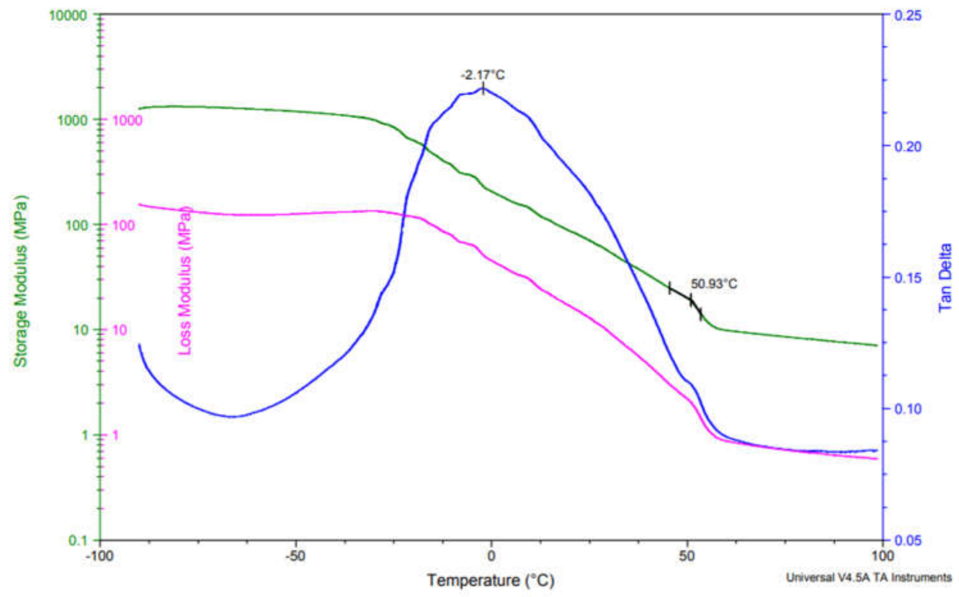

Figure S55 Dynamic mechanical thermal analysis for E_PCUU3000_3.5.

E_PCUU3000_3

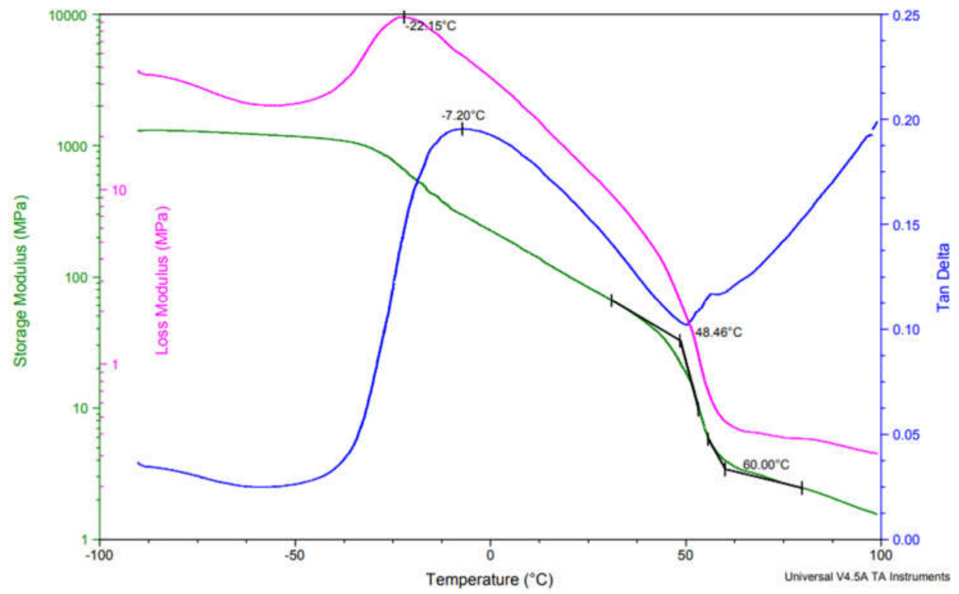

Figure S56 Dynamic mechanical thermal analysis for E_PCUU3000_3.

E_PCUU3000_2.5

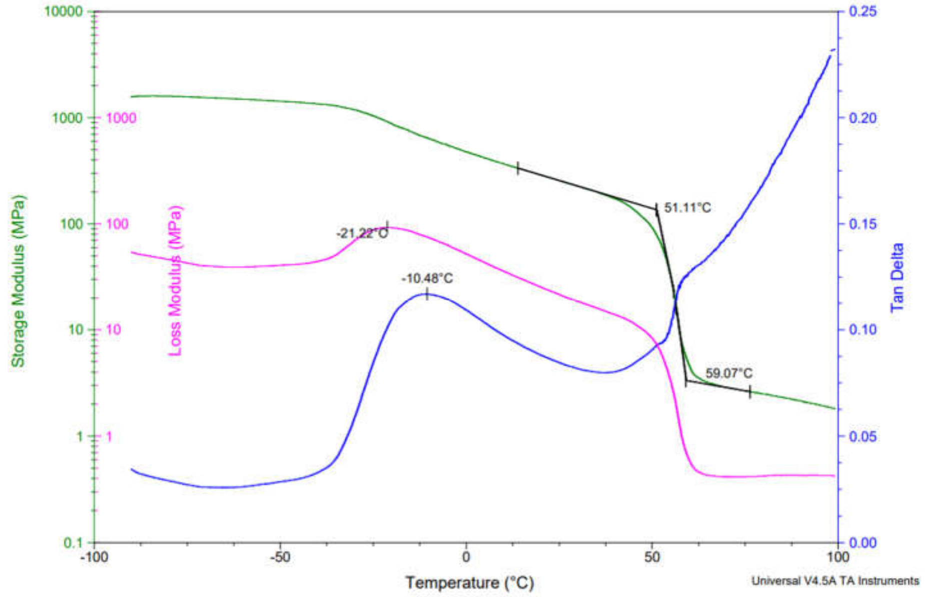

Figure S57 Dynamic mechanical thermal analysis for E_PCUU3000_2.5.

E_PCUU3000_2

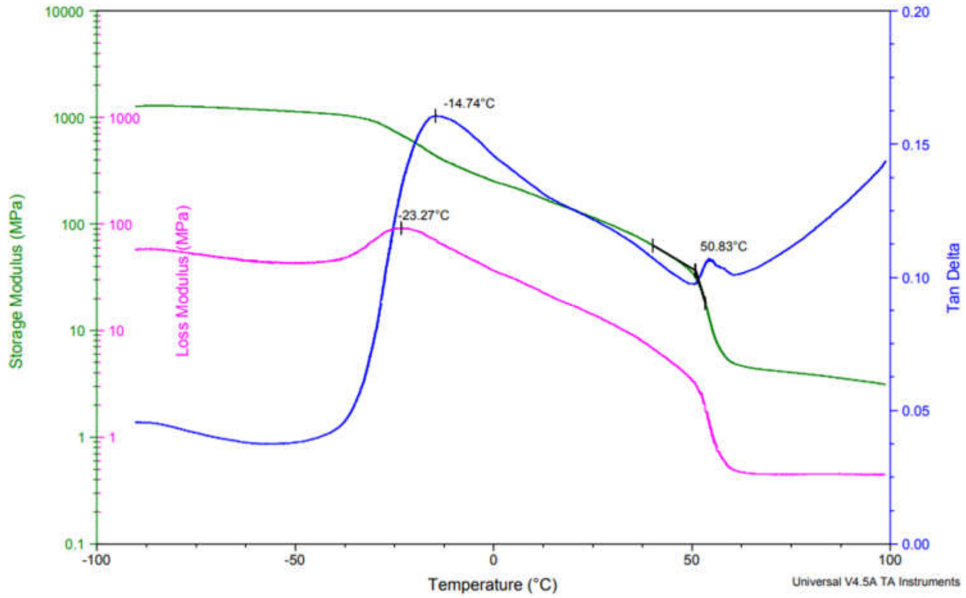

Figure S58 Dynamic mechanical thermal analysis for E_PCUU3000_2.

E_PCUU3000_1.5

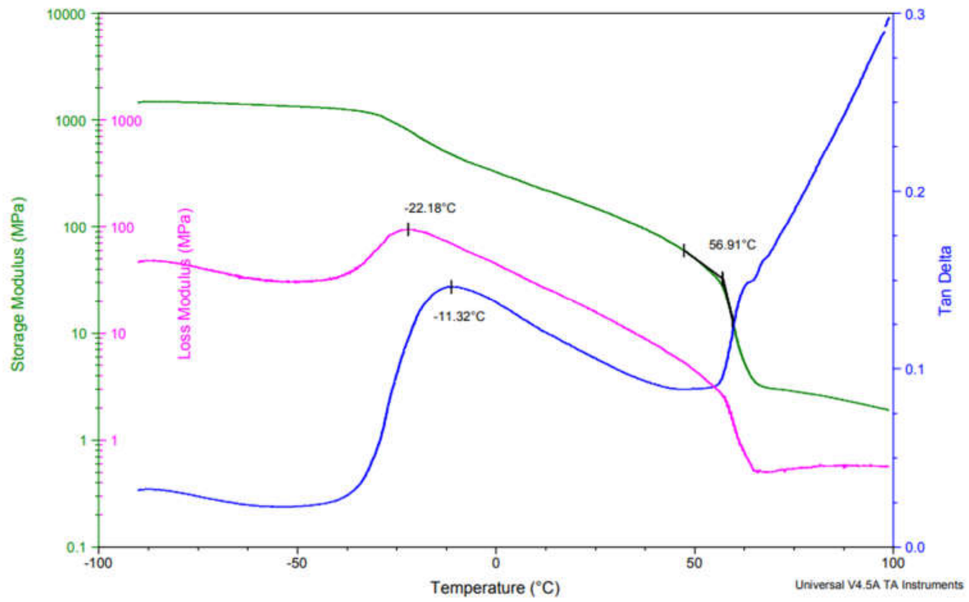

Figure S59 Dynamic mechanical thermal analysis for E_PCUU3000_1.5.

E_PCUU5000_3

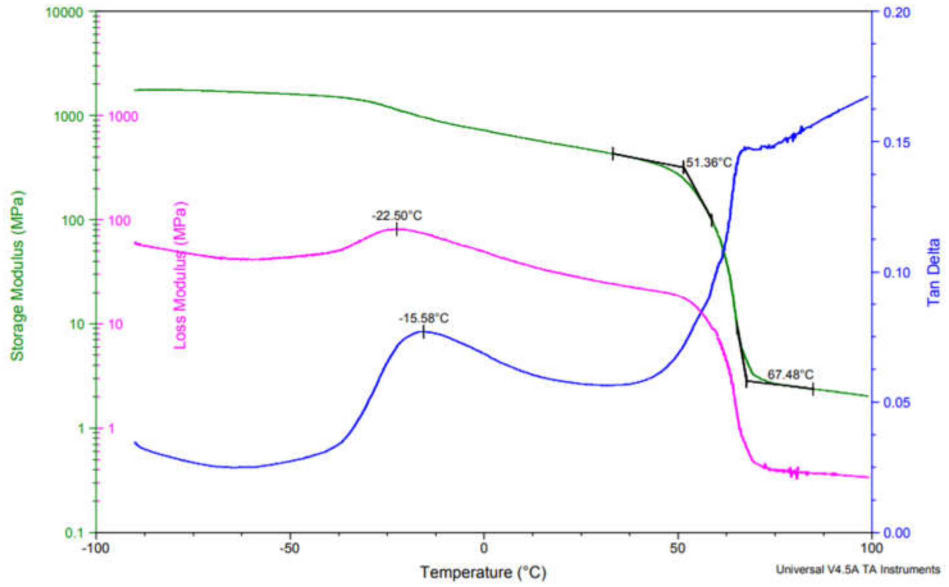

Figure S60 Dynamic mechanical thermal analysis for E_PCUU5000_3.

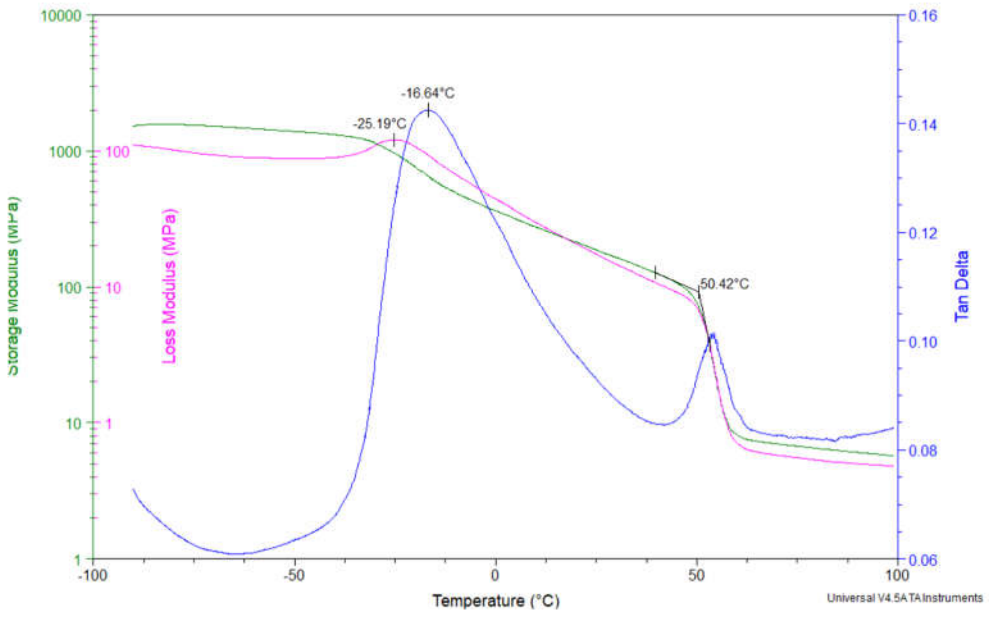

Figure S61 Dynamic mechanical thermal analysis for E_PCUU5000_2.

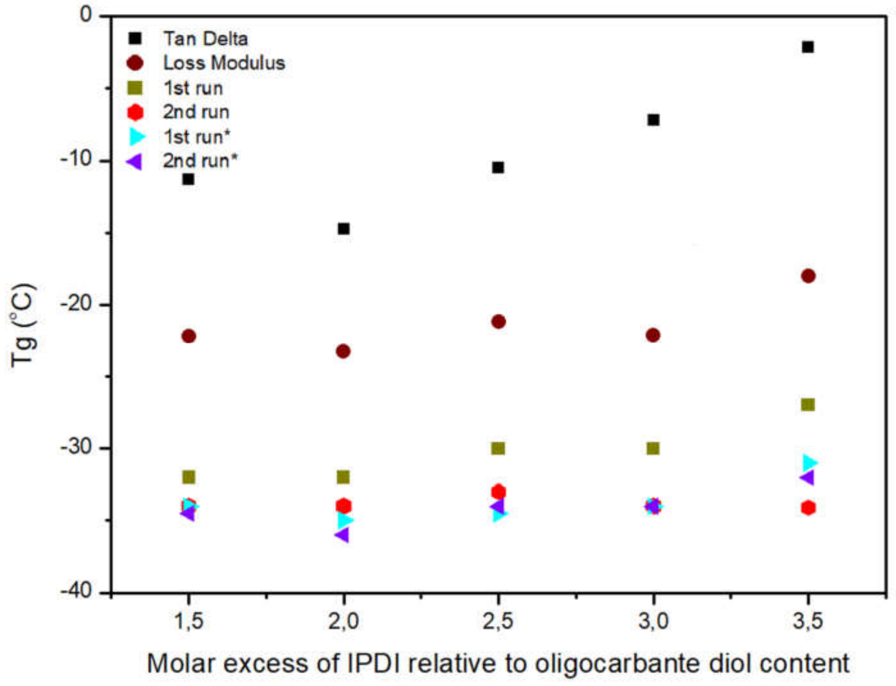

Figure S62 Glass transition temperatures estimated from DMA and MDSC measurements for PCUUs based on 3000g/mol OCD.

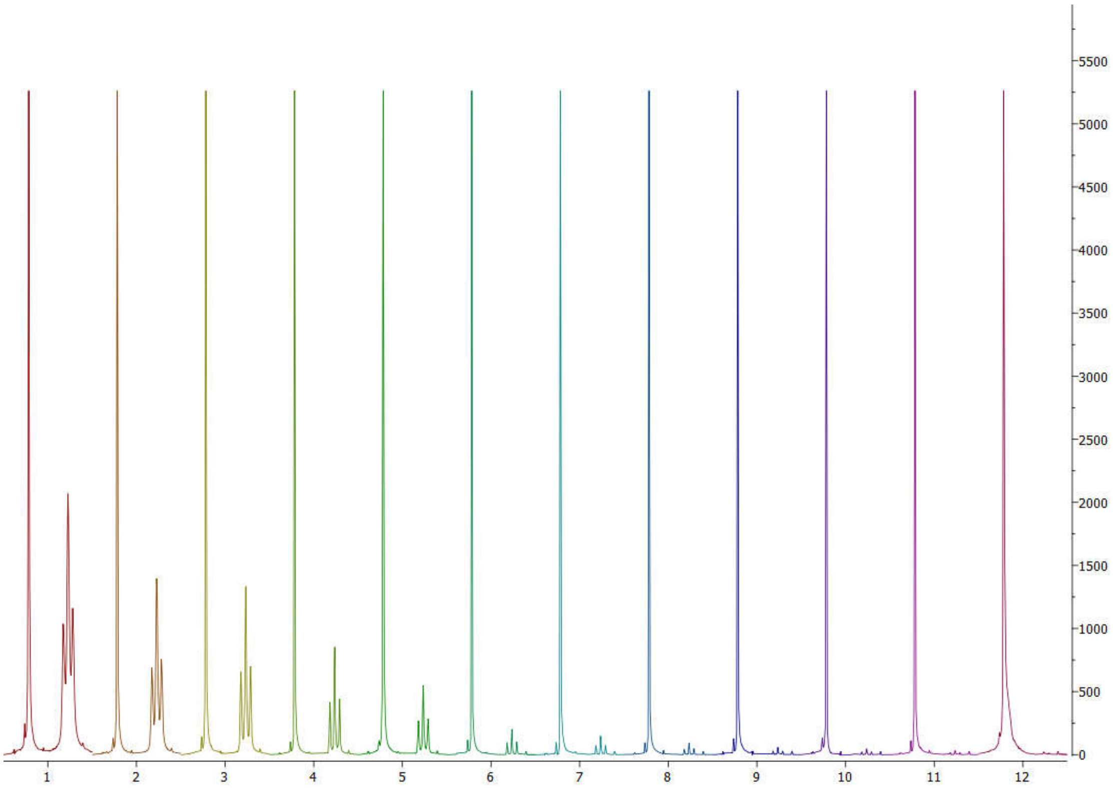

Figure S63 ^{1}H NMR stack of spectra for the region from 3.85 ppm to 3.55 ppm illustrating the progress of conversion for E_BCM_10 with 20 minutes sampling intervals.
